# Structural basis of nucleosome remodeling by Cockayne syndrome B homologue *Komagataella phaffii* Rad26

**DOI:** 10.1038/s41467-026-73500-7

**Published:** 2026-06-24

**Authors:** Yutaro Fukushima, Chiaki Kinoshita, Lumi Negishi, Tomoya Kujirai, Yuki Kobayashi, Mitsuo Ogasawara, Haruhiko Ehara, Shun-ichi Sekine, Wataru Kagawa, Hitoshi Kurumizaka, Yoshimasa Takizawa

**Affiliations:** 1 Laboratory of Chromatin Structure and Function, Institute for Quantitative Biosciences, The University of Tokyo, Tokyo, Japan; 2 Department of Biological Sciences, Graduate School of Science, The University of Tokyo, Tokyo, Japan; 3 Department of Chemistry, Graduate School of Science and Engineering, Meisei University, Tokyo, Japan; 4 RIKEN Center for Integrative Medical Sciences, Yokohama, Japan; 5 Division of Chromatin Structure and Function, Department of Multidisciplinary Life Science, Medical Institute of Bioregulation, Kyushu University, Fukuoka, Japan; 6 Department of Computational Biology and Medical Sciences, Graduate School of Frontier Sciences, The University of Tokyo, Tokyo, Japan

**Keywords:** Cryoelectron microscopy, Cryoelectron microscopy, DNA damage and repair, Nucleosomes, Transcription

## Abstract

Rad26, a yeast homologue of mammalian Cockayne syndrome protein B (CSB), plays an essential role in transcription-coupled nucleotide excision repair (TC-NER). Rad26/CSB binds RNA polymerase II stalled at DNA lesions and recruits DNA repair factors, functioning as a molecular scaffold. In addition, Rad26/CSB possesses nucleosome-remodeling activity that may help restore transcription after DNA repair. Here we determine the cryo-electron microscopy structure of the Rad26/CSB-nucleosome complex. Rad26/CSB binds near the nucleosomal entry/exit region (superhelical location ±6) through a unique mechanism in which its ATPase domains, Lobe 1 and Lobe 2, engage nucleosomal DNA in a reverse orientation compared with other remodelers such as Snf2 and Ino80. Mutational, biochemical, and crosslinking mass-spectrometric analyses demonstrate the requirement of the KR loop for nucleosome binding and remodeling. Furthermore, we show that N-terminal auto-inhibition involves long-range contacts between the disordered N-terminus and the Lobe 2 region, and is relieved by mutations of Leu8 and Leu11. These findings reveal the structural basis of Rad26/CSB-mediated nucleosome remodeling in TC-NER.

## Abstract (web summary)

Transcription-coupled nucleotide excision repair requires chromatin remodeling. Here, authors determine structure of the Rad26/CSB-nucleosome, revealing a reversed ATPase orientation, while biochemical analyses define key roles of the KR loop and autoinhibitory domain.

## Introduction

Transcription-coupled nucleotide excision repair (TC-NER) is an essential DNA repair pathway that resolves DNA lesions caused by endogenous or exogenous genotoxic agents on actively transcribed genes^1–7^. These lesions stall RNA polymerase II (RNAPII) and threaten genomic stability^1,5^. The TC-NER machinery recognizes the stalled RNAPII at the DNA lesion sites and removes the DNA lesion^1–7^. The RNAPII transcription is then restored, and the DNA is repaired^6,8^.

In eukaryotes, genomic DNA is compacted as chromatin, in which nucleosomes wrap an approximately 150 base-pair DNA segment around the histone octamer composed of two of each histone, H2A, H2B, H3, and H4^9–11^. During transcription elongation, RNAPII gradually peels the nucleosomal DNA and dominantly pauses at the superhelical locations (SHLs) −5 and −1^12–14^. We previously reported that RNAPII stalls at other nucleosomal DNA locations if a DNA lesion is introduced^15^.

Rad26 is a yeast homologue of mammalian Cockayne syndrome protein B (CSB), which is responsible for Cockayne syndrome, a rare disorder characterized by developmental abnormality^3,6,8,16^. Rad26/CSB is an essential TC-NER factor and binds to the RNAPII stalled on the nucleosomal DNA by a DNA lesion. Rad26/CSB reportedly promotes the assembly of TC-NER factors on the DNA lesion site, as a scaffold for DNA repair proteins^3,6,8,17–19^. Nevertheless, Rad26/CSB belongs to a family of ATP-dependent nucleosome remodelers^20–22^. Rad26/CSB contains the Lobe 1, Lobe 2, and Brace domains, which are commonly conserved in chromatin remodelers, but is phylogenetically diverse from the four major Snf2-like, Swr1-like, Rad54-like, and Rad5/16-like families (Supplementary Fig. 1)^20^. Intriguingly, Rad26/CSB reportedly enhances the processivity of RNAPII in the nucleosome, probably by its nucleosome remodeling activity^15,23^. Therefore, Rad26/CSB may have a coordinating function in the DNA repair process by recruiting the TC-NER factors and remodeling the nucleosome to restore RNAPII transcription. However, the mechanism by which Rad26/CSB binds and remodels the nucleosome has remained elusive.

In this work, we determined the cryo-electron microscopy (cryo-EM) structure of Rad26/CSB complexed with the nucleosome. We found that Rad26/CSB specifically binds near the entry/exit region at SHL ± 6. The ATPase domains, Lobe 1 and Lobe 2, of Rad26/CSB bind to the nucleosomal DNA in a reverse orientation, as compared to other nucleosome remodelers such as Snf2 and Ino80. The Rad26/CSB-specific KR loop and N-terminal disordered region are required for the nucleosome binding and remodeling.

## Results

### Cryo-EM structure of the Rad26/CSB-nucleosome complex

**Fig. 1:**
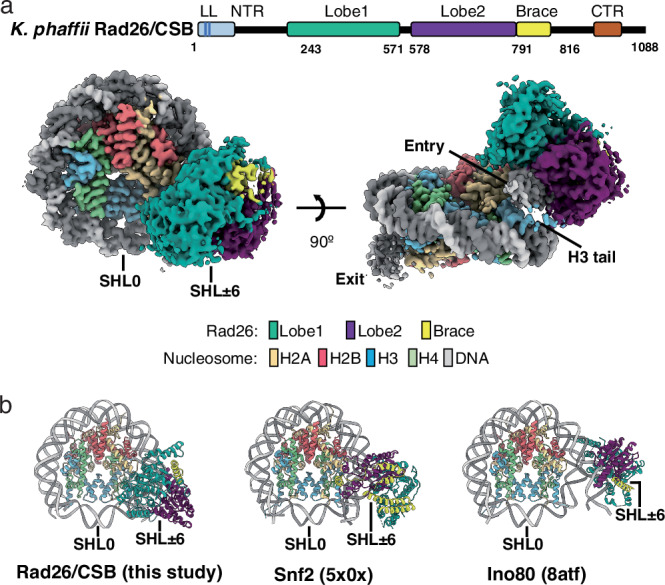
Cryo-EM structure of the *K. phaffii* Rad26/CSB-nucleosome complex. **a** Cryo-EM density map of the *K. phaffii* Rad26/CSB-nucleosome complex (EMDB: EMD-63262). Histones H2A, H2B, H3, and H4 are shown in ochre, red, light blue, and light green, respectively. DNA strands are colored light and dark gray. The color codes of the Rad26/CSB domains correspond to the schematic diagram shown at the top. Local sharpening using DeepEMhancer was applied to the cryo-EM density map. **b** Structure of the *K. phaffii* Rad26/CSB-nucleosome complex (left, PDB: 9LOX). Lobe 1, Lobe 2, and Brace domains are colored green, purple, and yellow, respectively. For comparison, structures of the Snf2-nucleosome complex (middle, PDB: 5X0X) and Ino80-nucleosome complex (right, PDB: 8ATF), in which their Lobe 1, Lobe 2, and Brace domains (colored green, purple, and yellow, respectively) are bound at the SHL ± 6 position of the nucleosome, are presented with the same nucleosome angle as the *K. phaffii* Rad26/CSB-nucleosome complex.

To study the mechanism by which Rad26/CSB remodels the nucleosome, we determined the cryo-EM structure of the Rad26/CSB-nucleosome complex (Fig. 1a and Supplementary Fig. 4). To do so, we reconstituted the nucleosome with *Komagataella phaffii* (formerly *Komagataella pastoris*) histones and a 169 base-pair DNA containing the Widom 601 sequence (Supplementary Fig. 2a). In this nucleosome, a 23 base-pair linker DNA protruded from one entry/exit region of the nucleosome. Purified *K. phaffii* Rad26 (a homologue of mammalian CSB) (Supplementary Fig. 2b) was then incubated with the reconstituted *K. phaffii* nucleosome in the presence of a non-hydrolyzable ATP analog, AMP-PNP (Supplementary Fig. 3a). *K. phaffii* Rad26 stably bound to the nucleosome, and the resulting complex was fractionated by sucrose gradient ultracentrifugation with a glutaraldehyde gradient (GraFix) (Supplementary Fig. 3b).

**Fig. 2:**
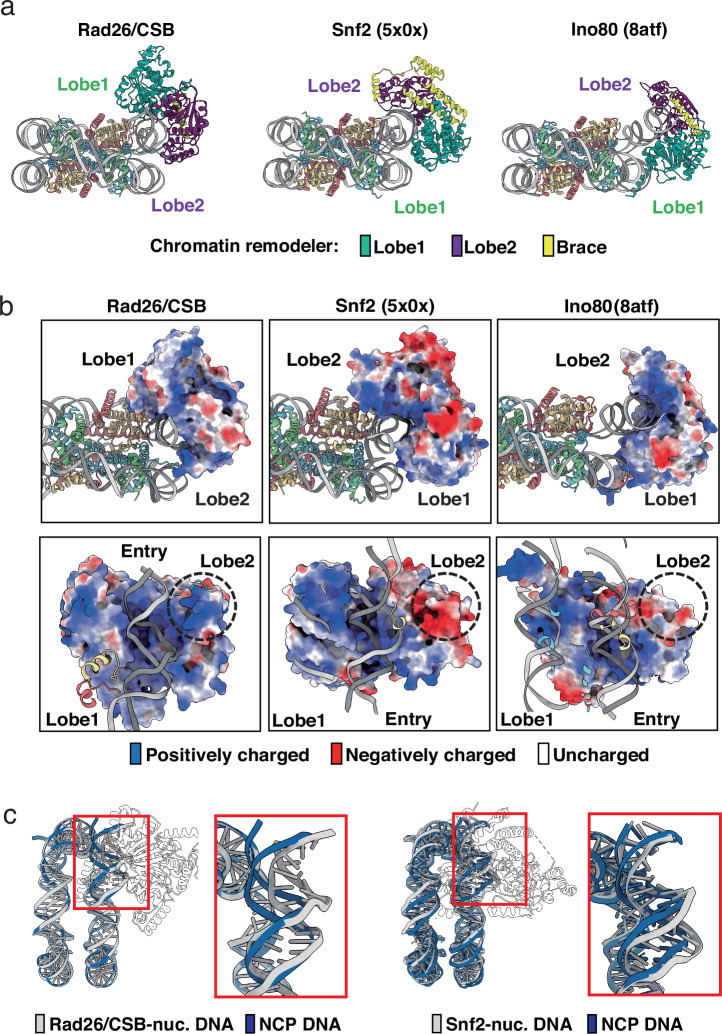
Distinct structural features of the *K. phaffii* Rad26/CSB-nucleosome complex. **a** Structural comparison of the *K. phaffii* Rad26/CSB-nucleosome complex with the Snf2-nucleosome and Ino80-nucleosome complexes, in which the Lobe 1 and Lobe 2 domains are bound at SHL ± 6 of the nucleosome. Color codes for the nucleosome, Rad26/CSB, Snf2, and Ino80 are the same as in Fig. 1. **b** Surface potential maps of Rad26/CSB (left), Snf2 (center), and Ino80 (right) bound to the nucleosome. Upper panels show side views of the complexes. Bottom panels show surface potential views of Rad26/CSB, Snf2, and Ino80 from the nucleosomal DNA side. **c** DNA path of the entry/exit region of the nucleosome bound to Rad26/CSB. The DNA in the Rad26/CSB-nucleosome complex is colored gray. The DNA in the free nucleosome is overlaid with blue. For comparison, a similar view of the Snf2-nucleosome complex is presented in the right panels.

We then conducted the cryo-EM single particle analysis and determined the structure of the Rad26/CSB-nucleosome complex (Fig. 1a). We also observed the low-resolution nucleosome structure with Rad26/CSB mapped symmetrically on both sides (Supplementary Fig. 4g). In the structure, the ATPase domains (Lobe 1 and Lobe 2) and Brace domain of Rad26/CSB were clearly visualized and bound to the DNA near the entry/exit region (SHL ± 6) (Fig. 1b). This SHL ± 6 binding mode is similar to that in previously reported structures of some chromatin remodelers, such as Snf2 and Ino80^24–27^ (Fig. 1b), but unlike the binding mode of other chromatin remodelers, Isw1 and Chd1, which reportedly bind to the SHL ± 2 position^24,25,28,29^. Intriguingly, Rad26/CSB exhibited a distinctive nucleosomal DNA binding feature: the orientation of the Lobe 1 and Lobe 2 domains relative to the nucleosomal DNA is reversed, as compared to that in the Snf2-nucleosome and Ino80-nucleosome complexes (Fig. 2a). The Lobe 2 domain of Rad26/CSB possesses a distinct basic patch that functions to bind the backbone phosphates of the nucleosomal DNA (Fig. 2b). Conversely, in Snf2 and Ino80, the corresponding basic patch is conserved in the Lobe 1 domain (Fig. 2b). This relocation of the basic patch specific to the Rad26/CSB Lobe 2 domain may underpin its unique mode of DNA binding in the nucleosome.

The nucleosomal DNA binding by Rad26/CSB induced DNA distortion and changed the DNA orientation around the SHL ± 6 position (Fig. 2c, left). The DNA structure around the SHL ± 6 position of the Rad26/CSB-nucleosome complex also differed from that of the Snf2-nucleosome complex (Fig. 2c, right). Therefore, the unique features of the nucleosome binding by Rad26/CSB may result in different DNA structures around the SHL ± 6 position of the nucleosome.

### The KR loop of the Rad26/CSB Lobe 2 domain

**Fig. 3:**
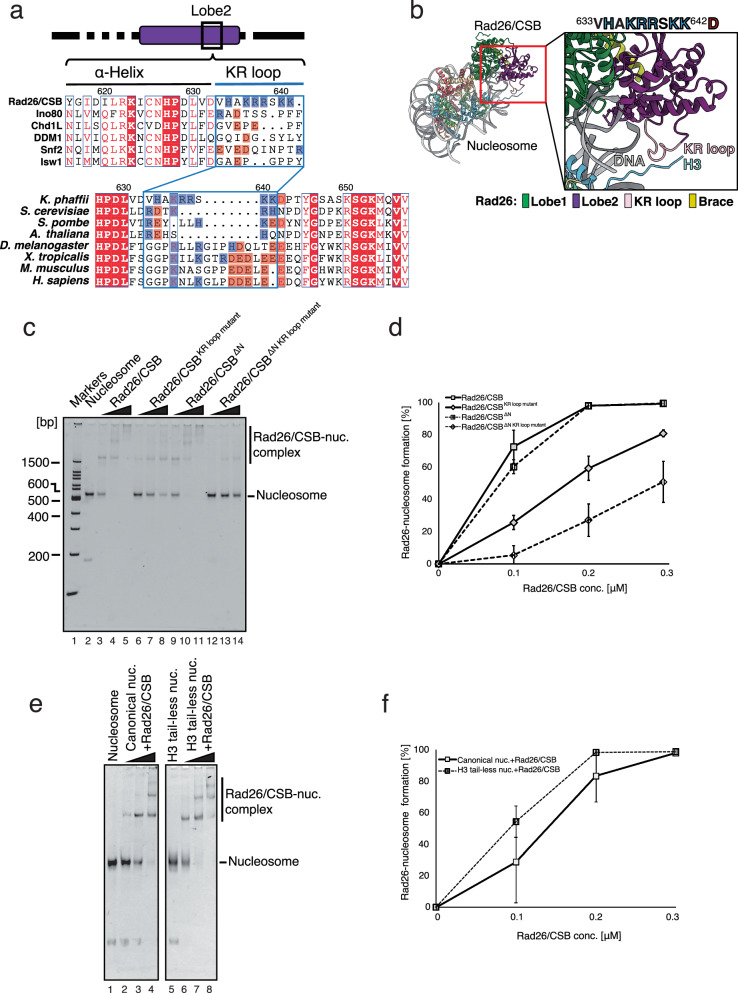
The KR loop of Rad26/CSB. **a** Comparison of the amino acid sequence of the *K. phaffii* Rad26/CSB Lobe 2 region with other chromatin remodelers, *Tetrahymena thermophila* Ino80, *Homo sapiens* ChdL, *Arabidopsis thaliana* DDM1, *Saccharomyces cerevisiae* Snf2, and *Saccharomyces cerevisiae* Isw1, whose structures have been reported^24,25,28,29^. White letters with a red background and red letters with a white background indicate amino acid residues conserved and mostly conserved among them. Letters with blue and orange backgrounds are basic and acidic amino acid residues, respectively, in the KR loop region. Amino acid sequence alignment of the KR loop regions of Rad26/CSB and Rad26/CSB homologs. **b** Structure of the *K. phaffii* Rad26/CSB-nucleosome complex. An enlarged view around the Rad26/CSB KR loop region is presented in the right panel. The model extends up to the Gly34 residue of H3, and the KR loop region corresponding to residues Val633 to Asp642 is shown. **c** The nucleosome binding assay. Rad26/CSB was incubated with the nucleosome. Lanes 1 and 2 indicate DNA markers and the nucleosome, respectively, Lanes 3–5, 6–8, 9–11, and 12–14 indicate the experiments with Rad26/CSB, Rad26/CSB KR loop mutant, Rad26/CSBΔN mutant (remodeling active), and Rad26/CSBΔN KR loop mutant, respectively. The protein concentrations of Rad26/CSB are 0.1, 0.2, and 0.3 μM. **d** Quantitation of Rad26/CSB-nucleosome complex formation. The complex formation ratios (%) were estimated relative to the band intensity of free nucleosome (lane 2). Average ratios of three independent experiments (Fig. 3c and Supplementary Fig. 13) were plotted with SD values. **e** Rad26/CSB binding to the H3 tail-less nucleosome. Lanes 1 and 5 indicate the canonical and H3 tail-less nucleosomes, respectively. Lanes 2–4 and 6–8 indicate the experiments with the canonical and H3 tail-less nucleosomes, respectively. The protein concentrations of Rad26/CSB are 0.1, 0.2, and 0.3 μM. **f** Quantitation of the Rad26/CSB-nucleosome complex formation. The complex formation ratios (%) were estimated relative to the band intensity of free nucleosomes (lanes 1 and 5). Average ratios of three independent experiments (Fig. 3e and Supplementary Fig. 9) were plotted with SD values. Source data are provided as a Source Data file.

The basic patch specific to the Rad26/CSB Lobe 2 domain contains the KR loop, which is composed of the amino acid residues 633-VHAKRRSKKD-642 (Fig. 3a and Supplementary Fig. 5a). The mainchain path of the KR loop is clearly traced in the Rad26/CSB-nucleosome complex, but the sidechain maps are not distinguishable (Supplementary Fig. 5b). The KR loop of Rad26/CSB is conserved from yeasts to humans, but is not present in the other nucleosome remodelers, Ino80, Chd1, DDM1, Snf2, and Isw1 (Fig. 3a). The Rad26/CSB KR loop is reportedly a major RNAPII binding surface that functions in the TC-NER pathway^15^. In the Rad26/CSB-nucleosome complex, the K636, R637, R638, K640, and K641 residues of the KR loop directly contact the DNA at the SHL ± 6 position (Fig. 3b). To investigate these interactions further, we prepared a Rad26/CSB KR loop mutant, in which ten consecutive residues (V633-D642) were replaced with glycine or serine to disrupt the KR loop (Fig. 3a).

**Fig. 4:**
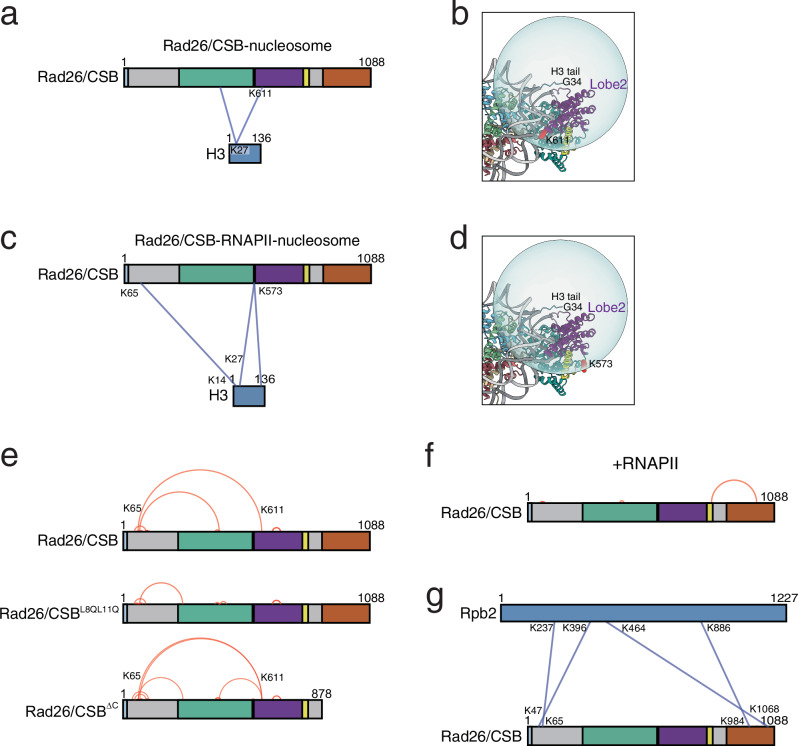
Crosslinking mass spectrometry of Rad26/CSB-nucleosome complex. **a** Intermolecular crosslinks between Rad26/CSB and histone H3 within the nucleosome were identified between lysine residues K611 of Rad26/CSB (lobe 2) and K27 of the H3 N-terminal tail. xQuest LD Scores are ≥ 30. **b** Localization of K611 in the structure of the Rad26/CSB-nucleosome complex. The blue sphere indicates a radius of 51.35 Å centered on H3 G34, representing the distance to H3 K27. **c** In the presence of RNAPII, intermolecular crosslinks were detected between K659 of Rad26/CSB (lobe 2) and K14 of the H3 N-terminal tail. xQuest LD Scores are ≥ 15. **d** Localization of K659 in the structure of the Rad26/CSB-nucleosome complex. The blue sphere indicates a radius of 51.35 Å centered on H3 G34, representing the distance to H3 K27. **e** Intramolecular crosslinks within Rad26/CSB, Rad26/CSB L8Q/L11Q, and Rad26/CSBΔC in the presence of AMP-PNP. The K65 residue near the auto-inhibitory domain and K611 in lobe 2 were crosslinked in Rad26/CSB and Rad26/CSBΔC, but not in Rad26/CSB L8Q/L11Q. xQuest LD Scores are ≥ 30. **f** Intramolecular crosslinks within Rad26/CSB in the presence of RNAPII. xQuest LD Scores are ≥ 18. **g** Intramolecular crosslinks between Rad26/CSB and Rpb2 in the presence of RNAPII. xQuest LD Scores are ≥ 18.

**Fig. 5:**
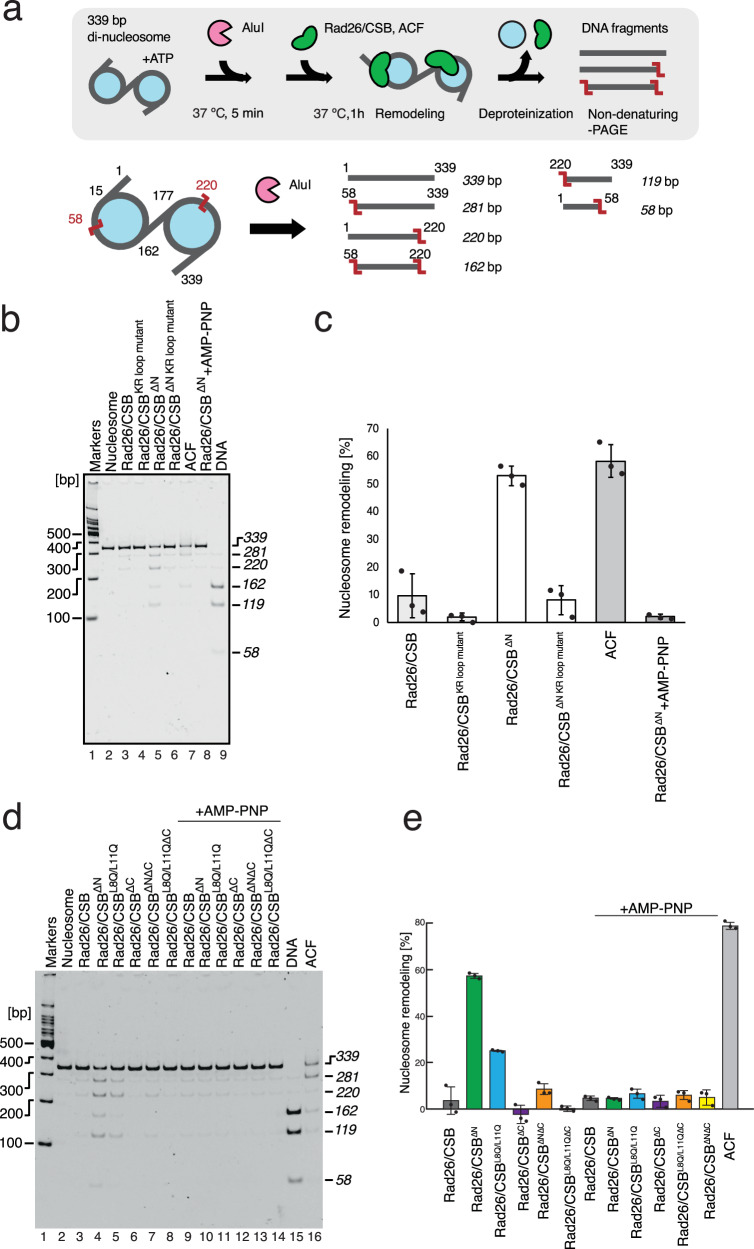
The nucleosome remodeling assay. **a** Schematic representation of the nucleosome remodeling assay. In the di-nucleosome substrate used in this assay, two *Alu*I restriction enzyme sites are concealed by nucleosome formation, but they become accessible when nucleosome remodeling occurs. Expected DNA fragments resulting from *Alu*I digestion are presented in the lower panel. **b** The nucleosome remodeling assay. The nucleosome remodeling reaction was conducted in the presence of Rad26/CSB (lane 3), Rad26/CSB KR loop mutant (lane 4), Rad26/CSBΔN (lane 5), Rad26/CSBΔN KR loop mutant (lane 6), and ACF (lane 7; a positive control). For a negative control, the reaction was conducted in the presence of Rad26/CSB and AMP-PMP (lane 8). Lanes 1 and 2 are DNA markers and the nucleosome substrate, respectively. Lane 9 presents DNA fragments resulting from *Alu*I digestion. Experiments were independently replicated three times, and these results are shown in Supplementary Fig. 11. **c** Quantitation of the nucleosome remodeling activity. The nucleosome remodeling ratios (%) were estimated relative to the band intensity of free nucleosomes (lane 2). Average ratios of three independent experiments (Fig. 5b and Supplementary Fig. 15) were plotted with SD values. **d** The nucleosome remodeling reaction was conducted in the presence of Rad26/CSB (lane 3), Rad26/CSB ∆N (lane 4), Rad26/CSB L8Q/L11Q (lane 5), Rad26/CSBΔC (lane 6), Rad26/CSBΔNΔC (lane 7), Rad26/CSB L8Q/L11Q ΔC (lane 8), and ACF (lane 16; a positive control). For a negative control, the reaction was conducted in the presence of Rad26/CSB or its mutants with AMP-PMP (lanes 9–14). Lanes 1 and 2 are DNA markers and the nucleosome substrate, respectively. Lane 9 presents DNA fragments resulting from *Alu*I digestion. Experiments were independently replicated three times, and these results are shown in Supplementary Fig. 16. **e** Quantitation of the nucleosome remodeling activity. The nucleosome remodeling ratios (%) were estimated relative to the band intensity of free nucleosomes (lane 2). Average ratios of three independent experiments (Fig. 5d and Supplementary Fig. 16) were plotted with SD values. Source data are provided as a Source Data file.

An electrophoretic mobility shift assay revealed that the Rad26/CSB KR loop mutant was clearly defective in nucleosome binding (Fig. 3c, d and Supplementary Fig. 12). This suggests that the KR loop-DNA interaction in the nucleosome is impaired in the Rad26/CSB KR loop mutant. Our cryo-EM structure also demonstrated that, in the Rad26/CSB-nucleosome complex, the KR loop resides near the N-terminal tail of H3 (Fig. 3b). Our crosslinking mass spectrometry (XL-MS) analysis revealed that the Lys611 residue of Rad26/CSB near the KR loop crosslinks with the Lys27 residue of the H3 N-terminal tail (Fig. 4a). To assess the feasibility of the H3 K27-Rad26 K611 crosslink, we estimated the maximum span permitted by the crosslink in the Rad26/CSB-nucleosome structure. In our cryo-EM model, H3 G34 is the first residue resolved in the density, and therefore the position of H3 K27 cannot be directly assigned. We therefore used H3 G34 as a reference point and estimated the maximum possible distance from G34 to K27 based on the length of the intervening peptide segment. The resulting distance of 51.35 Å corresponds to the sum of the estimated maximum length of the H3 K27–G34 segment, the spacer arm length of disuccinimidyl suberate (DSS), and the side-chain lengths of two lysine residues, and thus represents the theoretical upper limit that can be spanned by this crosslink. Based on this criterion, H3 K27 and Rad26 K611 are positioned within a distance compatible with crosslink formation in the cryo-EM structure (Fig. 4b, d). Therefore, the H3 N-terminal tail is located near the Rad26/CSB KR loop in the Rad26/CSB-nucleosome complex. To assess the interaction between the Rad26/CSB KR loop and the N-terminal tail of H3, we prepared the *K. phaffii* nucleosome lacking the N-terminal 33 residues of H3 (H3 tail-less) (Supplementary Fig. 8). Unexpectedly, Rad26/CSB exhibited somewhat higher affinity to the H3 tail-less nucleosome than the wild-type nucleosome (Fig. 3e, f and Supplementary Fig. 13). Consistently, the deletion of the H3 N-terminal tail did not affect the Rad26/CSB-mediated nucleosome remodeling (described later, Fig. 5). These findings suggest that the KR loop-H3 tail interaction is not essential for Rad26/CSB-nucleosome binding and remodeling. This conclusion is in line with previous observations showing that the N-terminal tail of H4, but not that of H3, is required for nucleosome remodeling by *Schizosaccharomyces pombe* Rad26 (Rhp26)^22^. An alternative explanation is that removal of the H3 N-terminal tail may alleviate steric or electrostatic interference with the KR loop, thereby facilitating more direct access of the KR loop to nucleosomal DNA. Therefore, while the KR loop and the H3 N-terminal tail are spatially proximal, their interaction does not appear to be essential for nucleosome binding or remodeling, and the KR loop-DNA interaction likely plays the dominant functional role.

Interestingly, in the XL-MS analysis in the presence of RNAPII stalled on a nucleosomal DNA position, the interaction between Rad26/CSB Lys611 and H3 K27 was not visible, and instead, crosslinking between Rad26/CSB Lys659 and H3 K14 was detected (Fig. 4c). The Rad26/CSB Lys611 and Lys659 residues may be located on the path of the H3 N-terminal tail in the complex (Fig. 4b, d). Therefore, the H3 N-terminal tail may remain bound on the Rad26/CSB surface during the RNAPII progression.

### The nucleosome remodeling activity of Rad26/CSB

We next tested whether the Rad26/CSB KR loop functions in the nucleosome remodeling activity. The *Saccharomyces cerevisiae* and *S. pombe* Rad26 proteins reportedly contain an auto-inhibitory domain in their N-terminal regions^21–23^. In fact, the deletion of the N-termini of these Rad26 proteins robustly promoted the nucleosome remodeling activity. We then prepared the Rad26/CSBΔN protein, which may exhibit activated nucleosome remodeling activity due to lack of the auto-inhibitory domain composed of the N-terminal 17 amino acid residues (Supplementary Fig. 6b). Our cryo-EM analysis confirmed that Rad26/CSBΔN binds to the SHL ± 6 position of the nucleosome (Supplementary Fig. 7). The N-terminal deletion minimally affects the overall structure of the Rad26/CSB-nucleosome complex, although Rad26ΔN appears to engage the H2A-H2B surface more extensively than the full-length Rad26-nucleosome complex.

We then conducted a restriction enzyme accessibility assay, in which di-nucleosome remodeling is detected by *AluI* digestion (Fig. 5a and Supplementary Fig. 9). In this assay, the *AluI* sites are initially protected by nucleosome formation but become accessible to *AluI* cleavage upon nucleosome relocation (Fig. 5a). As expected, Rad26/CSBΔN exhibited substantial nucleosome remodeling activity, while full-length Rad26/CSB weakly promoted it (Fig. 5b and Supplementary Fig. 14). We then tested whether the KR loop mutation in Rad26/CSBΔN affects the nucleosome remodeling activity, and found that the Rad26/CSBΔN KR loop mutant was considerably defective (Fig. 5b, c and Supplementary Fig. 14). The Rad26/CSBΔN KR loop mutant was also defective in nucleosome binding (Fig. 3c and Supplementary Fig. 12). Therefore, the KR loop of Rad26/CSB plays an essential role in the nucleosome remodeling activity through its nucleosome binding.

Consistent with the efficient H3 tail-less nucleosome binding by Rad26/CSB, Rad26/CSBΔN was proficient in the remodeling of the H3 tail-less nucleosome (Supplementary Figs. 10 and 16). Therefore, the H3 N-terminal tail may not contribute to the nucleosome binding and remodeling activities of Rad26/CSB, although it could function in the Rad26/CSB translocation from RNAPII to the nucleosome.

It should be noted that, in the control experiment with the nucleosome remodeler ACF, one DNA fragment was dominantly detected, suggesting that the nucleosome sliding mediated by ACF may expose only one *Alu*I site and the other site was still concealed by the nucleosome^30,31^. Conversely, in the presence of Rad26/CSBΔN, five DNA fragments were detected by the *Alu*I treatment, suggesting that Rad26/CSBΔN drastically remodels the nucleosomes, probably by nucleosome disruption. This is consistent with previous reports, in which *S. cerevisiae* and *S. pombe* Rad26s promoted nucleosome disruption^22,23^.

### The N-terminal and C-terminal regions of Rad26/CSB

The *K. phaffii* Rad26/CSB Leu8 and Leu11 residues (corresponding to Leu7 and Leu10 of *S. pombe* Rhp26) reportedly function in the auto-inhibition of Rad26/CSB-mediated nucleosome remodeling^22^. To examine this further, we prepared the *K. phaffii* Rad26/CSB mutant (Rad26/CSB L8QL11Q), in which the Leu8 and Leu11 residues were replaced by glutamine residues (Supplementary Fig. 6c). Glutamine was chosen because its sidechain moiety has a comparable size to leucine but is hydrophilic. Consistent with previous studies, Rad26/CSB L8QL11Q displayed constitutive nucleosome remodeling activity (Fig. 5d, e), at about half the level of Rad26/CSBΔN (Fig. 5d, e). These results suggest that the N-terminal Leu8 and Leu11 residues of Rad26/CSB indeed function in the auto-inhibition of Rad26/CSB.

To gain mechanistic insight, we performed XL-MS using the Rad26/CSB L8QL11Q mutant. In wild-type Rad26/CSB, the N-terminal Lys65 residue was crosslinked to Lys611, whereas this crosslink was not observed in Rad26/CSB L8QL11Q (Fig. 4e). The Lys65 and Lys611 residues of Rad26/CSB are located within the N-terminal disordered region and the Lobe 2 domain, respectively. In the Rad26/CSB-nucleosome complex, the Rad26/CSB Lys611 residue exists near the nucleosomal DNA (Fig. 4b). Therefore, the N-terminal disordered region of Rad26/CSB may directly contact its Lobe 2 domain and promote its auto-inhibition by regulating the Rad26/CSB-nucleosome interaction during remodeling. Interestingly, the Rad26/CSB intra-molecular interaction between Lys65 and Lys611 was not predominantly observed in the presence of RNAPII (Fig. 4f), suggesting that RNAPII may have the potential to release the auto-inhibition by disrupting the interaction between the N-terminal disordered region and Lobe 2 of Rad26/CSB.

It should be noted that, in the presence of RNAPII and elongation factors, the Rpb2-Rad26/CSB KR loop interaction, which is previously reported in the absence of the nucleosome, was not detected in the presence of the nucleosome. The XL-MS interaction observed between Rpb2 and Rad26 was mapped to the flexible N-termina and C-terminal regions of Rad26 (Fig. 4g). Therefore, these interactions are not visible in the cryo-EM structure.

We next studied the contribution of the Rad26/CSB C-terminal region, which may stabilize the chromatin association^22^. To this end, we prepared Rad26/CSBΔC, which lacks the C-terminal residues 879–1088 (Supplementary Fig. 6c). Rad26/CSBΔC bound the nucleosome efficiently (Supplementary Figs. 11 and 17) and retained the auto-inhibition of the nucleosome remodeling activity (Fig. 5d and Supplementary Fig. 15). Rad26/CSBΔN + ΔC and Rad26/CSB L8QL11Q + ΔC, in which the C-terminal residues were deleted from the Rad26/CSBΔN and Rad26/CSB L8QL11Q mutants, respectively, were also inactive in nucleosome remodeling (Fig. 5d, e). These results indicate that the C-terminal region of Rad26/CSB is essential for its nucleosome-remodeling activity, independently of the N-terminal auto-inhibitory region.

Our XL-MS analysis revealed that the Lys65-Lys611 crosslink observed in the wild-type Rad26/CSB was preserved in the Rad26/CSBΔC-nucleosome complex (Fig. 4e). Therefore, the C-terminal region of *K. phaffii* Rad26/CSB may not participate in the N-terminal-Lobe 2 interaction that mediates the auto-inhibition of the nucleosome remodeling activity.

## Discussion

In the TC-NER pathway, Rad26/CSB is recruited to the RNAPII stalled at the damaged DNA site, where it promotes the assembly of downstream DNA repair factors such as Cockayne syndrome protein A (CSA), UV-stimulated scaffold protein A (UVSSA), and transcription factor IIH (TFIIH)^3,6,8,17–19^. This scaffold function of Rad26/CSB explains why mutations in the *Rad26/CSB* gene cause Cockayne syndrome, which is a rare disorder characterized by developmental abnormality and TC-NER deficiency^3,16,18,19^. Rad26/CSB possesses nucleosome remodeling activity, although the scaffold function of Rad26/CSB does not require it^21–23^. However, the nucleosome remodeling activity of Rad26/CSB is reportedly required for RNAPII passage through the nucleosome to complete the TC-NER pathway^21–23^. However, the mechanism by which Rad26/CSB binds and remodels the nucleosome has remained enigmatic.

In the present study, we determined the cryo-EM structure of the Rad26/CSB-nucleosome complex and performed XL-MS and biochemical analyses. We found that (i) Rad26/CSB preferentially binds to the SHL ± 6 position of the nucleosomal DNA and distorts the entry/exit region of the nucleosomal DNA, (ii) the ATPase Lobe 1 and Lobe 2 domains bind to the nucleosomal DNA in the reverse orientation as compared to the Snf2 and Ino80-nucleosome remodelers bound to the SHL ± 6 position, and (iii) the Rad26/CSB Lobe 2 domain contains a basic patch including the KR loop, which functions in nucleosome binding and remodeling. These unique characteristics of Rad26/CSB may contribute to its specific function in TC-NER.

**Fig. 6:**
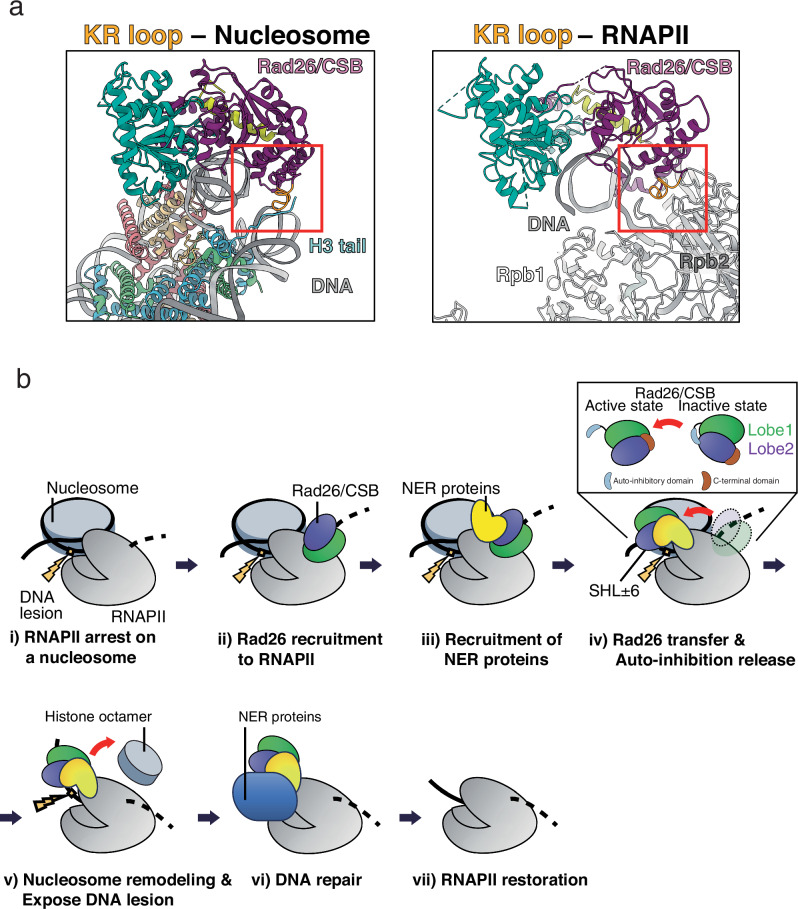
Structural comparison of the Rad26/CSB-nucleosome and Rad26/CSB-RNAPII complexes and a model for Rad26/CSB function in TC-NER. **a** Structural comparison of the Rad26/CSB-nucleosome complex (left panel) with the Rad26/CSB-RNAPII complex (right panel). The Rad26/CSB KR loop regions bound to the nucleosome or RNAPII are enlarged. **b** A model for the Rad26/CSB function during TC-NER.

The Rad26/CSB KR loop reportedly plays a crucial role in interacting with RNAPII^15^. Interestingly, we found that, in the Rad26/CSB-nucleosome complex, the Rad26/CSB KR loop also has a critical function in nucleosome binding and remodeling (Fig. 5). A structural comparison between the Rad26/CSB-nucleosome and Rad26/CSB-RNAPII complexes revealed that the nucleosome and RNAPII face a similar Rad26/CSB surface (Fig. 6a). Accordingly, the nucleosome binding and the RNAPII binding of Rad26/CSB may be mutually exclusive.

Based on our present findings, we propose a model for Rad26/CSB function in the TC-NER pathway within chromatin (Fig. 6b). When RNAPII is stalled at a DNA lesion, Rad26/CSB is recruited to the stalled complex. This recruitment facilitates the assembly of NER proteins, such as CSA, UVSSA, and TFIIH. It should be noted that CSA and UVSSA are specific to higher eukaryotes and are not conserved in yeast, whereas TFIIH is evolutionarily conserved from yeast to human and functions in both transcription and NER^1^. The assembly of NER proteins on the stalled RNAPII may also depend on its pausing position within the nucleosome, where the Rad26/CSB bound to RNAPII could be spatially positioned to allow the recruitment of repair proteins without steric hindrance from the nucleosome^15^. Subsequently, Rad26/CSB relocates from RNAPII to the nucleosome. Our XL-MS analyses showed that the Rad26/CSB KR loop contacts the N-terminal tail of the nucleosomal histone H3. Intriguingly, the interaction between Rad26/CSB and the H3 N-terminal tail becomes more evident in the presence of RNAPII, suggesting that RNAPII may actively support the Rad26/CSB relocation to the nucleosome. Given that the KR loop of Rad26/CSB is thought to initially interact with the stalled RNAPII, the mechanism by which Rad26/CSB relocates from RNAPII to the nucleosome during TC-NER may be the next important issue to resolve. This relocation may relieve the auto-inhibition mediated by the Rad26/CSB N-terminal region, activating its chromatin remodeling function. This is consistent with the XL-MS analysis, in which the intra-molecular interaction between the Rad26/CSB Lys65 and Lys611 residues may be released in the presence of RNAPII. The active Rad26/CSB then remodels the nucleosome to expose the DNA lesion site. After the DNA is repaired, RNAPII progression can resume.

With undamaged DNA, mammalian CSB reportedly functions as an ATP-dependent processivity factor that promotes RNAPII progression through nucleosomal barriers^23^. This suggests that Rad26/CSB may directly enhance RNAPII processivity through its interactions. In addition, our results indicate that Rad26/CSB may also act as a chromatin remodeler that could facilitate RNAPII progression on nucleosomal DNA during normal transcription elongation. Thus, in both transcription elongation and transcription-coupled repair, the chromatin-remodeling activity of Rad26/CSB likely represents a critical step that enables RNAPII to efficiently traverse chromatin.

Although direct evidence for the relocation of Rad26/CSB from RNAPII to the nucleosome has not yet been reported, several studies have provided indirect support for this hypothesis. Biochemical and structural data have shown that CSB can remodel nucleosomes in an ATP-dependent manner^21–23^, and that its association with RNAPII is dynamically regulated by CSA-UVSSA-TFIIH complexes and ubiquitin-dependent turnover mechanisms^5,32,33^. Live-cell imaging further indicated that CSB mobility changes upon DNA damage, suggesting dynamic transitions between the RNAPII-bound and chromatin-bound states. Together, these findings support a model in which Rad26/CSB initially acts as a scaffold on RNAPII to recruit repair factors, and is subsequently released to remodel the adjacent nucleosome and expose the lesion. Alternatively, another Rad26/CSB molecule, or other chromatin remodeler such as INO80, SWI/SNF, or RSC, could be recruited to perform this remodeling function, in coordination with the Rad26/CSB-mediated repair complex assembly. Thus, our structural model is consistent with and extends previous biochemical and cellular observations, providing a mechanistic framework for the functions of the Rad26/CSB bridge between transcription and chromatin remodeling during TC-NER.

## Methods

### Purification of *K. phaffii* histones and Rad26/CSB

*K. phaffii* histones H2A, H2B, H3.1, and H4 were purified as previously described^14^. Briefly, histones were produced as 6×His-tagged proteins in *E. coli* cells and purified under denaturing conditions, using Ni-NTA agarose resin (QIAGEN). The 6 × His tag-containing peptide region was removed via thrombin protease digestion. The proteins were then further purified by Mono S cation exchange chromatography (Cytiva), lyophilized, and stored as powders at 4 °C.

The DNA sequence of the *K. phaffii rad26/csb* gene was obtained from UniProt (C4R198_KOMPG) and cloned into the pGEX-6P-1 vector^15^, to produce full-length Rad26/CSB, the Rad26/CSB KR loop mutant (V633G/H634G/A635G/K636G/R637S/R638G/S639G/K640G/K641G/D642S), Rad26/CSB∆N (residues 18-1088), Rad26/CSB L8Q/L11Q, Rad26/CSB∆C (residues 1-878), Rad26/CSB L8Q/L11Q∆C, and Rad26/CSB∆N∆C (residues 18–878) with the PreScission protease recognition sequence to remove the 6 × His-tagged trigger factor (UniProt-ID P0A850). Purification of Rad26/CSB and its mutants was conducted by the method described previously^15^. Briefly, pGEX-6P-1-rad26/csb was introduced into *Escherichia coli* Rosetta2 (DE3) cells. The cells were harvested, and the pellet was resuspended in sonication buffer (20 mM Tris-HCl (pH 7.5), 500 mM NaCl, 5% glycerol, and 1 mM 2-mercaptoethanol) and stored at −80 °C. The pellet was thawed and then sonicated on ice (TAITEC: Output control 5, Duty cycle 40%, 60 s). The lysate was centrifuged, and the resulting supernatant was mixed with 4 ml of Ni-NTA agarose (Qiagen) and rotated at 4 °C for 1 h. The mixture was packed into an Econo-column (Bio-Rad), and the beads were washed with 200 ml of wash buffer A (20 mM Tris-HCl (pH 7.5), 500 mM NaCl, 5% glycerol, 10 mM imidazole, and 1 mM 2-mercaptoethanol). Rad26/CSB was then eluted by elution buffer A (20 mM Tris-HCl (pH 7.5), 500 mM NaCl, 5% glycerol, 200 mM imidazole, and 1 mM 2-mercaptoethanol). The eluate containing Rad26/CSB was mixed with Heparin-Sepharose resin and incubated at 4 °C for 1 h. The mixture was then washed with 150 ml of wash buffer B (20 mM HEPES-NaOH (pH 7.5), 200 mM NaCl, 5% glycerol, and 1 mM 2-mercaptoethanol). The His-tag portion was cleaved by the PreScission protease on the Heparin-Sepharose resin by an overnight incubation at 4 °C. The mixture was packed into an Econo-column (Bio-Rad), and the resin was washed with 50 column volumes of wash buffer B, and eluted with elution buffer B (20 mM HEPES-NaOH (pH 7.5), 1 M NaCl, 5% glycerol, and 1 mM 2-mercaptoethanol). The eluted Rad26/CSB was dialyzed against dialysis buffer A (20 mM HEPES-NaOH (pH 7.5), 500 mM NaCl, 5% glycerol, and 1 mM 2-mercaptoethanol) overnight at 4 °C. After dialysis, Rad26/CSB was subjected to HiLoad Superdex 16/60 S200 gel filtration chromatography (GE Healthcare), using dialysis buffer A. Fractions containing Rad26/CSB were collected and concentrated using an Amicon Ultra 50 kDa cutoff filter (Merck). Purified Rad26/CSB was stored at −80 °C.

### Preparation of DNAs

The 169 base-pair Widom 601 DNA fragment (Supplementary Table 1) was amplified by polymerase chain reaction (PCR) with the primers: 5´- CTGAGAATCCGGTGCCGAGGCC −3´ (sense) and 5´- ATCTATGAATTTCGCGACACAAGGCCTG −3´ (anti-sense). The PCR product was subjected to phenol-chloroform extraction and ethanol precipitation to concentrate the DNA. The plasmid containing the 339 base-pair DNA fragment derived from the modified Widom 601 sequence (Supplementary Table 1) was prepared as described previously^34,35^. Briefly, the plasmid was introduced into the *E. coli* (DH5α) strain and amplified. The *E. coli* cells were lysed by an alkaline method, and the 339 base-pair DNA fragment was prepared by digestion with restriction enzymes.

### Preparation of *K. phaffii* nucleosomes

The 169 base-pair or 339 base-pair DNA fragment was mixed with the *K. phaffii* histone octamer, which was reconstituted and purified by the method described previously^14^. For the H3 tail-less nucleosome, canonical H3 was replaced with tail-less H3 (residues 34–136). By using the salt dialysis method with reconstitution buffer 1 (10 mM Tris-HCl (pH 7.5), 2 M KCl, 1 mM EDTA, and 1 mM DTT) and reconstitution buffer 2 (10 mM Tris-HCl (pH 7.5), 250 mM KCl, 1 mM EDTA, and 1 mM DTT), the salt concentration was gradually reduced from 2 M to 250 mM, and the nucleosome was reconstituted. The reconstituted nucleosomes were fractionated by non-denaturing polyacrylamide gel electrophoresis, using a Prep Cell Model 491 apparatus (Bio-Rad). The concentrated nucleosomes were dialyzed against buffer containing 20 mM HEPES-NaOH (pH 7.5), 5% glycerol, and 1 mM DTT, and stored at −80 °C.

### Nucleosome binding assay

The *K. phaffii* nucleosome containing the 169 base-pair DNA (0.05 µM) was mixed with Rad26/CSB (0–0.3 µM) in 20 µL of binding buffer (20 mM HEPES-NaOH (pH 7.5), 50 mM NaCl, 1 mM MgCl_2_, 0.5% glycerol, 1 mM DTT, 0.1 mM 2-mercaptoethanol, and 1 mM AMP-PNP). The mixture was incubated at 30 °C for 30 min. Samples were mixed with sucrose to a final concentration of 5% (w/v), and then separated by 6% non-denaturing polyacrylamide gel electrophoresis (PAGE) containing 0.5× TBE (45 mM Tris-borate and 1 mM EDTA). After electrophoresis, the gel was stained with ethidium bromide to detect DNA, and the band intensity was quantitated using an Amersham Typhoon Imager (Cytiva).

### Preparation of the Rad26/CSB-nucleosome complex sample for cryo-EM

The *K. phaffii* nucleosome containing the 169 base-pair DNA (0.05 µM) was mixed with Rad26/CSB (0.2 µM) in 20 µL of binding buffer (20 mM HEPES-NaOH (pH 7.5), 50 mM NaCl, 1 mM MgCl_2_, 0.5% glycerol, 1 mM DTT, 0.1 mM 2-mercaptoethanol, and 1 mM AMP-PNP (Roche)), and was incubated at 30 °C for 30 min. The Rad26/CSB-nucleosome complex was fractionated and crosslinked by the GraFix method^36^ with low gradient buffer (10 mM HEPES-KOH (pH 7.5), 5% sucrose, 30 mM NaCl, 1 mM DTT, and 0.1 mM AMP-PNP) and high gradient buffer (10 mM HEPES-KOH (pH 7.5), 30% sucrose, 0.2% glutaraldehyde, 30 mM NaCl, 1 mM DTT, and 0.1 mM AMP-PNP). The sucrose and glutaraldehyde gradient solution was prepared using a Gradient Master 108 (BioComp Instruments). The Rad26/CSB-nucleosome complex was loaded on the top of the gradient and subjected to ultracentrifugation (Beckman Coulter) at 125,000 × *g* for 16 h at 4 °C, using an SW41Ti rotor. Fractions containing the Rad26/CSB-nucleosome complex were desalted with final buffer (10 mM Tris-HCl (pH 7.5), 30 mM NaCl, and 1 mM DTT) using a PD-10 column (Cytiva), and were concentrated using an Amicon Ultra-2 centrifugal filter unit (Merck). The Rad26ΔN-nucleosome complex was prepared using the same method as for the Rad26/CSB-nucleosome complex. A 2.5 µL aliquot of the Rad26/CSB-nucleosome complex (0.91 µM) was placed onto a Quantifoil R1.2/1.3 200-mesh Cu grid, which was pre-hydrated with a PIB-10 hydrophilization device (Vacuum Device, Japan). The Rad26/CSB-nucleosome complex was then blotted at 4 °C and 100% humidity for 6 s using a Vitrobot Mark IV (Thermo Fisher Scientific), and rapidly frozen in liquid ethane.

### Cryo-EM structural analysis of the Rad26/CSB -nucleosome complexes

Cryo-EM images of the Rad26/CSB-nucleosome complex and the Rad26/CSBΔN-nucleosome complex were obtained using a Krios G4 (Thermo Fisher Scientific) cryo-electron microscope, operated at 300 kV and equipped with a K3 direct electron detector and a BioQuantum energy filter (Gatan) with a 20 eV slit. Automated data collection was conducted using the EPU software (Thermo Fisher Scientific), with a pixel size of 1.06 Å, defocus values ranging from 1.0 to 2.5 μm, and doses of 59.7 electrons/Å² for the Rad26/CSB-nucleosome dataset, 61.4 electrons/Å² for dataset1 of the Rad26/CSBΔN-nucleosome, and 63.8 electrons/Å² for dataset2 of the Rad26/CSBΔN-nucleosome, across 40 frames. For the structural analysis of the Rad26/CSB-nucleosome complex and the Rad26/CSBΔN-nucleosome complex, 10,264 and 10,367 micrographs were acquired, respectively. All movie frames of the Rad26/CSB-nucleosome complex or the Rad26/CSBΔN-nucleosome complex were aligned using MOTIONCOR2^37^ with dose weighting, and the contrast transfer function (CTF) was estimated using CTFFIND4^38^. Image processing and structural analysis were performed with RELION 4.0-beta^39^.

In the cryo-EM analysis of the Rad26/CSB-nucleosome complex, a total of 4,747,760 particles were automatically picked from 10,264 micrographs, based on the 2D class averages generated with this dataset, and 3,425,355 particles were further selected by 2D classification. The initial 3D model was built based on the cryo-EM structure of the *K. phaffii* nucleosome (PDB: 7WLR). After 3D classification, 36,207 particles were selected for 3D refinement, followed by Bayesian polishing and CTF refinement. The resolution of the refined Rad26/CSB-nucleosome map was determined to be 3.5 Å, as estimated by the gold-standard Fourier Shell Correlation (FSC) at FSC = 0.143^40^. The final electron density map of the Rad26/CSB-nucleosome complex was refined with DeepEMhancer^41^. Additional parameters used for the cryo-EM structural analysis are listed in Supplementary Table 2. In the cryo-EM analysis of the Rad26/CSBΔN-nucleosome complex, 4,038,070 particles were picked from 10,367 micrographs, based on the 2D class averages generated with this dataset, and 621,299 particles were further selected by 2D classification. The initial 3D model was built based on the cryo-EM structure of the *K. phaffii* Rad26/CSB-nucleosome complex. After 3D classification, 226,294 particles were selected for 3D refinement, followed by Bayesian polishing and CTF refinement. The resolution of the refined Rad26/CSBΔN-nucleosome map was determined to be 4.63 Å, as estimated by the gold-standard Fourier Shell Correlation (FSC) at FSC = 0.143^40^.

### Model building and refinement of the Rad26/CSB-nucleosome

The atomic model of the Rad26/CSB-nucleosome was constructed using the cryo-EM structure of the *K. phaffii* nucleosome containing the Widom 601 DNA (PDB ID: 7WLR) as the template for the nucleosome region. The Rad26/CSB region was modeled based on the structure predicted by AlphaFold3 (ref. ^42^: https://golgi.sandbox.google.com). The atomic coordinates of the *K. phaffii* nucleosome and Rad26/CSB were fitted into the cryo-EM density map of the Rad26/CSB-nucleosome using UCSF ChimeraX^43^. Amino acid residues were manually added or removed using COOT^44^ or PyMOL (The PyMOL Molecular Graphics System, Version 2.0, Schrödinger, LLC). The constructed atomic model of the Rad26/CSB-nucleosome was refined using phenix.realspacerefine^45^, and the flexible fitting via molecular dynamics simulation was manually performed using ISOLDE^46^. The final model of the Rad26/CSB-nucleosome was evaluated with MolProbity^47^. Structural figures presented in the main text were prepared with ChimeraX^43^. Additional parameters used for the atomic model construction are listed in Supplementary Table 3.

### Nucleosome remodeling assay

The reaction mixture was prepared by mixing 2 μL of 0.5 μM *K. phaffii* di-nucleosome containing 339 base-pair DNA with 2 μL of 10× reaction buffer (62.5 μM MgCl_2_, 25 mM ATP or AMP-PNP, 12.5 mM DTT, 50 mM Tris-HCl (pH 7.5), and 10.0 mg/mL BSA). Subsequently, 2 µL of *Alu*I restriction enzyme (10 U/µL) was added. The total volume was adjusted to 18 μL with distilled deionized water, and the reaction mixture was incubated at 37 °C for 5 min. Rad26/CSB, the Rad26/CSB KR loop mutant, Rad26/CSB∆N, the Rad26/CSB∆N KR loop mutant, Rad26/CSB L8Q/L11Q, Rad26/CSB∆C, Rad26/CSB∆N∆C, or Rad26/CSB L8Q/L11Q∆C (each at 0.4 μM) was then added to the reaction mixture, and the chromatin remodeling reaction was carried out in a total volume of 20 μL. In the control experiments, Rad26/CSB was replaced with an equivalent amount of ACF (Active Motif; 0.05 μg/μL). The reaction mixture was incubated at 37 °C for 30 min, and terminated by adding stop buffer (20 mM Tris-HCl (pH 7.5), 80 mM EDTA (pH 8.0), 0.25% SDS, and 1 mg/mL Proteinase K). After an incubation at room temperature for 30 min, an equal volume of phenol/chloroform/isoamyl alcohol was added, and the mixture was vortexed vigorously for 1 min. The samples were centrifuged at 20,400 × *g* for 5 min at 4 °C, and the supernatants were analyzed by 6 or 10% non-denaturing PAGE in 0.5× TBE buffer (45 mM Tris-borate and 1 mM EDTA). After electrophoresis, the gel was stained with ethidium bromide, and DNA band intensities were quantified using an Amersham Typhoon Imager (Cytiva).

For the H3 tail-less nucleosome, the reaction mixture was prepared by mixing 2 μL of 0.5 μM *K. phaffii* nucleosomes, containing either canonical H3 or tail-less H3 (residues 34–136) with the 169 bp DNA, with 2 μL of 10× reaction buffer (62.5 μM MgCl_2_, 25 mM ATP or AMP-PNP, 12.5 mM DTT, 50 mM Tris-HCl (pH 7.5), and 10.0 mg/mL BSA). Subsequently, 2 µL of *Alu*I restriction enzyme (10 U/µL) was added. The total volume was adjusted to 18 μL with distilled deionized water, and the reaction mixture was incubated at 37 °C for 5 min. Rad26/CSB, the Rad26/CSB KR loop mutant, Rad26/CSB∆N, or Rad26/CSB∆N KR loop mutant (each at 0.4 μM) was then added to the reaction mixture, and the chromatin remodeling reaction was carried out in a total volume of 20 μL. In the control experiments, Rad26/CSB was replaced with an equivalent amount of ACF (Active Motif; 0.05 μg/μL). The reaction mixture was incubated at 37 °C for 30 min, and terminated by adding stop buffer (20 mM Tris-HCl (pH 7.5), 80 mM EDTA (pH 8.0), 0.25% SDS, and 1 mg/mL Proteinase K). After an incubation at room temperature for 30 min, an equal volume of phenol/chloroform/isoamyl alcohol was added, and the mixture was vortexed vigorously for 1 min. The samples were centrifuged at 20,400 × *g* for 5 min at 4 °C, and the supernatants were analyzed by 10% non-denaturing PAGE in 0.5× TBE buffer (45 mM Tris-borate and 1 mM EDTA). After electrophoresis, the gel was stained with ethidium bromide, and DNA band intensities were quantified using an Amersham Typhoon Imager (Cytiva).

### Crosslinking mass spectrometry

Crosslinking mass spectrometry was performed as described previously^48,49^. Briefly, for the Rad26/CSB-nucleosome complex, the nucleosome (0.5 µM) was mixed with Rad26/CSB (4 µM) in a 1:8 molar ratio. The sample was then crosslinked with 0.8 mM DSS-H12/D12 (Creative Molecules) at 30 °C for 30 min. The reaction was stopped by adding 53 mM Tris-HCl (pH 7.5). For the Rad26/CSB-RNAPII-nucleosome complex, RNAPII, TFIIS, Spt4/5, Elf1, and *K. phaffii* nucleosomes were purified, and the transcription reaction was performed as previously described^12–14,50,51^. Briefly, the transcription reaction was assembled by combining the *K. phaffii* nucleosome (1.5 µM), Rad26/CSB (1.5 µM), RNAPII (1.5 µM), DY647-labeled RNA primer (2 µM), TFIIS (1.5 µM), Spt4/5 (6 µM), and Elf1 (15 µM) in buffer, containing 20 mM HEPES-KOH (pH 7.5), 50 mM potassium acetate, 5 mM magnesium chloride, 0.2 µM zinc acetate, and 0.4 mM each of ATP, UTP, and GTP. The reaction mixture was incubated at 30 °C for 30 min, and then crosslinked with 0.8 mM DSS-H12/D12 (Creative Molecules) under the same conditions. The reaction was stopped by adding 53 mM Tris-HCl (pH 7.5). Both crosslinked samples were reduced, alkylated, and subsequently digested using sequencing-grade Trypsin/Lys-C Mix (Promega) at an enzyme-to-substrate ratio of 1:50 (w/w). The resulting peptides were fractionated on a Superdex 30 Increase 3.2/300 column (GE Healthcare) using a mobile phase composed of 25% acetonitrile and 0.1% trifluoroacetic acid (TFA), yielding 30–40 fractions. Fractions (100 µL each) were collected, dried, and reconstituted in 0.1% TFA. For each condition, one biological sample was analyzed in two technical replicate LC-MS/MS measurements (*n* = 1). Liquid chromatography-tandem mass spectrometry (LC-MS/MS) analysis was performed using an Orbitrap Fusion mass spectrometer coupled to an Ultimate 3000 nano-LC system (Thermo Fisher Scientific). Crosslinked peptides were identified using the xQuest/xProphet pipeline, and crosslink maps were visualized through the xVis web server^52^.

Crosslink MS analysis was performed according to the settings described in a previous study using xQuest/xProphet version 2.1.5^48^. MS data were acquired using an Orbitrap mass spectrometer at a resolution of 60,000. MS/MS analysis was performed by ion selection with a quadrupole, followed by fragmentation by collision-induced dissociation (CID) and detection in an ion trap. Data processing parameters were defined in the xmm.def file for isotopically labeled samples. The parameters were set as follows: Isopair_Mr_tolerance = 15, Isopair_Tr_tolerance = 3.0, Isotopeshift = 12.075321, and Printisotopicscanpairs = 1. For analyses of samples containing nucleosomes and Rad26 (Fig. 4a and e), a FASTA file comprising n histones (H2A, H2B, H3.1, and H4) and Rad26 (WT, L8Q/L11Q, and ΔC) was used. For analyses of samples containing nucleosomes, Rad26, and RNA polymerase II (Fig.4c, f, and g), a FASTA file containing histone H3, Rad26 (WT), RNA polymerase II subunits (Rpb2 and Rpb4), and transcription factors (Spt4, Spt5, Elf1, and TFIIS) was used. Approximately 1500–3300 crosslinked peptides were identified per analysis. To discuss crosslinked peptides identified with high confidence and a low likelihood of false positives, only the top several dozen crosslinks with the highest LD scores were selected for further analysis (Fig. 4). In Fig. 4a and e, all crosslinks with LD scores ≥30 are shown, whereas in Fig. 4c, f, and g, all crosslinks with LD scores ≥18 are shown. All raw data, including mass spectrometry files, databases, and identification results, have been deposited in the ProteomeXchange Consortium via the JPOST repository (PXD070143)^53^.

### Reporting summary

Further information on research design is available in the Nature Portfolio Reporting Summary linked to this article.

## Supplementary information

Supplementary Information

Reporting Summary

Transparent Peer Review file

## Source data

Source Data

## Data Availability

The cryo-EM maps and atomic coordinates of the *K. phaffii* Rad26/CSB-nucleosome complex have been deposited in the Electron Microscopy Data Bank (EMDB) and the Protein Data Bank (PDB), respectively. The cryo-EM maps of the *K. phaffii* Rad26/CSBΔN-nucleosome complex have been deposited in the EMDB. The accession codes for these cryo-EM maps are EMD-63262 (*K. phaffii* Rad26-nucleosome) [https://www.ebi.ac.uk/emdb/EMD-63262] and EMD-63507 (*K. phaffii* Rad26/CSBΔN-nucleosome) [https://www.ebi.ac.uk/emdb/EMD-63507]. The accession code for the atomic coordinates is 9LOX (*K. phaffii* Rad26-nucleosome) [10.2210/pdb9LOX/pdb]. Uncropped images are provided in Supplementary Fig. 19. Figures were created with Chimera, ChimeraX and Adobe Illustrator. Raw mass spectrometry data have been deposited in the ProteomeXchange Consortium via the JPOST repository (PXD070143) [http://proteomecentral.proteomexchange.org/cgi/GetDataset?ID = PX070143]^53^. Source data are provided with this paper.
