# Supplementary material for: Structural basis of nucleosome remodeling by Cockayne syndrome B homologue Komagataella phaffii Rad26
Source: Nat Commun. 2026 Jun 24;17:4881. doi: 10.1038/s41467-026-73500-7 (PMC13294370; doi:10.1038/s41467-026-73500-7)
Supplement: Supplementary file 1 — Supplementary Information [file 41467_2026_73500_MOESM1_ESM.pdf]

Supplementary Information for

Structural basis of nucleosome remodeling by Cockayne syndrome B homologue *Komagataella phaffii* Rad26

Yutaro Fukushima[1,2†], Chiaki Kinoshita[1,3], Lumi Negishi[1], Tomoya Kujirai[1,4], Yuki Kobayashi[1], Mitsuo Ogasawara[1], Haruhiko Ehara[4], Shun-ichi Sekine[4], Wataru Kagawa[3], Hitoshi Kurumizaka[1,2,4,5]*, Yoshimasa Takizawa[1,6†]*

[1]Laboratory of Chromatin Structure and Function, Institute for Quantitative Biosciences, The University of Tokyo, 1-1-1 Yayoi, Bunkyo-ku, Tokyo 113-0032, Japan.
[2]Department of Biological Sciences, Graduate School of Science, The University of Tokyo, 1-1-1 Yayoi, Bunkyo-ku, Tokyo 113-0032, Japan.
[3]Department of Chemistry, Graduate School of Science and Engineering, Meisei University, 2-1-1 Hodokubo, Hino-shi, Tokyo 191-8506, Japan.
[4]RIKEN Center for Integrative Medical Sciences, 1-7-22 Suehiro-cho, Tsurumi-ku, Yokohama, 230-0045, Japan
[5]Division of Chromatin Structure and Function, Department of Multidisciplinary Life Science, Medical Institute of Bioregulation, Kyushu University, 3-1-1 Maidashi, Higashi-ku, Fukuoka 812-0054, Japan
[6]Department of Computational Biology and Medical Sciences, Graduate School of Frontier Sciences, The University of Tokyo, 1-1-1 Yayoi, Bunkyo-ku, Tokyo 113-0032, Japan.

†These authors contributed equally to this work.

*Corresponding author’s e-mail address: ytakizawa@iqb.u-tokyo.ac.jp or kurumizaka@iqb.u-tokyo.ac.jp

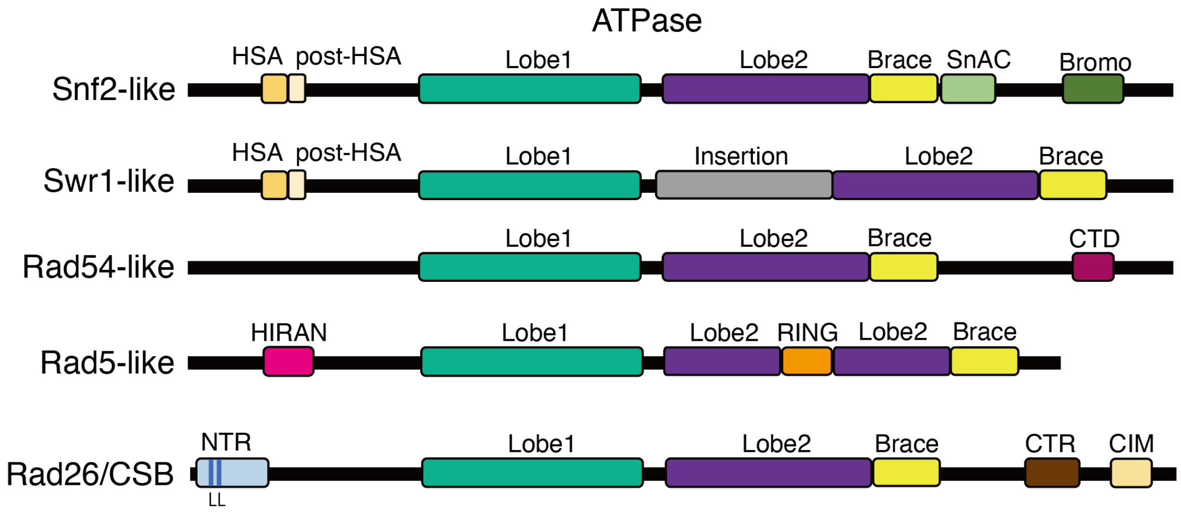

Supplementary Fig. 1. Schematic diagram of conserved domains of chromatin remodelers. Each chromatin remodeler contains a conserved catalytic domain known as the ATPase domain, composed of Lobe 1 and Lobe 2, along with a Brace domain. Rad26/CSB contains the NTR (N-terminal region) including an auto-inhibition domain with a leucine latch motif (L**L) essential for chromatin remodeling, CTR (C-terminal region) and a CIM (CSA-interaction motif).

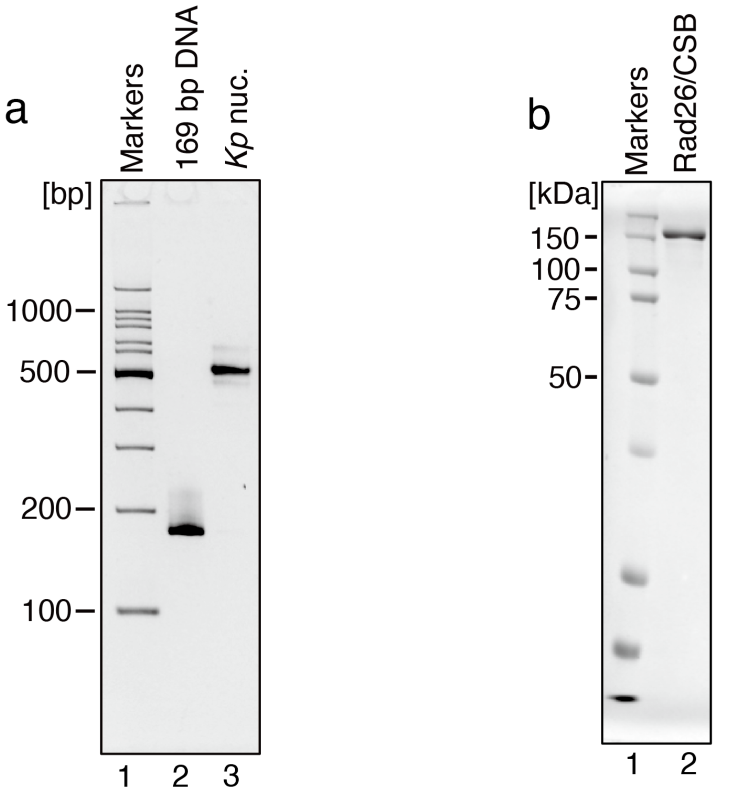

Supplementary Fig. 2. Preparation of Rad26/CSB and *K. phaffii* nucleosome.
(a) Non-denaturing-PAGE analysis of the purified *K. phaffii* nucleosome containing the 169 base-pair DNA with ethidium bromide staining. The uncropped gel image is provided in Supplementary Fig. 19. (b) SDS-PAGE analysis of the purified Rad26/CSB with Coomassie Brilliant Blue staining. The uncropped gel image is provided in Supplementary Fig. 19.

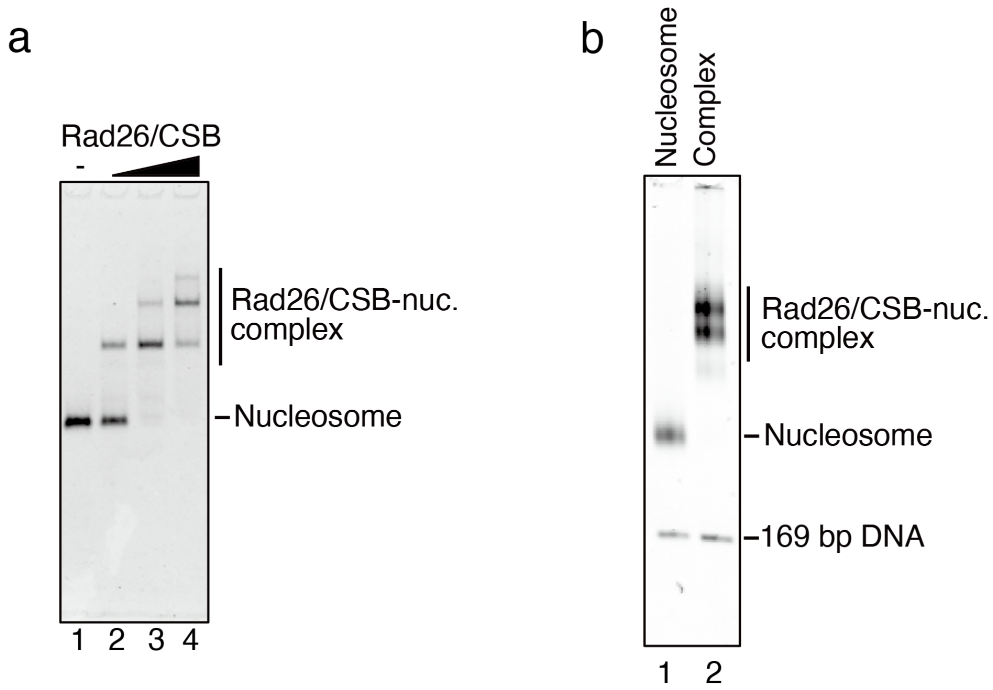

Supplementary Fig. 3. Preparation of the Rad26/CSB-nucleosome complex for cryo-EM.
(a) Electrophoretic mobility shift assay of Rad26/CSB bound to the nucleosome. The nucleosome (0.05 μM) was mixed with Rad26/CSB and analyzed by non-denaturing PAGE with ethidium bromide staining. The protein concentrations of Rad26/CSB are 0.1, 0.2, and 0.3 μM. The uncropped gel image is provided in Supplementary Fig. 19. (b) Non-denaturing PAGE analysis of the Rad26/CSB-nucleosome complex after GraFix, with ethidium bromide staining. The uncropped gel image is provided in Supplementary Fig. 19.

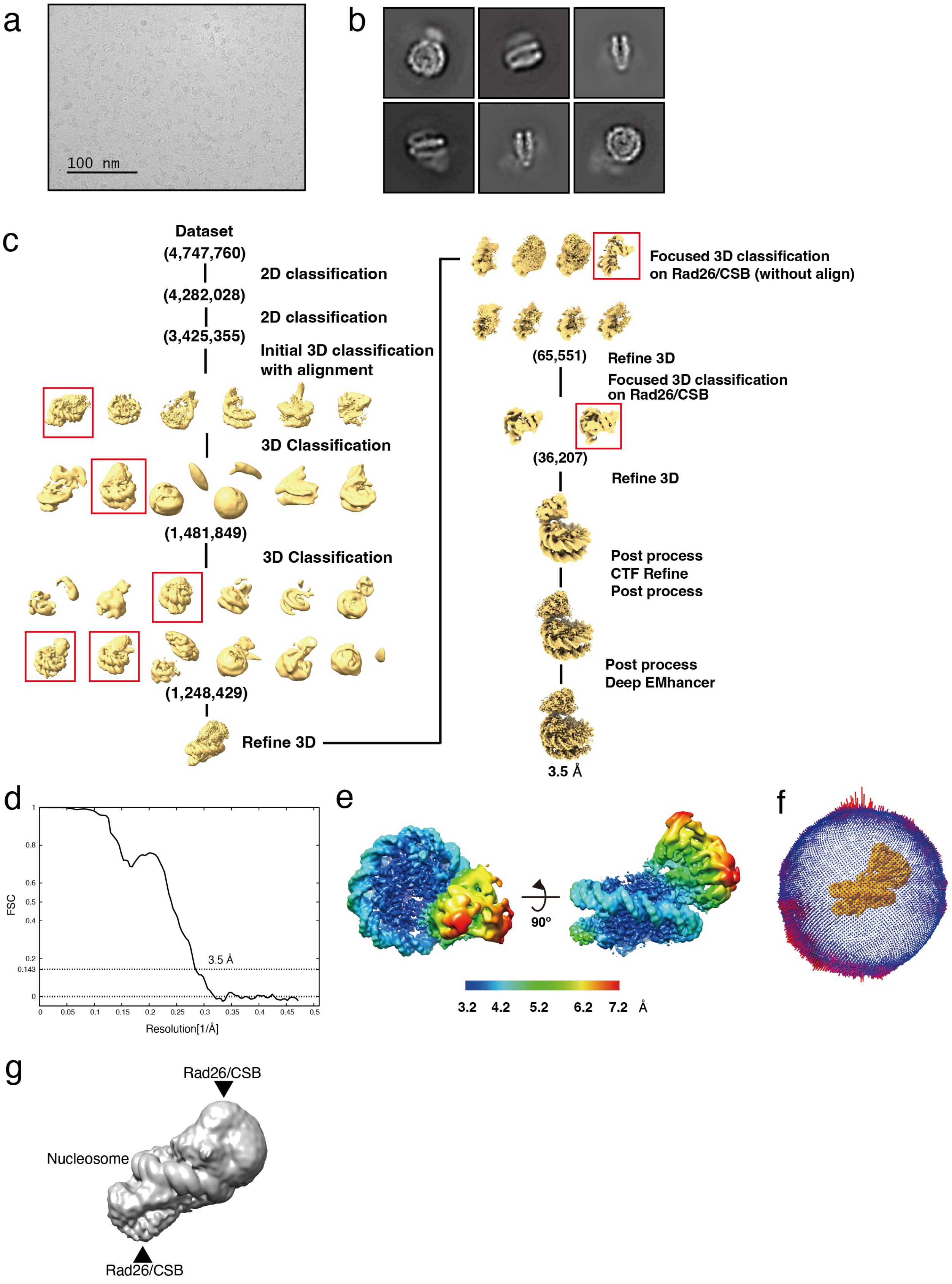
a
100 nm
b
c
Dataset
(4,747,760)
2D classification
(4,282,028)
2D classification
(3,425,355)
Initial 3D classification
with alignment
3D Classification
(1,481,849)
3D Classification
(1,248,429)
Refine 3D
Focused 3D classification
on Rad26/CSB (without align)
(65,551)
Refine 3D
Focused 3D classification
on Rad26/CSB
(36,207)
Refine 3D
Post process
CTF Refine
Post process
Post process
Deep EMhancer
3.5 Å
d
FSC
3.5 Å
0.143
Resolution[1/Å]
e
90°
3.2 4.2 5.2 6.2 7.2 Å
f
g
Rad26/CSB
Nucleosome
Rad26/CSB

Supplementary Fig. 4. Cryo-EM data collection and image processing of the Rad26/CSB-nucleosome complex.

(a) Representative cryo-EM micrograph of the Rad26/CSB-nucleosome complex. Scale bar indicates 100 nm. (b) Representative 2D class averages of the Rad26-nucleosome complex. Box size is 24 nm. (c) Flow chart of the image processing of the Rad26/CSB-nucleosome complex. (d) Fourier Shell Correlation (FSC) curve of the Rad26/CSB-nucleosome complex. (e) Local resolution map of the Rad26/CSB-nucleosome complex. (f) Euler angular distribution map of the Rad26/CSB-nucleosome complex. (g) Representative 3D class of the Rad26/CSB–nucleosome complex showing two Rad26/CSB molecules bound symmetrically on the nucleosome.

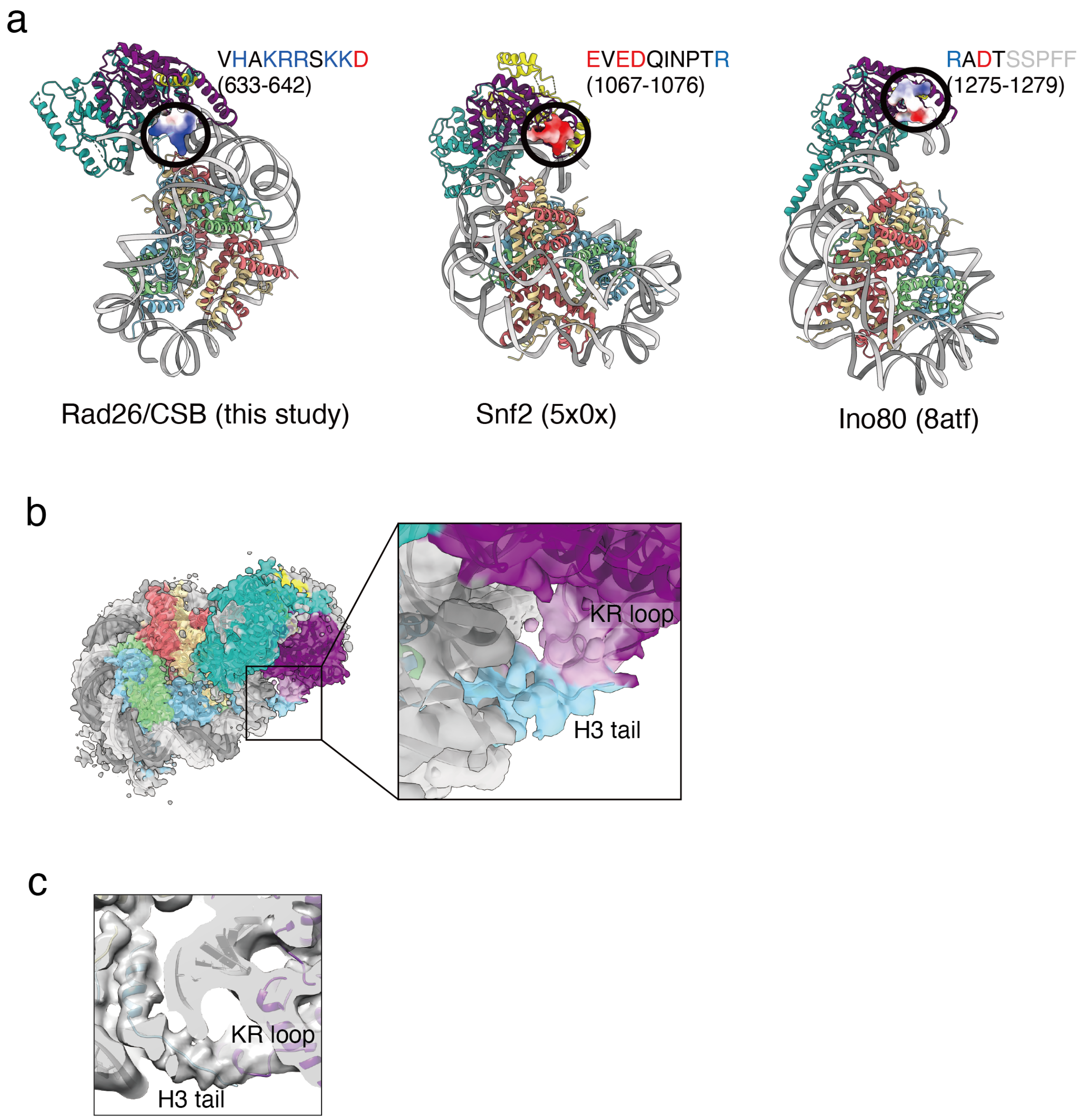

Supplementary Fig. 5. Comparison of the KR loop region of the Rad26/CSB-nucleosome complex with other nucleosome remodeler structures.

(a) Comparison of electrostatic potentials of loop regions contained within Lobe 2 in the molecular models of *K. phaffii* Rad26/CSB, Snf2 (PDB ID: 5X0X), and Ino80 (PDB ID: 8ATF). Electrostatic potential is displayed in blue (positive charge) and red (negative charge). (b) Docking of the cryo-EM map and model of the Rad26/CSB-nucleosome complex. Close-up view of the H3 tail and KR loop region. (c) Docking of model into the cryo-EM map low-pass filtered to 6 Å, shown as a close-up view of the H3 tail region in the Rad26/CSB–nucleosome complex.

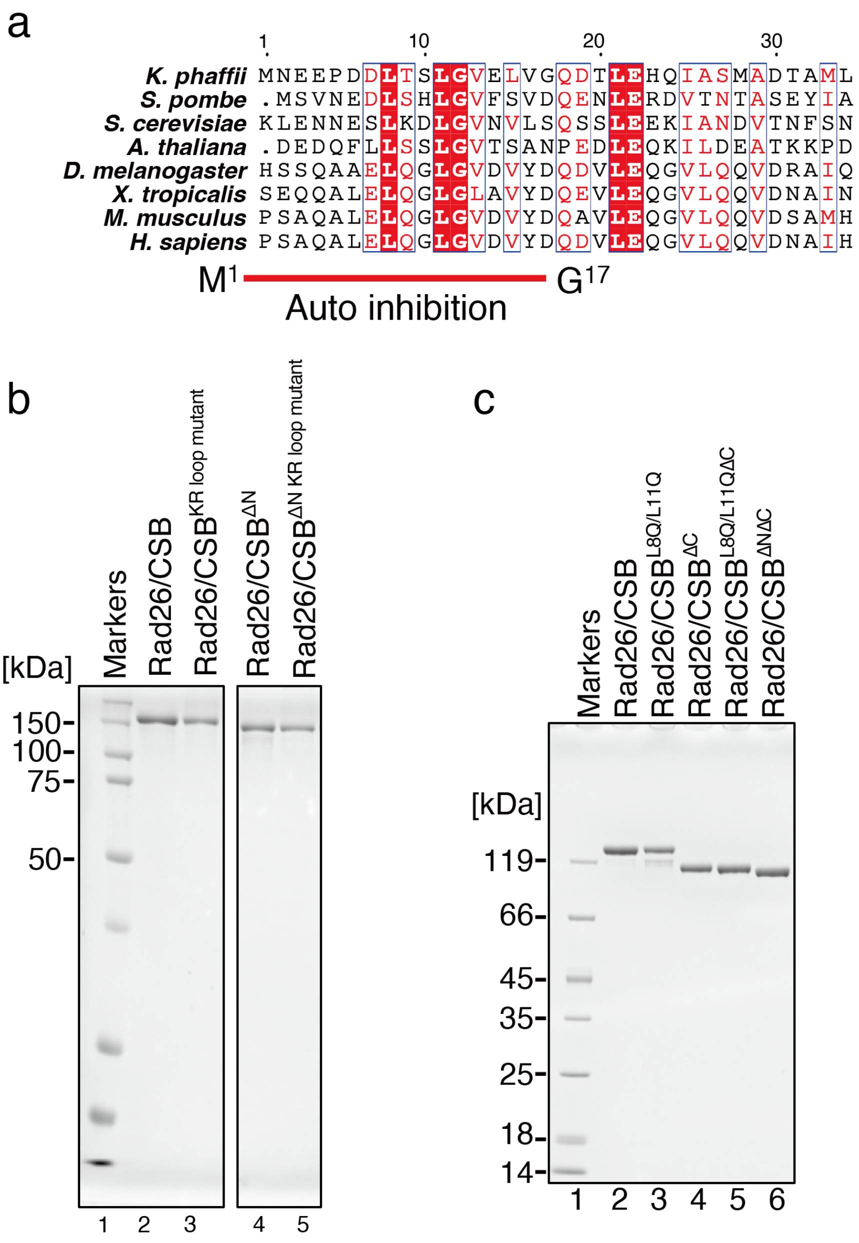

Supplementary Fig. 6. Preparation of the Rad26/CSB mutants.

(a) Amino acid sequence alignment of the N-terminal region of Rad26/CSB and its homologues. (b) SDS-PAGE analysis of purified Rad26/CSB and its mutants, Rad26/CSB, Rad26/CSB KR loop mutant, Rad26/CSBΔN, and Rad26/CSBΔN KR loop mutant, with Coomassie Brilliant Blue staining. The uncropped gel image is provided in Supplementary Fig. 19. (c) SDS-PAGE analysis of purified Rad26/CSB, Rad26/CSB L8Q/L11Q, Rad26/CSBΔC, Rad26/CSB L8Q/L11QΔC, and Rad26/CSBΔNΔC, with Coomassie Brilliant Blue staining. The uncropped gel image is provided in Supplementary Fig. 19.

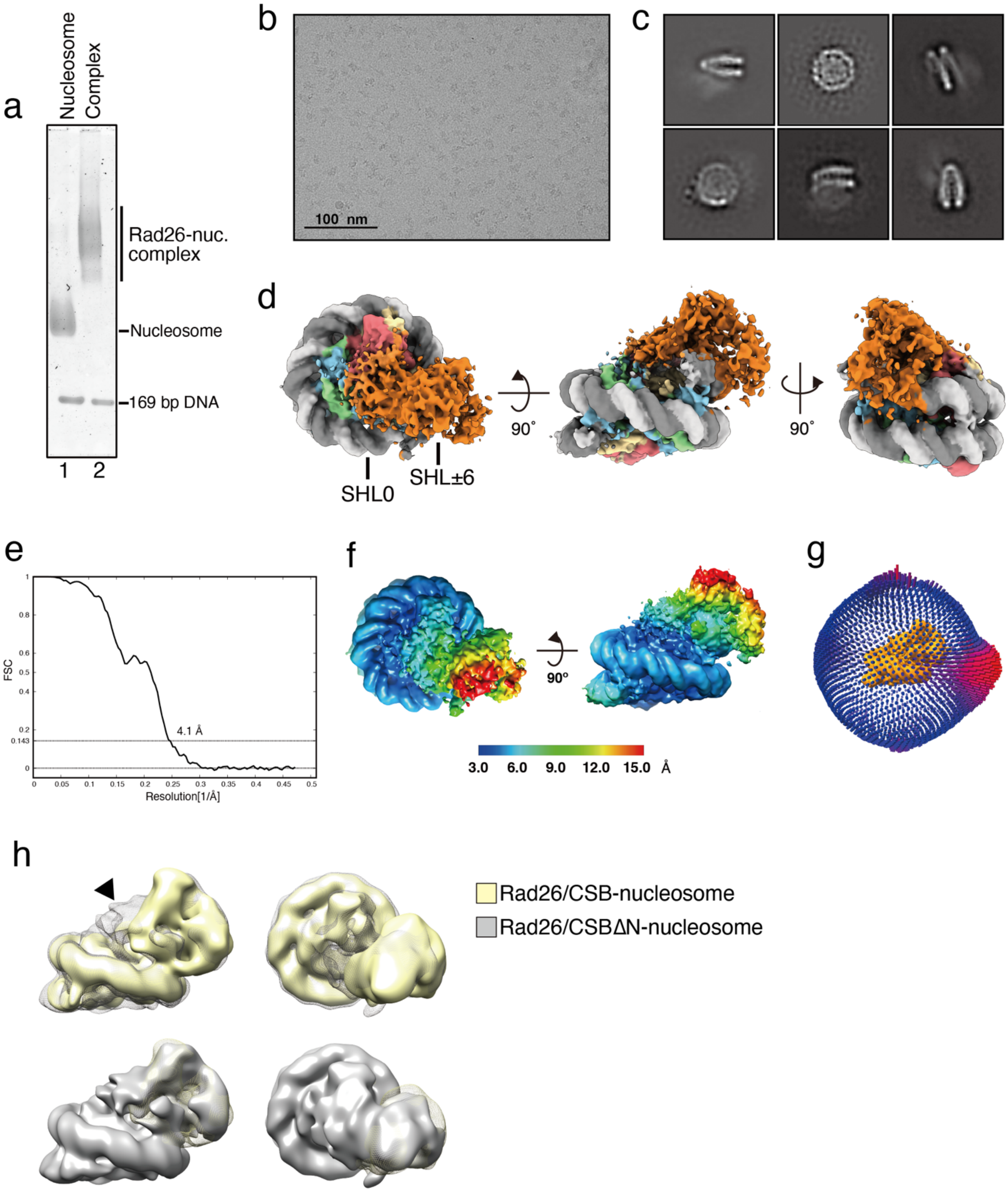

Supplementary Fig. 7. Cryo-EM data collection and image processing of the Rad26/CSBΔN-nucleosome complex.

(a) Non-denaturing PAGE analysis of the Rad26/CSBΔN-nucleosome complex after GraFix, with ethidium bromide staining. The uncropped gel image is provided in Supplementary Fig. 13. (b)

Representative cryo-EM micrograph of the Rad26/CSBΔN-nucleosome complex. Scale bar indicates 100 nm. (c) Representative 2D class averages of the Rad26/CSBΔN-nucleosome complex. Box size is 24 nm. (d) Cryo-EM map of the Rad26/CSBΔN-nucleosome complex. Histones H2A, H2B, H3, and H4 are shown in ochre, red, light blue, and green, respectively. DNA and Rad26/CSB are shown in gray and orange, respectively. (e) Fourier Shell Correlation (FSC) curve of the Rad26/CSBΔN-nucleosome complex. (f) Local resolution map of the Rad26/CSBΔN-nucleosome complex. (g) Euler angular distribution map of the Rad26/CSBΔN-nucleosome complex. (h) Comparison of cryo-EM maps between the Rad26/CSB–nucleosome complex (yellow) and the Rad26/CSBΔN–nucleosome complex (gray), both low-pass filtered to 15 Å resolution. Black triangles indicate additional density in the Rad26/CSBΔN-nucleosome complex compared with the Rad26/CSB-nucleosome complex.

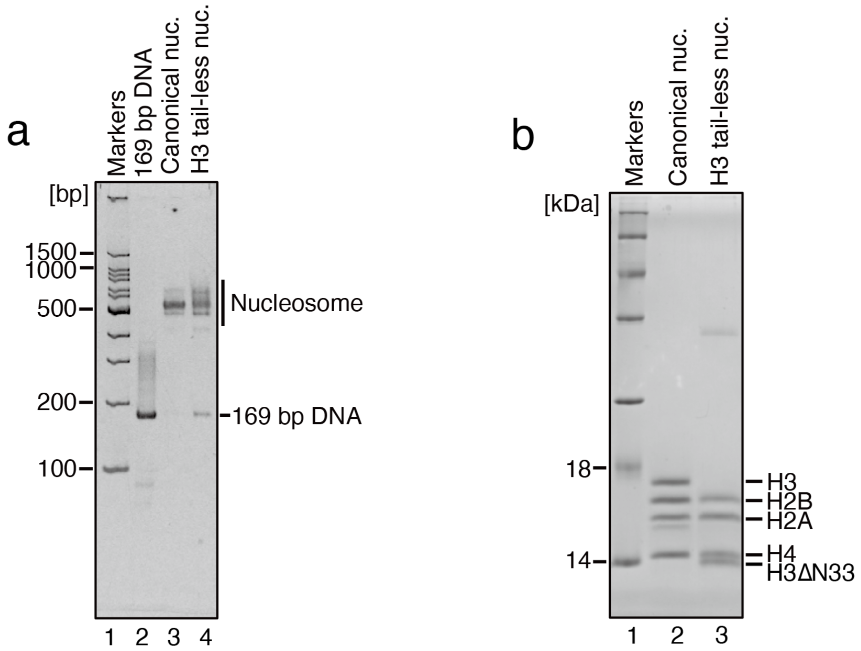

Supplementary Fig. 8. Preparation of the H3 tail-less nucleosome.
(a) Non-denaturing-PAGE analysis of the *K. phaffii* H3 tail-less nucleosome with ethidium bromide staining. The uncropped gel image is provided in Supplementary Fig. 19. (b) SDS-PAGE analysis of the *K. phaffii* H3 tail-less nucleosome with Coomassie Brilliant Blue staining. The uncropped gel image is provided in Supplementary Fig. 19.

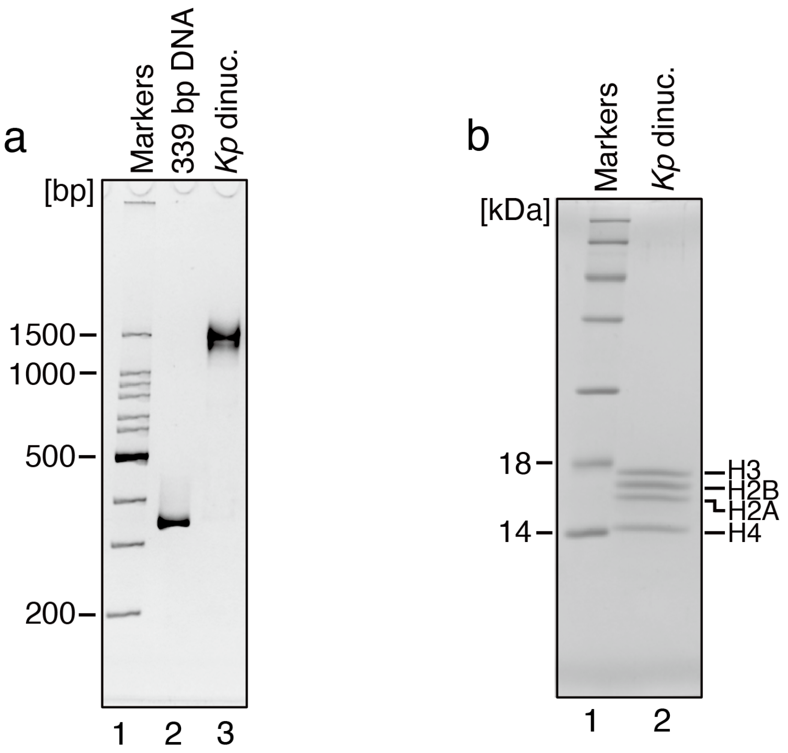

Supplementary Fig. 9. Preparation of di-nucleosomes for the nucleosome remodeling assay. (a) Non-denaturing-PAGE analysis of the *K. phaffii* di-nucleosome with ethidium bromide staining. The uncropped gel image is provided in Supplementary Fig. 19. (b) SDS-PAGE analysis of the purified *K. phaffii* di-nucleosome with Coomassie Brilliant Blue staining. The uncropped gel image is provided in Supplementary Fig. 19.

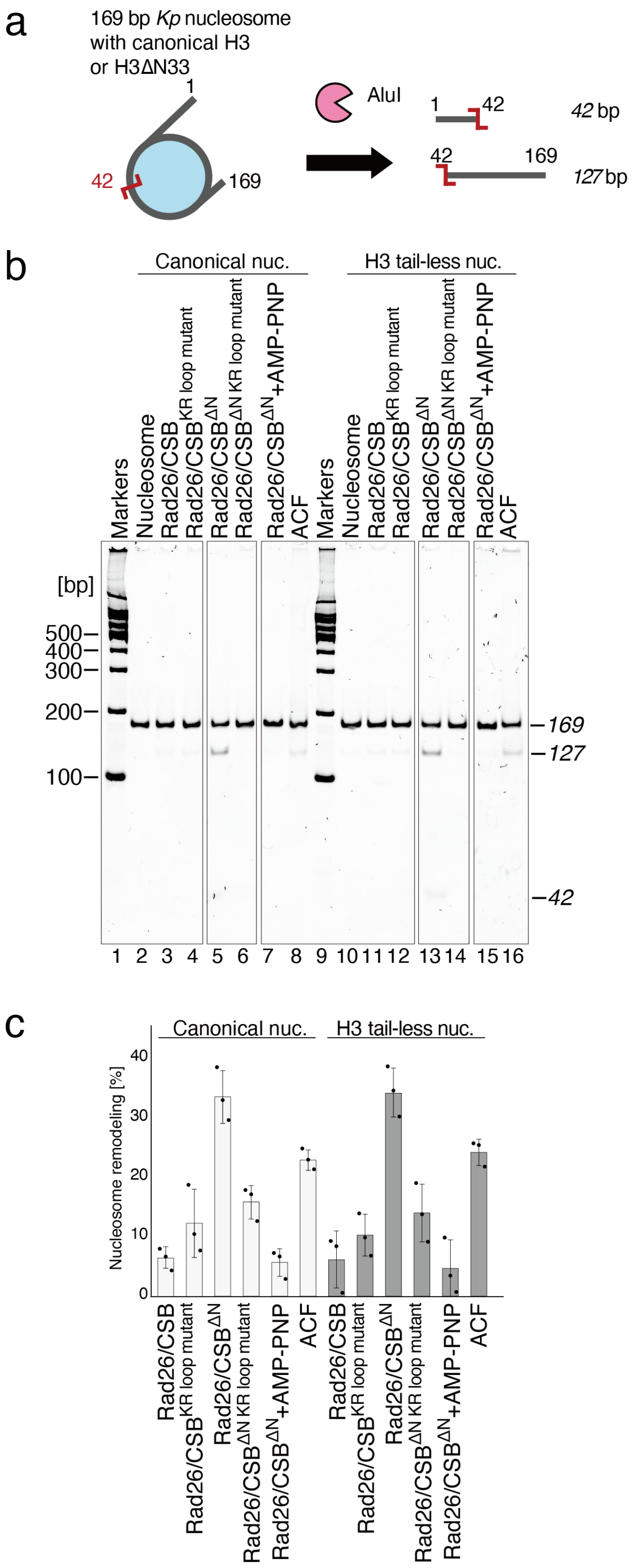
a
169 bp Kp nucleosome
with canonical H3
or H3ΔN33
AluI
42 bp
127 bp
b
Canonical nuc.
H3 tail-less nuc.
Markers
Nucleosome
Rad26/CSB
Rad26/CSB KR loop mutant
Rad26/CSB ΔN
Rad26/CSB ΔN KR loop mutant
Rad26/CSB ΔN +AMP-PNP
ACF
[bp]
500
400
300
200
100
169
127
42
1 2 3 4 5 6 7 8 9 10 11 12 13 14 15 16
c
Canonical nuc.
H3 tail-less nuc.
Nucleosome remodeling [%]
0
10
20
30
40

Supplementary Fig. 10. Nucleosome remodeling assay of the H3 tail-less nucleosome.
a, Schematic representation of the nucleosome remodeling assay of the H3 tail-less nucleosome. In the 169 bp nucleosome substrate used in this assay, one of the *Alu*I restriction enzyme sites is concealed by nucleosome formation, but becomes accessible when nucleosome remodeling occurs. Expected DNA fragments resulting from *Alu*I digestion are presented in the panel. **b**, The nucleosome remodeling assay. The nucleosome remodeling reaction using the canonical nucleosome was conducted in the presence of Rad26/CSB (lane 3), Rad26/CSB KR loop mutant (lane 4), Rad26/CSBΔN (lane 5), Rad26/CSBΔN KR loop mutant (lane 6), and ACF (lane 8; a positive control). For a negative control, the reaction was conducted in the presence of Rad26/CSB and AMP-PMP (lane 7). Lanes 1 and 2 are DNA markers and the nucleosome substrate, respectively. The nucleosome remodeling reaction using the H3 tail-less nucleosome was conducted in the presence of Rad26/CSB (lane 11), Rad26/CSB KR loop mutant (lane 12), Rad26/CSBΔN (lane 13), Rad26/CSBΔN KR loop mutant (lane 14), and ACF (lane 16; a positive control). For a negative control, the reaction was conducted in the presence of Rad26/CSB and AMP-PMP (lane 15). Lane 9 is DNA markers and the nucleosome substrate. Experiments were independently replicated three times, and these results are shown in Supplementary Fig. 17. **c**, Quantitation of the nucleosome remodeling activity. The nucleosome remodeling ratios (%) were estimated relative to the band intensity of free nucleosomes (lane 2). Average ratios of three independent experiments (Supplementary Fig. 10b and Supplementary Fig. 17) were plotted with SD values. Source data are provided as a Source Data file.

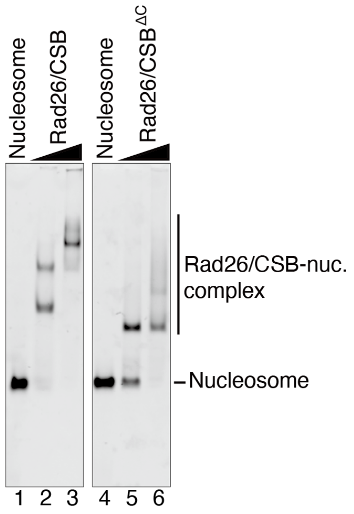

Supplementary Fig. 11. Nucleosome binding assay of Rad26/CSBΔC.
The Rad26/CSB-nucleosome complexes were detected by non-denaturing PAGE with ethidium bromide staining. Lanes 1 and 4 indicate the nucleosome (0.5 μM). Lanes 2-3 and 5-6 indicate the experiments with Rad26/CSB and Rad26/CSBΔX, respectively. The protein concentrations of Rad26/CSB are 0.25 and 0.5 μM. Experiments were independently replicated twice, and these results are shown in Supplementary Fig. 19. The uncropped gel image is provided in Supplementary Fig. 19.

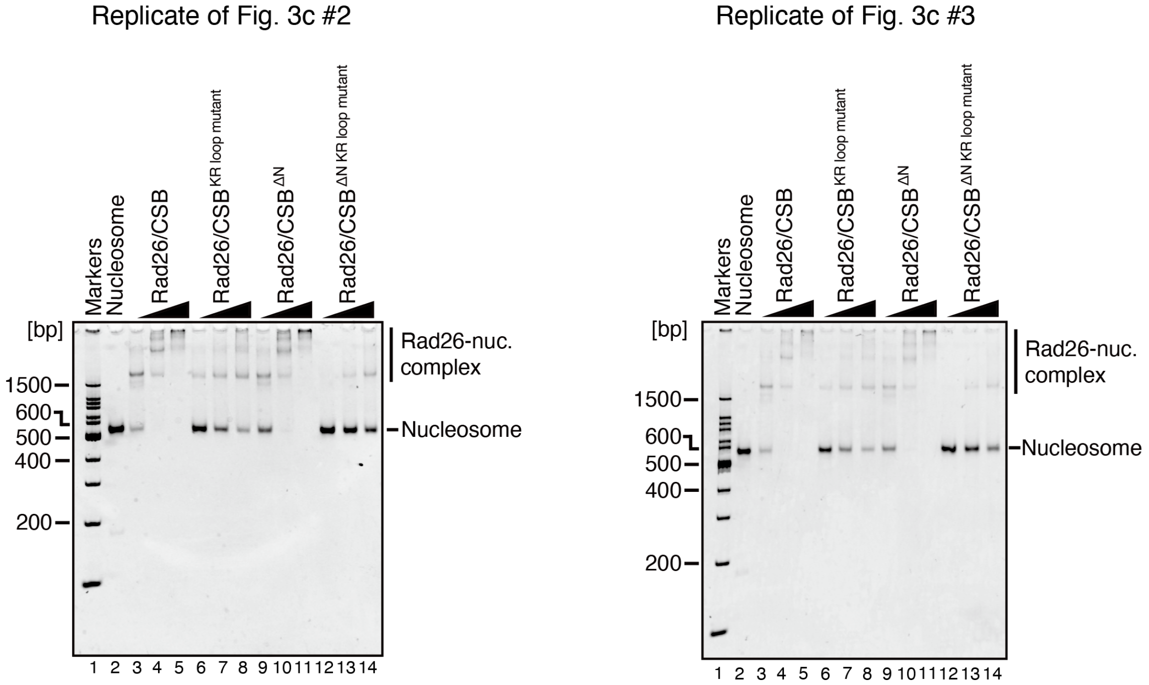

Supplementary Fig. 12. Replicated experiments of the nucleosome binding assay with Rad26/CSB mutants, as shown in Fig. 3c. Uncropped gel images are provided in Supplementary Fig. 19.

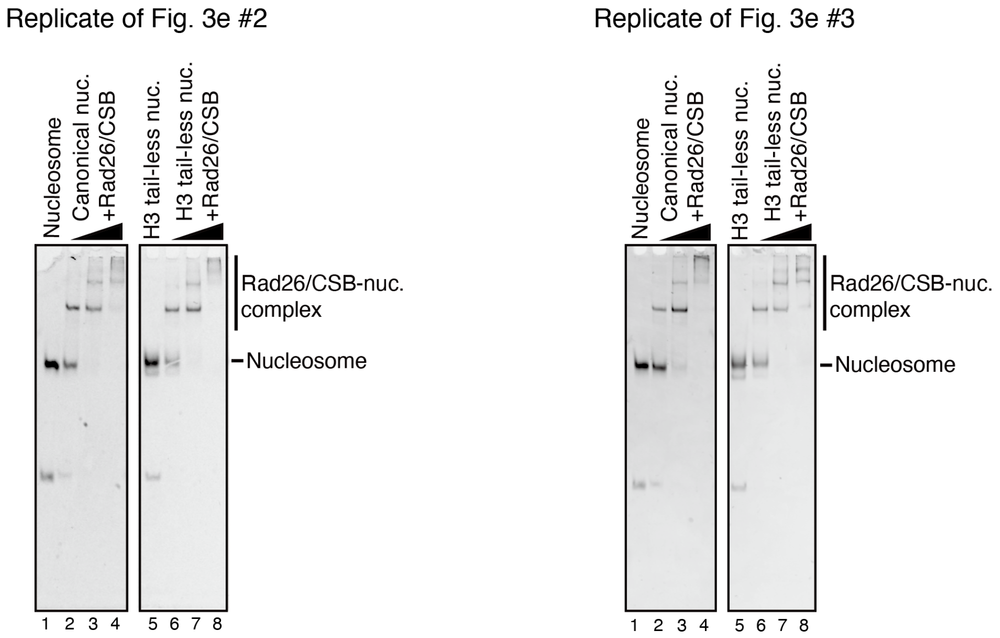

Supplementary Fig. 13. Replicated experiments of the H3 tail-less nucleosome binding assay with Rad26/CSB mutants, as shown in Fig. 3e. Uncropped gel images are provided in Supplementary Fig. 19.

Replicate of Fig. 5b #2

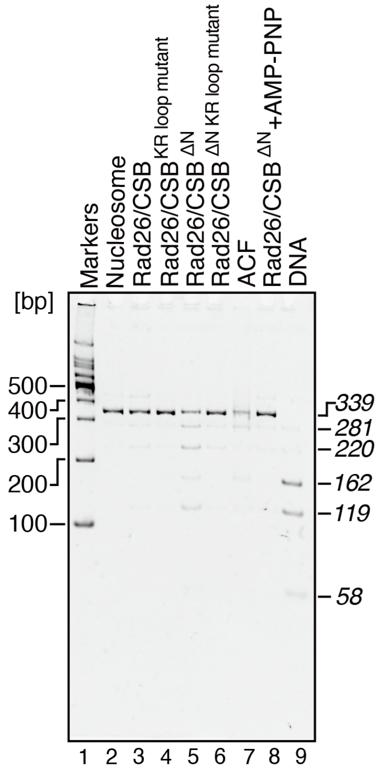

Replicate of Fig. 5b #3

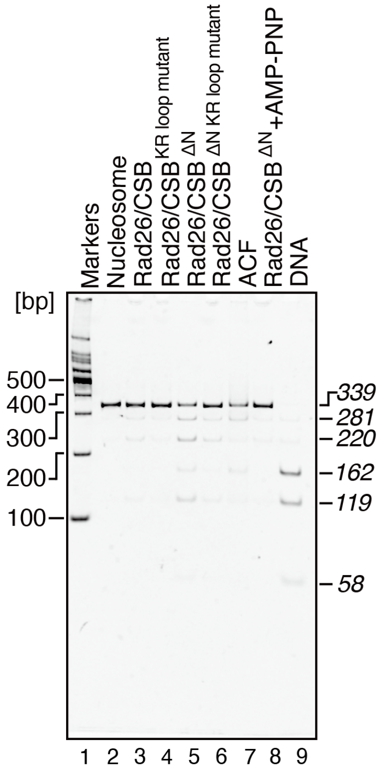

Supplementary Fig. 14. Replicated experiments of the nucleosome remodeling assay with Rad26 mutants, as shown in Fig. 5b. Uncropped gel images are provided in Supplementary Fig. 19.

Replicate of Fig. 5d #2

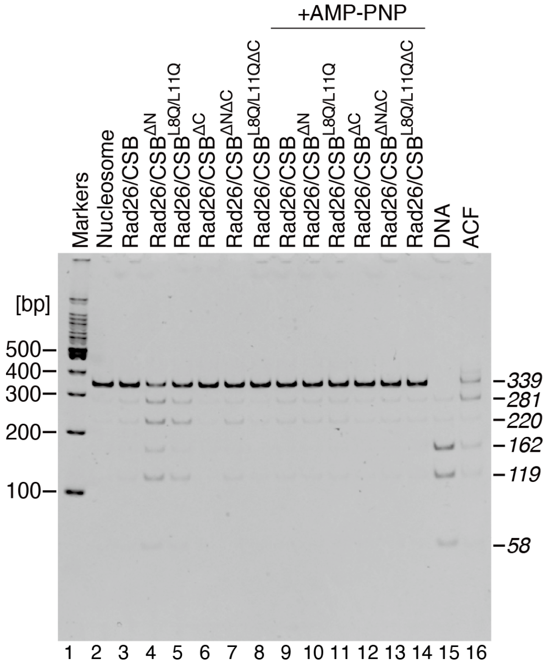

Replicate of Fig. 5d #3

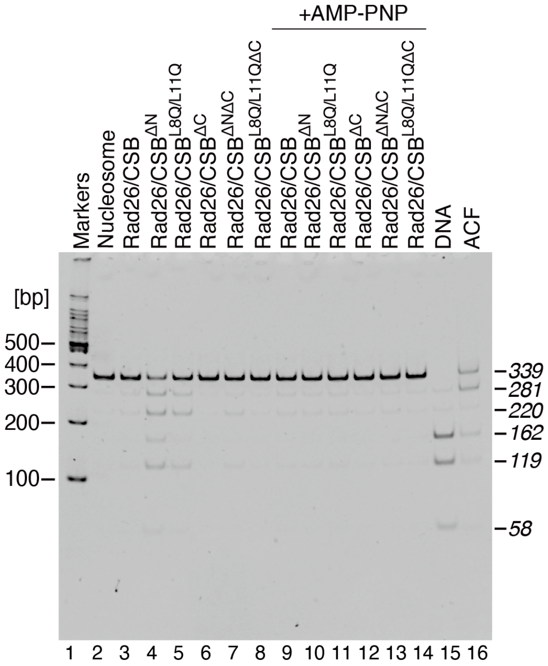

Supplementary Fig. 15. Replicated experiments of the nucleosome remodeling assay with Rad26 mutants, as shown in Fig. 5d. Uncropped gel images are provided in Supplementary Fig. 19.

Replicate of Suplemental Fig. 10 #2

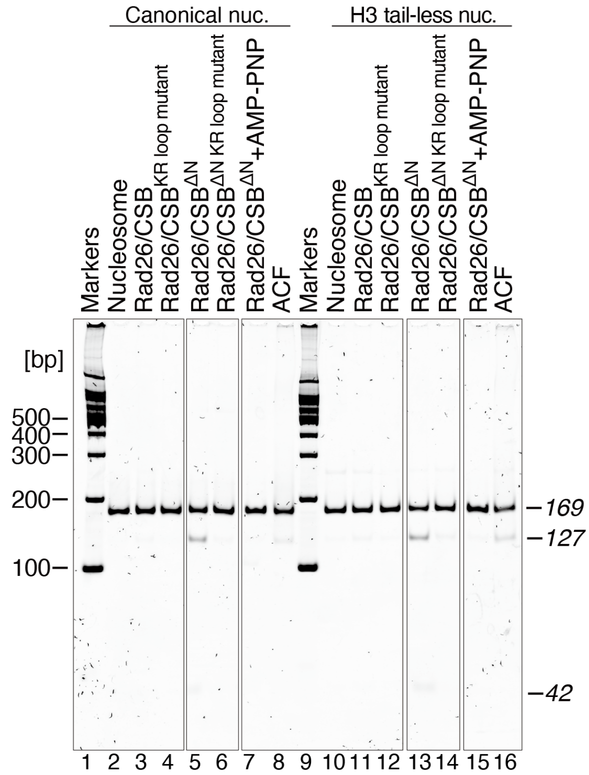

Replicate of Suplemental Fig. 10 #3

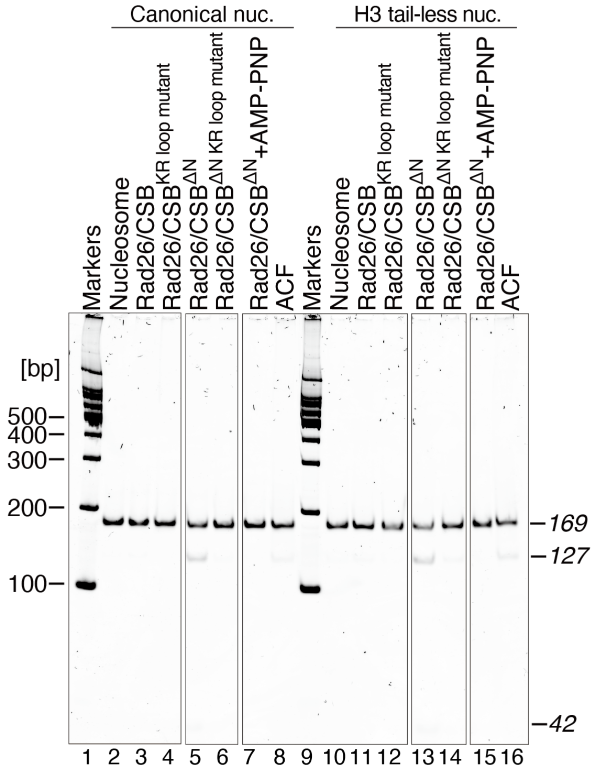

Supplementary Fig. 16. Replicated experiments of the nucleosome remodeling assay with H3 tail-less nucleosome, as shown in Supplementary Fig. 10. Uncropped gel images are provided in Supplementary Fig. 19.

Replicate of Supplementary Fig. 15 #2

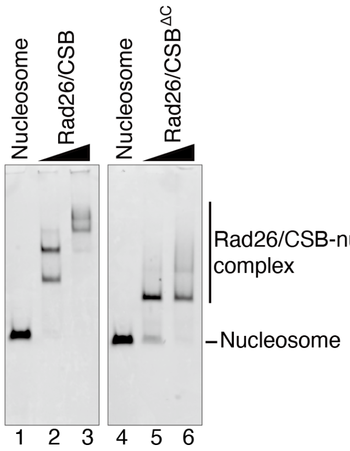

Supplementary Fig. 17. Replicated experiments of the nucleosome binding assay of Rad26/CSBΔC, as shown in Supplementary Fig. 12. Uncropped gel images are provided in Supplementary Fig. 19.

a

Id	Protein1	Protein2	Type	XLType	AbsPos1	AbsPos2	Id-Score
LNAKGAEEEDFVPFK-KLLELWK-a4-b1	Rad26/CSB	Rad26/CSB	xlink	intra-protein xl	695	660	42.26
VKELIDSVFTR-SLASLKAESR-a2-b6	Rad26/CSB	Rad26/CSB	xlink	intra-protein xl	430	424	39.69
ELKDIEENELK-NTKISER-a3-b3	Rad26/CSB	Rad26/CSB	xlink	intra-protein xl	85	73	37.15
LQETMKASSLK-DDLKEINTR-a6-b4	Rad26/CSB	Rad26/CSB	xlink	intra-protein xl	111	100	36.04
VLRDDLKEINTR-TKSQIQK-a7-b2	Rad26/CSB	Rad26/CSB	xlink	intra-protein xl	100	50	35.43
SLESKINSR-NTKISER-a5-b3	Rad26/CSB	Rad26/CSB	xlink	intra-protein xl	66	73	35.12
LNAKGAEEEDFVPFK-MQVVKK-a4-b5	Rad26/CSB	Rad26/CSB	xlink	intra-protein xl	695	659	34.19
VLRDDLKEINTR-LQETMKASSLK-a7-b6	Rad26/CSB	Rad26/CSB	xlink	intra-protein xl	100	111	33.82
SLASLKAESR-NTKISER-a6-b3	Rad26/CSB	Rad26/CSB	xlink	intra-protein xl	424	73	32.82
TSLQNEASIEKLLEEEEYGSTK-SLASLKAESR-a11-b6	Rad26/CSB	Rad26/CSB	xlink	intra-protein xl	407	424	32.72
SLESKINSR-ILQGKR-a5-b5	Rad26/CSB	Rad26/CSB	xlink	intra-protein xl	66	612	31.83
AKETLDEVEQLPNETK-LQETMKASSLK-a2-b6	Rad26/CSB	Rad26/CSB	xlink	intra-protein xl	118	111	31.66
NTKISERER-SLESKINSR-a3-b5	Rad26/CSB	Rad26/CSB	xlink	intra-protein xl	73	66	30.47
SLESKINSR-WQSKQR-a5-b4	Rad26/CSB	Rad26/CSB	xlink	intra-protein xl	66	59	30.11

Id	Protein1	Protein2	Type	XLType	AbsPos1	AbsPos2	Id-Score
KSAPSAAGGVK-SLASLKAESR-a1-b6	H3	Rad26/CSB	xlink	inter-protein xl	29	424	32.55
KSAPSAAGGVK-ILQGKR-a1-b5	H3	Rad26/CSB	xlink	inter-protein xl	29	612	30.29

b

Id	Protein1	Protein2	Type	XLType	AbsPos1	AbsPos2	Id-Score
SLESKINSR-STGGKAPR-a5-b5	Rad26/CSB	H3	xlink	inter-protein xl	66	16	20.32
VKADVAKDLPK-KDILLAR-a7-b1	Rad26/CSB	H3	xlink	inter-protein xl	574	125	18.41
KSAPSAAGGVK-ADVAKDLPK-a1-b5	H3	Rad26/CSB	xlink	inter-protein xl	29	574	18.05

Id	Protein1	Protein2	Type	XLType	AbsPos1	AbsPos2	Id-Score
SLESKINSR-NTKISER-a5-b3	Rad26/CSB	Rad26/CSB	xlink	intra-protein xl	66	73	30.91
TSLQNEASIEKLLEEEEYGSTK-SLASLKAESR-a11-b6	Rad26/CSB	Rad26/CSB	xlink	intra-protein xl	407	424	24.28
SLESKINSR-SQIQKWQSK-a5-b5	Rad26/CSB	Rad26/CSB	xlink	intra-protein xl	66	55	22.91
SLESKINSR-WQSKQR-a5-b4	Rad26/CSB	Rad26/CSB	xlink	intra-protein xl	66	59	21.16
NTKISERER-SLESKINSR-a3-b5	Rad26/CSB	Rad26/CSB	xlink	intra-protein xl	73	66	20.06
ILKDPKQR-DLDTKK-a3-b5	Rad26/CSB	Rad26/CSB	xlink	intra-protein xl	813	1013	18.92
DLDTKKK-DLDTKK-a5-b5	Rad26/CSB	Rad26/CSB	xlink	intra-protein xl	1013	1013	18.85

Id	Protein1	Protein2	Type	XLType	AbsPos1	AbsPos2	Id-Score
SLESKINSR-NTKISER-a5-b3	Rad26/CSB	Rad26/CSB	xlink	intra-protein xl	66	73	30.91
TSLQNEASIEKLLEEEEYGSTK-SLASLKAESR-a11-b6	Rad26/CSB	Rad26/CSB	xlink	intra-protein xl	407	424	24.28
SLESKINSR-SALEKGSR-a5-b5	Rad26/CSB	Rpb2	xlink	inter-protein xl	66	238	24.17
SLESKINSR-SQIQKWQSK-a5-b5	Rad26/CSB	Rad26/CSB	xlink	intra-protein xl	66	55	22.91
SLESKINSR-WQSKQR-a5-b4	Rad26/CSB	Rad26/CSB	xlink	intra-protein xl	66	59	21.16
SXLKQICK-KAMSSR-a4-b1	Rad26/CSB	Rpb2	xlink	inter-protein xl	1069	465	20.76
DHFGKK-RLEKTK-a5-b4	Rpb2	Rad26/CSB	xlink	inter-protein xl	397	48	20.73
NTKISERER-SLESKINSR-a3-b5	Rad26/CSB	Rad26/CSB	xlink	intra-protein xl	73	66	20.06
ATTLRLKHGTYEK-ILNKQR-a7-b4	Rpb2	Rad26/CSB	xlink	inter-protein xl	887	985	19.15
ILKDPKQR-DLDTKK-a3-b5	Rad26/CSB	Rad26/CSB	xlink	intra-protein xl	813	1013	18.92
DLDTKKK-DLDTKK-a5-b5	Rad26/CSB	Rad26/CSB	xlink	intra-protein xl	1013	1013	18.85

c

Id	Protein1	Protein2	Type	XLType	AbsPos1	AbsPos2	Id-Score
VLRDDLKEINTR-TKSQIQK-a7-b2	Rad26/CSBL8Q11Q	Rad26/CSBL8Q11Q	xlink	intra-protein xl	100	50	37.81
SLASLKAESR-SLASLKAESR-a6-b6	Rad26/CSBL8Q11Q	Rad26/CSBL8Q11Q	xlink	intra-protein xl	424	424	36.02
LNAKGAEEEDFVPFK-MQVVKK-a4-b5	Rad26/CSBL8Q11Q	Rad26/CSBL8Q11Q	xlink	intra-protein xl	695	659	35.73
LNAKGAEEEDFVPFK-KLLELWK-a4-b1	Rad26/CSBL8Q11Q	Rad26/CSBL8Q11Q	xlink	intra-protein xl	695	660	35
VKELIDSVFTR-SLASLKAESR-a2-b6	Rad26/CSBL8Q11Q	Rad26/CSBL8Q11Q	xlink	intra-protein xl	430	424	33.4
SLESKINSR-WQSKQR-a5-b4	Rad26/CSBL8Q11Q	Rad26/CSBL8Q11Q	xlink	intra-protein xl	66	59	32.52
SLESKINSR-NTKISER-a5-b3	Rad26/CSBL8Q11Q	Rad26/CSBL8Q11Q	xlink	intra-protein xl	66	73	32.41
VKELIDSVFTR-IYSKHLLK-a2-b4	Rad26/CSBL8Q11Q	Rad26/CSBL8Q11Q	xlink	intra-protein xl	430	455	32.08
NTKISERER-SLESKINSR-a3-b5	Rad26/CSBL8Q11Q	Rad26/CSBL8Q11Q	xlink	intra-protein xl	73	66	31.64
TSLQNEASIEKLLEEEEYGSTK-SLASLKAESR-a11-b6	Rad26/CSBL8Q11Q	Rad26/CSBL8Q11Q	xlink	intra-protein xl	407	424	31.22
SLESKINSR-SQIQKWQSK-a5-b5	Rad26/CSBL8Q11Q	Rad26/CSBL8Q11Q	xlink	intra-protein xl	66	55	30.45
LNAWVTKR-NTKISER-a7-b3	Rad26/CSBL8Q11Q	Rad26/CSBL8Q11Q	xlink	intra-protein xl	263	73	30.39
ELKDIEENELK-NTKISER-a3-b3	Rad26/CSBL8Q11Q	Rad26/CSBL8Q11Q	xlink	intra-protein xl	85	73	30.13

d

Id	Protein1	Protein2	Type	XLType	AbsPos1	AbsPos2	Id-Score
VKELIDSVFTR-SLASLKAESR-a2-b6	Rad26/CSBdeltaC	Rad26/CSBdeltaC	xlink	intra-protein xl	430	424	40.31
TSLQNEASIEKLLEEEEYGSTK-SLASLKAESR-a11-b6	Rad26/CSBdeltaC	Rad26/CSBdeltaC	xlink	intra-protein xl	407	424	38.95
LNAKGAEEEDFVPFK-MQVVKK-a4-b5	Rad26/CSBdeltaC	Rad26/CSBdeltaC	xlink	intra-protein xl	695	659	37.55
VLRDDLKEINTR-TKSQIQK-a7-b2	Rad26/CSBdeltaC	Rad26/CSBdeltaC	xlink	intra-protein xl	100	50	37.16
SLESKINSR-NTKISER-a5-b3	Rad26/CSBdeltaC	Rad26/CSBdeltaC	xlink	intra-protein xl	66	73	36
LNAKGAEEEDFVPFK-KLLELWK-a4-b1	Rad26/CSBdeltaC	Rad26/CSBdeltaC	xlink	intra-protein xl	695	660	35.96
VLRDDLKEINTR-LQETMKASSLK-a7-b6	Rad26/CSBdeltaC	Rad26/CSBdeltaC	xlink	intra-protein xl	100	111	35.66
SLESKINSR-LNAWVTKR-a5-b7	Rad26/CSBdeltaC	Rad26/CSBdeltaC	xlink	intra-protein xl	66	263	34.82
AKETLDEVEQLPNETK-LQETMKASSLK-a2-b6	Rad26/CSBdeltaC	Rad26/CSBdeltaC	xlink	intra-protein xl	118	111	33.14
NTKISER-ILQGKR-a3-b5	Rad26/CSBdeltaC	Rad26/CSBdeltaC	xlink	intra-protein xl	73	612	33.07
LQETMKASSLK-DKELELK-a6-b2	Rad26/CSBdeltaC	Rad26/CSBdeltaC	xlink	intra-protein xl	111	39	32.22
SLESKINSR-ILQGKR-a5-b5	Rad26/CSBdeltaC	Rad26/CSBdeltaC	xlink	intra-protein xl	66	612	32.21
VKADVAKDLPK-KSEMVLFCK-a2-b1	Rad26/CSBdeltaC	Rad26/CSBdeltaC	xlink	intra-protein xl	569	579	32.01
ELKDIEENELK-NTKISER-a3-b3	Rad26/CSBdeltaC	Rad26/CSBdeltaC	xlink	intra-protein xl	85	73	32
SLESKINSR-WQSKQR-a5-b4	Rad26/CSBdeltaC	Rad26/CSBdeltaC	xlink	intra-protein xl	66	59	31.83
TSLQNEASIEKLLEEEEYGSTK-VKELIDSVFTR-a11-b2	Rad26/CSBdeltaC	Rad26/CSBdeltaC	xlink	intra-protein xl	407	430	31.2
SLASLKAESR-ILQGKR-a6-b5	Rad26/CSBdeltaC	Rad26/CSBdeltaC	xlink	intra-protein xl	424	612	31.07
TKSQIQK-TKSQIQK-a2-b2	Rad26/CSBdeltaC	Rad26/CSBdeltaC	xlink	intra-protein xl	50	50	30.2

Supplementary Fig. 18. List of crosslinked peptides identified by crosslinking mass spectrometry, as illustrated in Fig. 4. (a) Crosslinked peptides corresponding to Fig. 4a and the top panel of Fig. 4e. (b) Crosslinked peptides corresponding to Fig. 4c, f and g. (c) Crosslinked peptides corresponding to the middle panel of Fig. 4e. (d) Crosslinked peptides corresponding to the bottom panel of Fig. 4e.

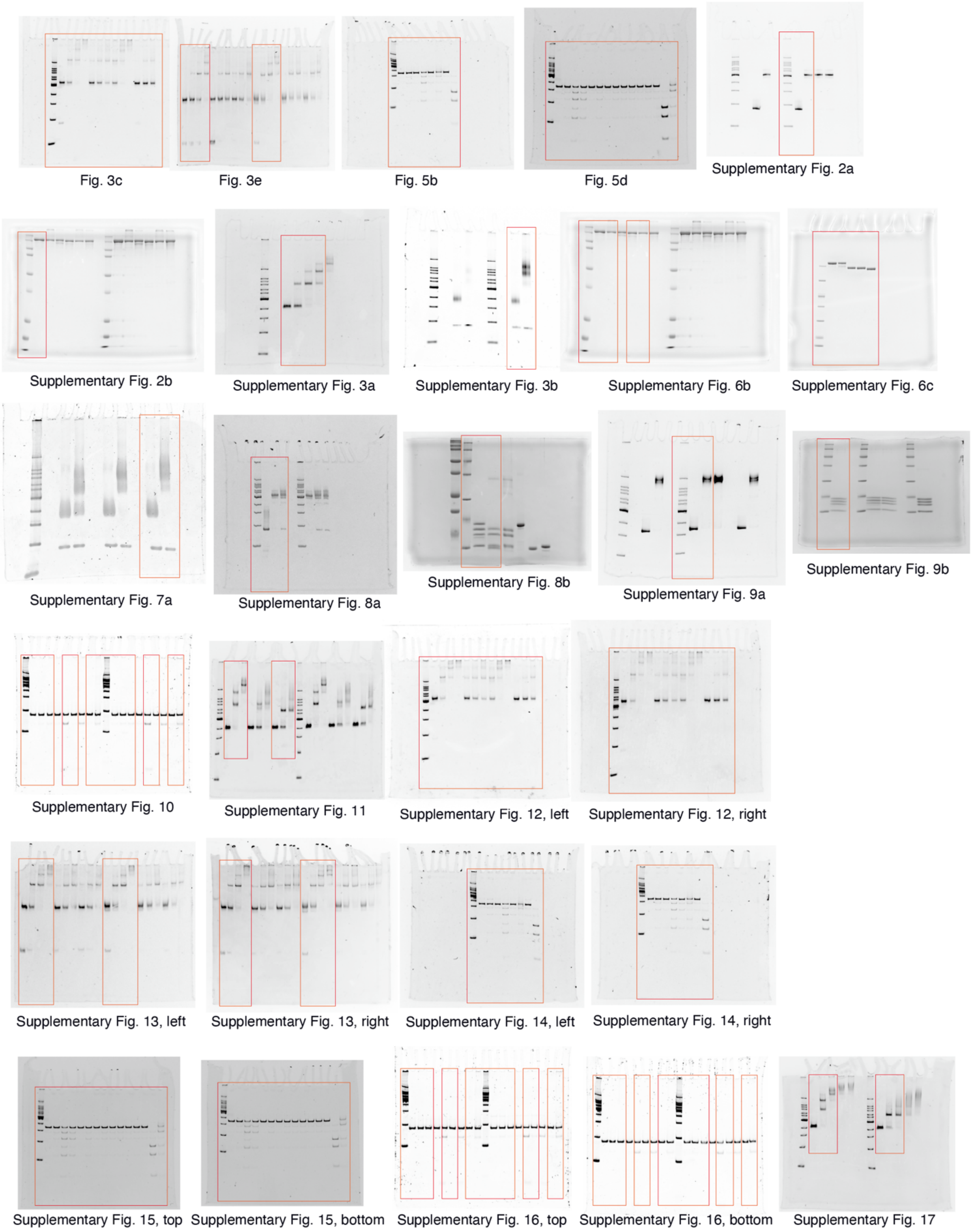

Supplementary Fig. 19. Uncropped gel images.

Supplementary Table 1: DNA sequence of *K. phaffii* nucleosomes used for cryo-EM structural analysis and remodelling assay.

601 DNA (169 bp)	CTGAGAATCCCGGTGCCGAGGCCGCTCAATTGGTCGTA<br>GAC**AGCT**CTAGCACCGCTTAAACGCACGTACGCGCTGT<br>CCCCCGCGTTTTAACCGCCAAGGGGATTACTCCCTAGTC<br>TCCAGGCACGTGTCAGATATATACATCCAGGCCTTGTGT<br>CGCGAAATTCATAGA
601 x2 DNA (339 bp)<br>Containing 15 bp linker<br>DNA x3, 601 DNA x2	ATCGGACCCTATCGCGAGGAGAATCCCGGTGCCGAGGC<br>CGCTCAATTGGTCGTAGAC**AGCT**CTAGCACCGCTTAAA<br>CGCACGTACGCGCTGTCCCCCGCGTITTAACCGCCAAGG<br>GGATTACTCCCTAGTCTCCAGGCACGTGTCAGATATATA<br>CATCCAGGCCTTGTGTAGCCAGGCCTGAGGGTCCCGGT<br>GCCGAGGCCGCTCAATTGGTCGTAGAC**AGCT**CTAGCAC<br>CGCTTAAACGCACGTACGCGCTGTCCCCCGCGTTTTAAC<br>CGCCAAGGGGATTACTCCCTAGTCTCCAGGCACGTGTC<br>AGATATATACATCCGTCGCGAAATTCATAGAT

*Alu*I recognition site on the 601 DNA sequence: **AGCT**

Supplementary Table 2: Cryo-EM image processing of *K. phaffii* Rad26-nucleosome complex

Sample	*K. phaffii* Rad26-nucleosome complex (EMDB: EMD-63262, PDB: 9LOX)	*K. phaffii* Rad26ΔN-nucleosome complex (EMDB: EMD-63507)	
**Data collection**			
Electron microscope	Krios G4	Krios G4	
Camera	K3	K3	
Pixel size (Å/pix)	1.06	1.06	
Defocus range (µm)	-1.0 to -2.5	-1.0 to -2.5	
Exposure time (second)	5.6	5.6	
Total dose (e/Å^2)	59.7	61.8	63.8
Movie frames (no.)	40	40	
Total micrographs (no.)	10,264	5,001	5,366
**Image processing**			
Software	Relion 4.0-beta	Relion 4.0-beta	
Particles for 2D classification	4,747,760	478,137	536,744
Particles for 3D classification	3,425,355	621,299	
Particles in the final map (no.)	36,207	226,294	
Symmetry	C1	C1	
Final resolution (Å)	3.5	4.63	
FSC threshold	0.143	0.143	
Map sharpening B factor (Å^2)	-38.3975		

Supplementary Table 3: Construction of the atomic model of the *K. phaffii* Rad26-nucleosome complex

sample	*K. phaffii* Rad26- nucleosome complex
**Model building**	
Software	AlphaFold3, PyMOL, ISOLDE
**Refinement**	
Software	Phenix, ISOLDE
**Model composition**	
Protein	1272
Nucleotide	284
**Validation**	
MolProbity score	1.44
Clash score	8.03
**R.m.s. deviations**	
Bond lengths (Å)	0.005
Bond angles (˚)	0.706
**Ramachandran plot**	
Favored (%)	98.16
Allowed (%)	1.84
Outliers (%)	0
