# Supplementary material for: Structural basis of nucleosome remodeling by Cockayne syndrome B homologue Komagataella phaffii Rad26
Source: Nat Commun. 2026 Jun 24;17:4881. doi: 10.1038/s41467-026-73500-7 (PMC13294370; doi:10.1038/s41467-026-73500-7)
Supplement: Supplementary file 4 — Source Data [file 41467_2026_73500_MOESM4_ESM.zip › Source_Data_uncropped_gel.pdf]

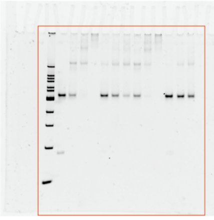
Fig. 3c

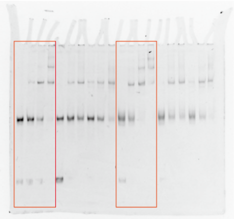
Fig. 3e

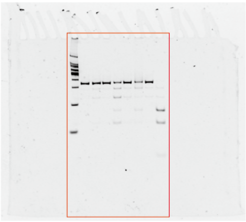
Fig. 5b

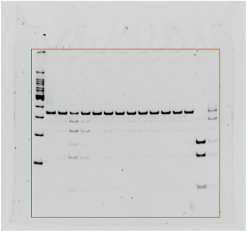
Fig. 5d

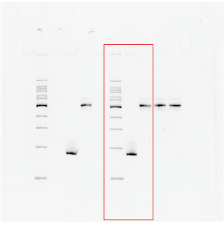
Supplementary Fig. 2a

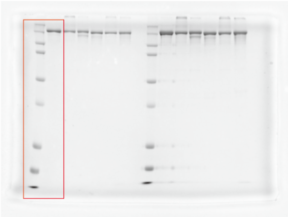
Supplementary Fig. 2b

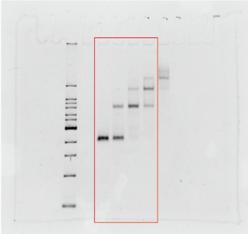
Supplementary Fig. 3a

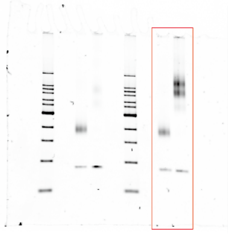
Supplementary Fig. 3b

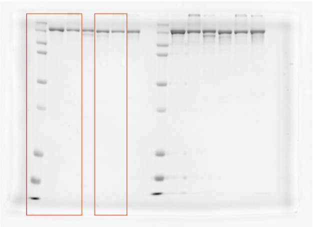
Supplementary Fig. 6b

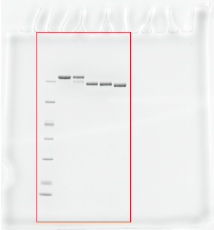
Supplementary Fig. 6c

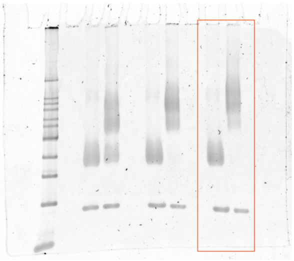
Supplementary Fig. 7a

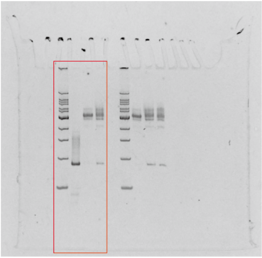
Supplementary Fig. 8a

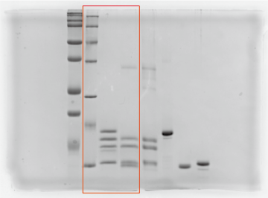
Supplementary Fig. 8b

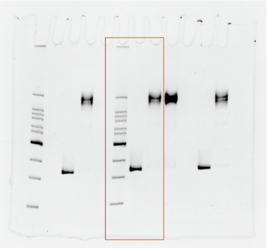
Supplementary Fig. 9a

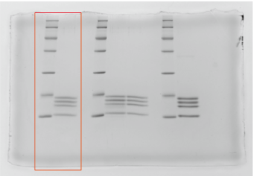
Supplementary Fig. 9b

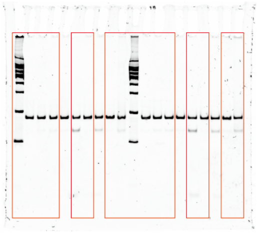
Supplementary Fig. 10

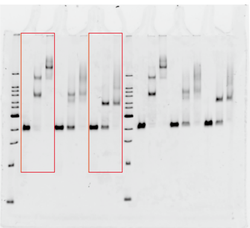
Supplementary Fig. 11

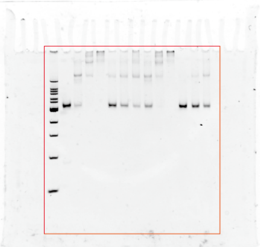
Supplementary Fig. 12, left

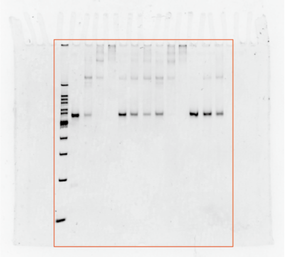
Supplementary Fig. 12, right

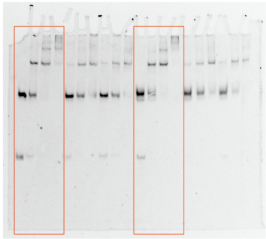
Supplementary Fig. 13, left

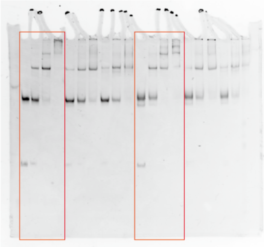
Supplementary Fig. 13, right

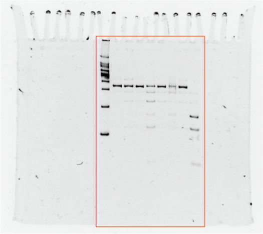
Supplementary Fig. 14, left

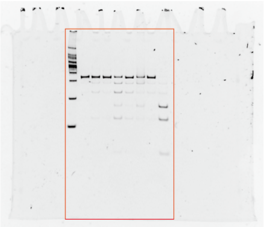
Supplementary Fig. 14, right

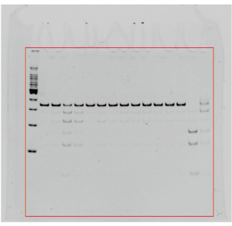
Supplementary Fig. 15, top

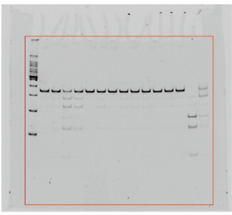
Supplementary Fig. 15, bottom

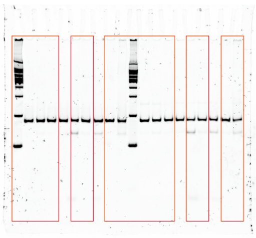
Supplementary Fig. 16, top

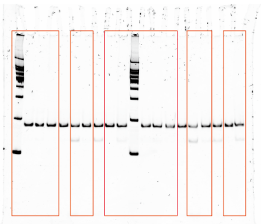
Supplementary Fig. 16, bottom

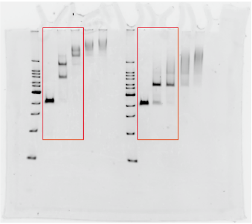
Supplementary Fig. 17
