# Supplementary material for: Structural basis of nucleosome remodeling by Cockayne syndrome B homologue Komagataella phaffii Rad26
Source: Nat Commun. 2026 Jun 24;17:4881. doi: 10.1038/s41467-026-73500-7 (PMC13294370; doi:10.1038/s41467-026-73500-7)
Supplement: Supplementary file 3 — Transparent Peer Review file [file 41467_2026_73500_MOESM3_ESM.pdf]

nature portfolio

Peer Review File

Structural basis of nucleosome remodeling by Cockayne syndrome B homologue Komagataella phaffii Rad26

Corresponding Author: Professor Hitoshi Kurumizaka

This file contains all reviewer reports in order by version, followed by all author rebuttals in order by version.

Version 0:

Reviewer comments:

Reviewer #1

(Remarks to the Author)
In this manuscript, Fukushima et al. present the cryo-EM structure of Rad26 in complex with the nucleosome, providing insights into its role in transcription-coupled nucleotide excision repair. The authors combine a cryo-EM reconstruction with an AlphaFold-predicted model to depict how Rad26 recognizes the nucleosome and potentially functions as a chromatin remodeler within the ATP-dependent remodeling family. Notably, they highlight that Rad26 exhibits a distinct mode of nucleosome engagement compared to other remodelers, and propose a potential role for the KR-loop in recognizing RNA polymerase II and mediating nucleosome binding and remodeling.
While the structural data reasonably support the authors' claims regarding Rad26's unique remodeling mechanism and the involvement of the KR-loop, the manuscript contains several inconsistencies and unclear interpretations that must be addressed. Therefore, revision is recommended to strengthen the scientific rigor and clarity of the study.
Detailed recommendations for revision are provided below. These improvements are essential to enhance the manuscript's readability and to advance understanding of Rad26's biological function.

Major points

1. The Coulomb density maps of Rad26 shown in Extended Data Fig. 4e and Fig. 1a appear notably different in quality. It seems the resolution for Rad26 may not be sufficient to confidently trace even the backbone. Could the authors clarify how the map in Fig. 1a was generated? Cryo-EM structures of nucleosome–protein complexes often exhibit higher resolution for the nucleosome core, while the binding partner tends to be less well-resolved. If focused refinement was performed specifically on Rad26, please report the corresponding gold-standard FSC resolution derived from that focused refinement.

2. Were the contacting residues of the KR loop in DNA versus H3 tail recognition distinguishable in your map? The KR loop appears to interact with both DNA and the histone H3 tail, but the specific residues involved are not clearly described. If the resolution does not permit unambiguous assignment of these contacts, this limitation should be explicitly noted in the main text.

3. The claim that "Our cryo-EM analysis confirmed that Rad26/CSB N binds to the SHL±6 position of the nucleosome, and the N-terminal deletion minimally affects the structure of the Rad26/CSB-nucleosome complex" is not fully convincing. The density maps presented in Extended Data Fig. 8d and f are not of sufficient quality to support this conclusion without ambiguity. Additional clarification or supporting evidence is needed to substantiate this structural comparison.

Minor points

1. Have you observed any particle populations with a 1:2 stoichiometry of nucleosome to Rad26? Given that Rad26 is reported to bind at both the +6 and –6 superhelical locations (SHLs), one might expect to detect complexes with two Rad26 molecules bound to a single nucleosome. If not, any possible explanation?

2. V633-H634-A635-K636-R637-R638-S639-K640-K641-D642 is hard to read. 633-VHAKRRSKKD-642 might be easier to look at the peptide sequence at the KR loop.

3. In Fig. 3b, the colors used for the H3 tail, lobe 1, and the KR loop appear quite similar, which may lead to confusion. To improve clarity, please consider using distinct color schemes for each feature to clearly differentiate them.

4. Although the authors state that the KR loop is conserved from yeast to humans, Extended Data Fig. 6a does not clearly support this claim. As the alignment data are not easily interpretable in their current form, please consider incorporating the relevant conservation information of Extended Data Fig 6a directly into Fig. 3a to allow readers to independently assess the degree of conservation.

5. In Extended Data Fig. 10, please label the panels as “Replicate of Fig. 3e #2” and “Replicate of Fig. 3e #3” to prevent potential confusion for readers.

Reviewer #2

(Remarks to the Author)
The manuscript presents high-quality structural work on the Rad26/CSB–nucleosome complex, offering new insights into how CSB engages chromatin during transcription-coupled nucleotide excision repair (TC-NER). The cryo-EM data are compelling and well analyzed. However, to fully appreciate the regulatory and mechanistic aspects of CSB/Rad26 function, the manuscript would benefit from greater integration of known and emerging roles of the N- and C-terminal regions of CSB, especially in relation to its interaction with RNA polymerase II (RNAPII) and other TC-NER factors. Below are specific suggestions to expand the mechanistic depth of the work.

1. N-terminal Leucine Latch Motif: Autoinhibitory Function
The study mentions that the N-terminal region (NTR) of Rad26 includes an auto-inhibitory domain with a leucine latch motif (LxxL). While this motif is functionally significant (as its deletion activates remodeling activity), structural and mechanistic details of its interaction with the ATPase domain (Lobe 1 and Lobe 2) remain insufficiently explored in this work.

Recommendation:
Please expand your discussion on the regulatory role of the leucine latch. Incorporating structural modeling or mutagenesis experiments (L7G/L10G mutations) to test interaction with the ATPase domain would support a mechanistic understanding of auto-inhibition.
Further, crosslinking mass spectrometry (XL-MS) could be used to identify residues in the N-terminal region that contact lobe 2 in both nucleosome-bound and apo states in presence or absence AMP-PNP.
The C-terminal region CSB-specific coupling motif of Rhp26 is essential for chromatin remodeling (Wang et al., PNAS, 2014). This study doesn't explore this important point.
Wang et al., PNAS, 2014 showed that the remodeling activity of Rhp26 7P/10P leucine latch mutant is greatly reduced by H3 and H4 tail deletion. The activity of Rad26 leucine latch mutant should be investigated with H3/H4 tail less nucleosomes.

2. Residue Overlap Between RNAPII and ATPase/Nucleosome Interactions
The N-terminal domain of CSB is also known to interact with stalled RNAPII, yet it is unclear whether this involves the same residues as those mediating auto-inhibition or nucleosome binding.
Recommendation:
Clarify whether shared residues in the N-terminal region, possibly within or near the leucine latch, are involved in both RNAPII engagement and ATPase inhibition. This could be tested using comparative XL-MS of Rad26/CSB in complex with either RNAPII or nucleosome. Distinct or overlapping footprints would strongly inform models of CSB conformational switching.

3. C-terminal Domain: Role in RNAPII Interaction and Transcriptional Regulation
As demonstrated in Weems et al. (J. Biol. Chem., 2021), the C-terminal domain of CSB is essential for stable interaction with RNAPII and recruitment of the Elongin-CUL5 ubiquitin ligase complex, even under transcriptional stress (e.g., ER stress, hormone signaling), not just DNA damage.
Recommendation:
I recommend including the C-terminal domain in your structural or functional model, particularly to contextualize CSB’s dual role in DNA repair and RNAPII transcription regulation. Functional assays using C-terminal truncation mutants could help delineate its role in remodeling vs. transcriptional engagement.

4. Dynamic Competition Between RNAPII and Nucleosomes
The manuscript suggests Rad26 may function at both RNAPII-stalled complexes and nucleosomes during TC-NER, implying a dynamic exchange or handoff mechanism.
Recommendation:
I encourage the authors to perform time-resolved or competitive binding assays (e.g., FRET, biolayer interferometry) to assess whether RNAPII and nucleosomes bind Rad26/CSB sequentially or competitively. This would provide mechanistic support to your proposed model and clarify whether a mutually exclusive binding mode exists.

Final Recommendation
This manuscript presents a valuable structural contribution to our understanding of rad26/CSB function. To maximize its impact, I recommend a more integrated discussion of the N-terminal leucine latch motif, the C-terminal domain’s role in transcriptional regulation, and emerging mechanistic insights from recent structures of RNA polymerase II (Pol II)–stalled complexes.
A key question that remains insufficiently addressed is how Rad26/CSB transitions between Pol II– and nucleosome-bound states, a process central to its function in transcription-coupled nucleotide excision repair (TC-NER). Elucidating this switch in binding modes through further discussion and targeted experimentation would strengthen the conceptual framework of the study.
Moreover, several mechanistic conclusions would benefit from additional validation, such as mutagenesis or crosslinking experiments, to support proposed models of autoinhibition and activation.
Recommendation: Major revisions are required prior to acceptance.

Reviewer #3

(Remarks to the Author)
Rad26, a yeast homologue of mammalian Cockayne syndrome protein B (CSB), plays an essential role in transcription-coupled nucleotide excision repair. In addition, Rad26 also possesses nucleosome remodeling activity, but the mechanism is unclear. In this manuscript, Fukushima, Kurumizaka and colleagues took a combined biochemistry and cryo-EM structural approach to visualize the structure of the Rad26/CSB-nucleosome complex. The quality of cryoEM structure is elegant. They found that Rad26/CSB preferentially binds to the SHL 6 position of the nucleosomal DNA and distorts the entry/exit region of the nucleosome. The ATPase Lobe 1 and Lobe 2 domains bind to the nucleosome in the reverse orientation as compared to the Snf2 and Ino80 bound to the SHL 6 position. Finally, they found that the KR loop in Lobe 2 domain can function in nucleosome binding and remodeling, which is further supported by mutational analysis. Together, this study provides important insights into the remodeling function of Rad26 and suggest its potential functional role in transcription-coupled nucleotide excision repair.

I have a few minor concerns that should be addressed before publication.
1. In the results part, the authors found that the KR loop directly contacts the DNA at the SHL 6 position and the N-terminal tail of H3. This result is consistent with previous publication indicating the important roles of H3 and H4 tails for remodeling activity of Rad26 (Ref 22. Wang W et al PNAS 2019). On the other hand, the biochemical assay results (Fig 3e) in this manuscript show that Rad26 appears to exhibit higher affinity for H3 tail-less nucleosomes than WT nucleosomes, which appears to conflict with the structural observation of an interaction between the H3 tail and the KR loop of Rad26. How can this discrepancy be explained?

2. In the discussion section, the authors propose an interesting model that upon Rad26 recruit to arrested Pol II, then transfer to nucleosome ahead of Pol II and evicted the nucleosome and clear the room for TC-NER. While this is very provocative showing the role of remodeling activity of Rad26 in TC-NER, mammalian CSB also plays an important role to recruit downstream factors including CSA, UVSSA, and TFIIH. The relative position of Pol II-CSB/Rad26 are critical for these recruitment events. It remains unclear how this new model (proposed in this manuscript) will be integrated into current model of TC-NER. In addition, for normal elongation for a set of genes (no lesion) or with small lesions, previous publication suggests that CSB forms a stable complex with Pol II and acts as an ATP-dependent processivity factor, promoting Pol II progression through a nucleosome barrier (and/or small lesions) and promotes elongation on these genes (Xu J et al PNAS 2020). This previous model may be also integrated with current model to elucidate the roles of Rad26 (on lesion recognition) in both TC-NER and transcription elongation for certain genes.
3. For Fig. 3b, it would be easier for readers to better understand the interactions between H3 and Rad26-KR-loop by showing the side chains of KR loop and their interaction partners in H3 tail and DNA.
4. Line 125 “This suggest that the N-terminal…”, “This suggest” should be “This suggests”. It would be great for authors take a careful look at the grammar and spelling to ensure clarity.

Reviewer #4

(Remarks to the Author)
I co-reviewed this manuscript with one of the reviewers who provided the listed reports. This is part of the Nature Communications initiative to facilitate training in peer review and to provide appropriate recognition for Early Career Researchers who co-review manuscripts.

Reviewer #5

(Remarks to the Author)
I co-reviewed this manuscript with one of the reviewers who provided the listed reports. This is part of the Nature Communications initiative to facilitate training in peer review and to provide appropriate recognition for Early Career Researchers who co-review manuscripts.

Version 1:

Reviewer comments:

Reviewer #1

(Remarks to the Author)
Although authors claimed that “In the Rad26/CSB-nucleosome complex, the K636, R637, R638, K640, and K641 residues of the KR loop directly contact the DNA at the SHL±6 position (page 5, lines 113-115)”, the structural data in Fig. 3b and Supplementary Fig. 5b instead show that the KR loop primarily interacts with the H3 tails rather than with nucleosomal DNA. Mutating the KR loop to GS residues disrupted nucleosome binding, which the authors interpreted as evidence for direct engagement of the KR loop with nucleosomal DNA. However, the authors did not rule out an alternative explanation: the Rad26–nucleosome dissociation could also result from disrupting KR loop–H3 tail interactions. This alternative model also provides a plausible explanation for why H3-tail–less nucleosomes bind more strongly to Rad26—removal of the H3 tail would eliminate interference with KR loop–H3 tail interactions, allowing the KR loop to access nucleosomal DNA more directly.
This interpretation challenges the authors’ main conclusion that “the KR loop-H3 tail interaction is not essential for Rad26/CSB nucleosome binding and remodeling. (Page 5, line 131)”. Because the role of the KR loop in nucleosome binding is a central claim of the manuscript, the authors should unambiguously clarify this issue before publication.

In Supplementary Fig. 7d, Rad26ΔN (orange) appears to make extensive contacts with the H2A–H2B surface, a feature not observed in the Rad26–nucleosome structure (Fig. 1a). Supplementary Fig. 7h further suggests that this region exhibits distinct EM densities between the Rad26–nucleosome and Rad26ΔN–nucleosome complexes.

In Fig. 4a, Lys611 (Rad26) and H3K27 appear too far apart to support direct crosslinking in the XL-MS analysis.

In Supplementary Fig. 7h, the legend should include an explanation of the dark triangle annotation.

Reviewer #2

(Remarks to the Author)
All substantive concerns have been thoroughly addressed, and the revised manuscript is greatly improved. I have only one remaining minor point:
Histone H3 residues 35–39: As there is no strong supporting cryo-EM density for these residues, please remove them from the model. I think the density in that area is the contribution of R638 and other neighboring residues (636KRRSKK641) of chain K.

With this minor correction, the manuscript is suitable for publication.
Recommendation: Accept after minor revision.

Reviewer #3

(Remarks to the Author)
The revised manuscript has addressed all questions raised from previous version. Therefore I recommend it for publication.

Reviewer #4

(Remarks to the Author)
I co-reviewed this manuscript with one of the reviewers who provided the listed reports. This is part of the Nature Communications initiative to facilitate training in peer review and to provide appropriate recognition for Early Career Researchers who co-review manuscripts.

Reviewer #5

(Remarks to the Author)
I co-reviewed this manuscript with one of the reviewers who provided the listed reports. This is part of the Nature Communications initiative to facilitate training in peer review and to provide appropriate recognition for Early Career Researchers who co-review manuscripts.

Reviewer #6

(Remarks to the Author)
Comments to the Authors:
Overview:
Fukushima et al. presents a large body of work combining remodelling assays, cryoEM and XLMS to investigate the structure and role of Rad26 in TC-NER. The presented data largely supports their claims. However, additional information should be included and reviewed) with regards to the XLMS data prior to publication.

The cryoEM component has been extensively reviewed and commented on. The comments below will mostly focus on the new XLMS data.

1. Line142 “This enhancement of the Rad26/CSB-H3 interaction by RNAPII may function to stimulate the Rad26/CSB transfer from RNAPII to the nucleosome during the TC-NER process (discussed later).”
This statement seems rather speculative based on the presented XLMS data. Is there additional data to support this statement?

2. It is noted from Figure 4g that there appears to be an enhancement of N-terminal to C-terminus crosslinking of Rad26 in the presence of RNAPII.
Do the authors have any comments/insights on this observation? Do these crosslinks fit with the proposed model in Figure 6?

3. Related to point 2 above, were there other observed crosslinks from Rad26 to RNAPII and other proteins? Do these support your proposed model or provide additional insight?

4. How does all identified crosslinks map to the structure of Rad26 reported here, with and without nucleosome? Does the identified crosslinks fall within the crosslinker length of DSS? This can provide some insight into the quality of the XLMS dataset.

5. Line 203 “Rad26/CSB C bound the nucleosome efficiently (Supplementary Fig. 11, Supplementary Fig. 17) and retained the auto-inhibition of the nucleosome remodeling activity (Fig. 5d, Supplementary Fig. 15).”
This statement could be made clearer by referencing figure 5e and also mentioning that the retention of auto-inhibition activity is observed when compared to the Rad26/CSBΔN+ΔC. The Rad26/CSBΔN+ΔC mutant is only mentioned later.

6. Additional details on XLMS data collection, data processing and data analysis are required:
a. For data collection, what were the instrument settings? E.g. was it high resolution MS1/MS2 orbitrap data or was it low resolution MS2 ion trap data? Providing exact MS instruments settings would be helpful.
b. For data processing, details on how the database searches were done including database of proteins used and search parameters that deviate from the default, should be included.
c. For data analysis, what were the score-cutoffs used? How was this determined? Were the spectra manually inspected? Was both deuterated and non-deuterated forms considered as part of the analysis?
d. From experience, using non-cleavable DSS crosslinker in combination with low-resolution ion trap data and the xQuest pipeline can lead to rather high false positives. Reported peptides should ideally be manually inspected for key fragment ions.

7. Apart from Figure 4, perhaps there should be a list /table of identified crosslinks with formatted data analysis outputs from xQuest in the Supplementaries.

8. While I can see that the dataset PXD070143 has been deposited, the link in the data availability statement is incorrect and doesn’t work.

Version 2:

Reviewer comments:

Reviewer #1

(Remarks to the Author)
The authors have addressed my concerns in the revised manuscript, and I am now pleased to support its publication.

Reviewer #6

(Remarks to the Author)
All major concerns have been addressed. Thank you.

I only have one remaining minor point:
p.5, ll. 123-130. I do not think the use of the sum length of K27-G34 (with G34 as the center) + DSS spacer arm + two lysines as the maximum distance is suitable. I think that K27 as the centre with 30A maximum distance will be more appropriate. The current methodology would imply that the DSS crosslink is attached to G34 rather than K27.
Alternatively, using a residue BEFORE K27 as the centre (in combination with the above methodology using peptide backbone sum lengths up to K27) could be acceptable too.
In additional, given the general flexibility of the histone tail, the upper limit of 30A could be a little more flexible.
I recommend accepting the article after minor revision.

Open Access This Peer Review File is licensed under a Creative Commons Attribution 4.0 International License, which permits use, sharing, adaptation, distribution and reproduction in any medium or format, as long as you give appropriate credit to the original author(s) and the source, provide a link to the Creative Commons license, and indicate if changes were made.
In cases where reviewers are anonymous, credit should be given to 'Anonymous Referee' and the source.
The images or other third party material in this Peer Review File are included in the article's Creative Commons license, unless indicated otherwise in a credit line to the material. If material is not included in the article's Creative Commons license and your intended use is not permitted by statutory regulation or exceeds the permitted use, you will need to obtain permission directly from the copyright holder.
To view a copy of this license, visit https://creativecommons.org/licenses/by/4.0/

REVIEWER COMMENTS

Reviewer #1 (Remarks to the Author):

Comment)

In this manuscript, Fukushima et al. present the cryo-EM structure of Rad26 in complex with the nucleosome, providing insights into its role in transcription-coupled nucleotide excision repair. The authors combine a cryo-EM reconstruction with an AlphaFold-predicted model to depict how Rad26 recognizes the nucleosome and potentially functions as a chromatin remodeler within the ATP-dependent remodeling family. Notably, they highlight that Rad26 exhibits a distinct mode of nucleosome engagement compared to other remodelers, and propose a potential role for the KR-loop in recognizing RNA polymerase II and mediating nucleosome binding and remodeling. While the structural data reasonably support the authors' claims regarding Rad26's unique remodeling mechanism and the involvement of the KR-loop, the manuscript contains several inconsistencies and unclear interpretations that must be addressed. Therefore, revision is recommended to strengthen the scientific rigor and clarity of the study.

Detailed recommendations for revision are provided below. These improvements are essential to enhance the manuscript's readability and to advance understanding of Rad26's biological function.

Reply)

Thank you very much for this favorable comment. We have addressed this reviewer's comments, as listed below.

Major points

Comment)

1. The Coulomb density maps of Rad26 shown in Extended Data Fig. 4e and Fig. 1a appear notably different in quality. It seems the resolution for Rad26 may not be sufficient to confidently trace even the backbone. Could the authors clarify how the map in Fig. 1a was generated? Cryo-EM structures of nucleosome–protein complexes often exhibit higher resolution for the nucleosome core, while the binding partner tends to be less well-resolved. If focused refinement was performed specifically on Rad26, please report the corresponding gold-standard FSC resolution derived from that focused refinement.

Reply)

Thank you very much for this comment. We applied local sharpening using DeepEMhancer in Fig. 1a, and added a corresponding description in the figure legend in the revised manuscript.

Comment)

2. Were the contacting residues of the KR loop in DNA versus H3 tail recognition distinguishable in your map? The KR loop appears to interact with both DNA and the histone H3 tail, but the specific residues involved are not clearly described. If the resolution does not permit unambiguous assignment of these contacts, this limitation should be explicitly noted in the main text.

Reply)

Thank you very much for this helpful comment. Because the resolutions of both the KR loop and H3 tail do not allow unambiguous visualization of amino acid side chains, we cannot definitively assign the specific contacting residues. We have now explicitly noted this limitation in the main text (pp.4-5, ll.108-110) and the legend for the cryo-EM map of the KR loop in Supplementary Fig. 5b.

Comment)

3. The claim that “Our cryo-EM analysis confirmed that Rad26/CSBΔN binds to the SHL±6 position of the nucleosome, and the N-terminal deletion minimally affects the structure of the Rad26/CSB-nucleosome complex” is not fully convincing. The density maps presented in Extended Data Fig. 8d and f are not of sufficient quality to support this conclusion without ambiguity. Additional clarification or supporting evidence is needed to substantiate this structural comparison.

Reply)

Thank you very much for this valuable comment. Because of the limited resolution of the Rad26/CSBΔN map, we could not directly compare it with the Rad26/CSB full-length structure at the original resolutions. To enable a fair comparison, we applied low-pass filtering to 15 Å for both EM maps, thereby equalizing their resolution, and then compared them (Supplementary Fig. 7h). This analysis confirmed that Rad26/CSBΔN binds to the same nucleosomal position (SHL±6) as Rad26/CSB WT. Thus, the N-terminal deletion minimally affects the overall architecture of the Rad26/CSB-nucleosome complex.

Minor points

Comment)

1. Have you observed any particle populations with a 1:2 stoichiometry of nucleosome to Rad26? Given that Rad26 is reported to bind at both the +6 and –6 superhelical locations (SHLs), one might expect to detect complexes with two Rad26 molecules bound to a single nucleosome. If not, any possible explanation?

Reply)

Thank you very much for this comment. Upon carefully inspecting the 3D classification results, we did identify a class in which two Rad26 molecules appeared to bind to a single nucleosome. However, the EM density corresponding to the second Rad26 was too weak to allow reliable structural analysis, and thus we could not generate a suitable EM map for the two-Rad26-bound complex. These new data are presented in the new Supplementary Fig. 4g, and described in the text (p.4, ll.86-88).

Comment)

2. V633-H634-A635-K636-R637-R638-S639-K640-K641-D642 is hard to read. 633-VHAKRRSKKD-642 might be easier to look at the peptide sequence at the KR loop.

Reply)

Thank you very much for this helpful suggestion. To improve the readability, we have revised the notation to 633–VHAKRRSKKD–642 in the revised manuscript.

Comment)

3. In Fig. 3b, the colors used for the H3 tail, lobe 1, and the KR loop appear quite similar, which may lead to confusion. To improve clarity, please consider using distinct color schemes for each feature to clearly differentiate them.

Reply)

Thank you very much for this helpful suggestion. We changed the colors for lobe 1 and the KR loop to enhance the color differences with the H3 tail. We also labeled the brace helix in the revised Fig. 3b.

Comment)

4. Although the authors state that the KR loop is conserved from yeast to humans,

Extended Data Fig. 6a does not clearly support this claim. As the alignment data are not easily interpretable in their current form, please consider incorporating the relevant conservation information of Extended Data Fig 6a directly into Fig. 3a to allow readers to independently assess the degree of conservation.

Reply)
Thank you very much for this helpful suggestion. To improve the clarity, we moved the amino acid sequence alignment from Extended Data Fig. 6a to Fig. 3a, thereby allowing readers to directly assess the conservation of the KR loop across species.

Comment)
5. In Extended Data Fig. 10, please label the panels as “Replicate of Fig. 3e #2” and “Replicate of Fig. 3e #3” to prevent potential confusion for readers.

Reply)
We have revised Supplementary Fig. 13 (formerly Extended Data Fig. 10) and labeled the panels as “Replicate of Fig. 3e #2” and “Replicate of Fig. 3e #3”. The same labeling has been applied consistently to all replicate experiments.

Reviewer #2 (Remarks to the Author):
Comment)
The manuscript presents high-quality structural work on the Rad26/CSB–nucleosome complex, offering new insights into how CSB engages chromatin during transcription-coupled nucleotide excision repair (TC-NER). The cryo-EM data are compelling and well analyzed. However, to fully appreciate the regulatory and mechanistic aspects of CSB/Rad26 function, the manuscript would benefit from greater integration of known and emerging roles of the N- and C-terminal regions of CSB, especially in relation to its interaction with RNA polymerase II (RNAPII) and other TC-NER factors. Below are specific suggestions to expand the mechanistic depth of the work.

Reply)
Thank you very much for this favorable comment. We have addressed this reviewer’s comments, as listed below.

Comment)
1. N-terminal Leucine Latch Motif: Autoinhibitory Function
The study mentions that the N-terminal region (NTR) of Rad26 includes an auto-inhibitory domain with a leucine latch motif (LxxL). While this motif is functionally significant (as its deletion activates remodeling activity), structural and mechanistic details of its interaction with the ATPase domain (Lobe 1 and Lobe 2) remain insufficiently explored in this work.

Reply)
Point-by-point responses are listed below.

Recommendation 1)
Please expand your discussion on the regulatory role of the leucine latch. Incorporating structural modeling or mutagenesis experiments (L7G/L10G mutations) to test interaction with the ATPase domain would support a mechanistic understanding of auto-inhibition.

Reply)
Thank you very much for this valuable comment. To address the regulatory role of the leucine latch, we generated Rad26/CSB mutants in which Leu8 and Leu11 (corresponding to Leu7 and Leu10 of *S. pombe* Rhp26) were replaced by glutamine residues (Rad26/CSB L8Q/L11Q). Glutamine was selected because it is of comparable size but has a hydrophilic side chain, providing a functional contrast to leucine. We also purified Rad26/CSBΔC lacking the C-terminal coupling motif. In addition, we prepared the Rad26/CSB L8Q/L11Q+ΔC and ΔN+ΔC mutants. We then performed the nucleosome remodeling assay comparing Rad26/CSB WT, Rad26/CSB L8Q/L11Q, Rad26/CSBΔC, Rad26/CSB L8Q/L11Q+ΔC, and Rad26/CSB ΔN+ΔC. We also performed the XL-MS analyses with Rad26/CSB WT, Rad26/CSB L8Q/L11Q, and Rad26/CSBΔC bound to nucleosomes in the presence and absence of ATP or AMP-PNP. These results are presented in Fig. 4 and Fig. 5d,e. We discuss these new data in the new Results section “The N-terminal and C-terminal regions of Rad26/CSB” (p.5, ll.122-125, ll.135-144, p.6, ll.179-215).

Recommendation 2)
Further, crosslinking mass spectrometry (XL-MS) could be used to identify residues in

the N-terminal region that contact lobe 2 in both nucleosome-bound and apo states in presence or absence AMP-PNP.

Reply)
As mentioned above, we performed the XL-MS analyses comparing Rad26/CSB WT, Rad26/CSB L8Q/L11Q, and Rad26/CSBΔC bound to nucleosomes in the presence and absence of ATP or AMP-PNP. These new results are presented in Fig. 4 and described in the new Results section "The N-terminal and C-terminal regions of Rad26/CSB" (p.6, ll.179-215).

Recommendation 3)
The C-terminal region CSB-specific coupling motif of Rhp26 is essential for chromatin remodeling (Wang et al., PNAS, 2014). This study doesn't explore this important point. Wang et al., PNAS, 2014 showed that the remodeling activity of Rhp26 7P/10P leucine latch mutant is greatly reduced by H3 and H4 tail deletion.

Reply)
Thank you very much. In the revised manuscript, we carried out chromatin remodeling assays to compare the remodeling activities of Rad26/CSB WT, Rad26/CSB L8Q/L11Q, Rad26/CSBΔC, Rad26/CSB L8Q/L11Q+ΔC, and Rad26/CSB ΔN+ΔC. The results of these chromatin remodeling assays are presented in the new Fig. 5, and are extensively described in the new Results section "The N-terminal and C-terminal regions of Rad26/CSB" (p.6, ll. 179-215).

Recommendation 4)
The activity of Rad26 leucine latch mutant should be investigated with H3/H4 tail less nucleosomes.

Reply)
To address the role of histone tails, we attempted to prepare tailless H3 and H4 histones. While tailless H4 proved unstable and could not be purified, we successfully purified tailless H3 and reconstituted nucleosomes containing tailless H3. The nucleosome remodeling assay of Rad26/CSB WT was then performed with the tailless H3 nucleosome. We found that Rad26/CSB is proficient in the nucleosome remodeling, suggesting that the H3 N-terminal tail may not be involved in the functional Rad26/CSB-nucleosome interaction (Supplementary Fig. 10). In contrast, the

nucleosome remodeling activity of the Rad26 leucine latch mutant was drastically enhanced as compared to wild-type Rad26/CSB. These new results are also presented in the new Fig. 5d,e, and Supplementary Fig. 15, and described in the text (pp. 6-7, ll.180-189).

Comment)
2. Residue Overlap Between RNAPII and ATPase/Nucleosome Interactions
The N-terminal domain of CSB is also known to interact with stalled RNAPII, yet it is unclear whether this involves the same residues as those mediating auto-inhibition or nucleosome binding.

Recommendation)
Clarify whether shared residues in the N-terminal region, possibly within or near the leucine latch, are involved in both RNAPII engagement and ATPase inhibition. This could be tested using comparative XL-MS of Rad26/CSB in complex with either RNAPII or nucleosome. Distinct or overlapping footprints would strongly inform models of CSB conformational switching.

Reply)
Thank you very much for this insightful comment. To clarify whether residues in the N-terminal region of Rad26/CSB contribute to both RNAPII engagement and ATPase/nucleosome interactions, we conducted XL-MS analyses of Rad26/CSB in complexes with the nucleosome and RNAPII stalled on nucleosomes. The results of these comparative XL-MS experiments, which highlight potential overlaps and distinctions in the interaction footprints between Rad26/CSB and the nucleosome, are presented in Fig. 4c,g. In fact, our XL-MS analysis suggested that the Rad26/CSB-nucleosomal H3 interaction may be changed by RNAPII when it is stalled on the nucleosome. These new results are presented in the new Fig. 4c,g, and discussed on p.7, ll.190-201.

Comment)
3. C-terminal Domain: Role in RNAPII Interaction and Transcriptional Regulation
As demonstrated in Weems et al. (J. Biol. Chem., 2021), the C-terminal domain of CSB is essential for stable interaction with RNAPII and recruitment of the Elongin-CUL5 ubiquitin ligase complex, even under transcriptional stress (e.g., ER stress, hormone signaling), not just DNA damage.

Recommendation)
I recommend including the C-terminal domain in your structural or functional model, particularly to contextualize CSB's dual role in DNA repair and RNAPII transcription regulation. Functional assays using C-terminal truncation mutants could help delineate its role in remodeling vs. transcriptional engagement.

Reply)
Thank you very much. In the revised manuscript, we extensively carried out chromatin remodeling assays to compare the remodeling activities of Rad26/CSB WT, Rad26/CSB L8Q/L11Q, Rad26/CSBΔC, Rad26/CSB L8Q/L11Q+ΔC, and Rad26/CSB ΔN+ΔC. The results of these chromatin remodeling assays are now included in the new Fig. 5d,e. In addition, we conducted the XL-MS analyses with Rad26/CSB L8Q/L11Q and Rad26/CSBΔC in complexes with the nucleosome. We also performed the XL-MS analysis with the Rad26/CSB-nucleosome complex transcribed by RNAPII. These new data are presented in the new Fig. 4, and are discussed in the last paragraph of "The KR loop of the Rad26/CSB Lobe 2 domain" section and the new section "The N-terminal and C-terminal regions of Rad26/CSB" in the Results section (p.7, ll.202-215).

Comment)
4. Dynamic Competition Between RNAPII and Nucleosomes
The manuscript suggests Rad26 may function at both RNAPII-stalled complexes and nucleosomes during TC-NER, implying a dynamic exchange or handoff mechanism.
Recommendation:
I encourage the authors to perform time-resolved or competitive binding assays (e.g., FRET, biolayer interferometry) to assess whether RNAPII and nucleosomes bind Rad26/CSB sequentially or competitively. This would provide mechanistic support to your proposed model and clarify whether a mutually exclusive binding mode exists.

Reply)
Thank you very much for this insightful comment. We fully agree that time-resolved or competitive binding assays would provide valuable mechanistic insights into whether RNAPII and nucleosomes engage Rad26/CSB sequentially or competitively. Although such experiments are beyond the scope of the current study and would require

extensive optimization over a longer time frame, we recognize their importance and plan to pursue them in our future work.

Comment)

Final Recommendation

This manuscript presents a valuable structural contribution to our understanding of rad26/CSB function. To maximize its impact, I recommend a more integrated discussion of the N-terminal leucine latch motif, the C-terminal domain's role in transcriptional regulation, and emerging mechanistic insights from recent structures of RNA polymerase II (Pol II)–stalled complexes.

A key question that remains insufficiently addressed is how Rad26/CSB transitions between Pol II– and nucleosome-bound states, a process central to its function in transcription-coupled nucleotide excision repair (TC-NER). Elucidating this switch in binding modes through further discussion and targeted experimentation would strengthen the conceptual framework of the study.

Moreover, several mechanistic conclusions would benefit from additional validation, such as mutagenesis or crosslinking experiments, to support proposed models of autoinhibition and activation.

Reply)

We thank the reviewer for the valuable feedback. In response, we have revised the manuscript to more clearly integrate the roles of the N-terminal leucine latch motif and the C-terminal domain. We have also expanded the discussion of the Rad26/CSB transitions between the RNAPII- and nucleosome-bound states, and added new mutagenesis and crosslinking data to further support our proposed models of autoinhibition and activation.

Reviewer #3 (Remarks to the Author):

Comment)

Rad26, a yeast homologue of mammalian Cockayne syndrome protein B (CSB), plays an essential role in transcription-coupled nucleotide excision repair. In addition, Rad26 also possesses nucleosome remodeling activity, but the mechanism is unclear. In this manuscript, Fukushima, Kurumizaka and colleagues took a combined biochemistry and cryo-EM structural approach to visualize the structure of the Rad26/CSB-nucleosome complex. The quality of cryoEM structure is elegant. They found that

Rad26/CSB preferentially binds to the SHL±6 position of the nucleosomal DNA and distorts the entry/exit region of the nucleosome. The ATPase Lobe 1 and Lobe 2 domains bind to the nucleosome in the reverse orientation as compared to the Snf2 and Ino80 bound to the SHL±6 position. Finally, they found that the KR loop in Lobe 2 domain can function in nucleosome binding and remodeling, which is further supported by mutational analysis. Together, this study provides important insights into the remodeling function of Rad26 and suggest its potential functional role in transcription-coupled nucleotide excision repair. I have a few minor concerns that should be addressed before publication.

Comment)
1. In the results part, the authors found that the KR loop directly contacts the DNA at the SHL±6 position and the N-terminal tail of H3. This result is consistent with previous publication indicating the important roles of H3 and H4 tails for remodeling activity of Rad26 (Ref 22. Wang W et al PNAS 2019). On the other hand, the biochemical assay results (Fig 3e) in this manuscript show that Rad26 appears to exhibit higher affinity for H3 tail-less nucleosomes than WT nucleosomes, which appears to conflict with the structural observation of an interaction between the H3 tail and the KR loop of Rad26. How can this discrepancy be explained?

Reply)
Thank you very much for this important comment. Although our structural data show that the KR loop is located near the H3 tail at the SHL±6 position, the biochemical assays indicate that Rad26/CSB still efficiently binds to H3 tail-less nucleosomes and promotes the nucleosome remodeling. These results suggest that the interaction between the H3 tail and the KR loop is not essential for the Rad26/CSB activity. However, our XL-MS analysis suggested that the Rad26/CSB KR loop is located near the N-terminal tail of the nucleosomal histone H3. Intriguingly, the crosslinks between Rad26/CSB and the H3 N-terminal tail become more evident in the presence of RNAPII, suggesting that RNAPII may actively support the Rad26/CSB relocation to the nucleosome. These new findings are extensively discussed in the revised manuscript (p.5, ll.135-137).

Comment)
2. In the discussion section, the authors propose an interesting model that upon Rad26 recruit to arrested Pol II, then transfer to nucleosome ahead of Pol II and evicted the

nucleosome and clear the room for TC-NER. While this is very provocative showing the role of remodeling activity of Rad26 in TC-NER, mammalian CSB also plays an important role to recruit downstream factors including CSA, UVSSA, and TFIIH. The relative position of Pol II-CSB/Rad26 are critical for these recruitment events. It remains unclear how this new model (proposed in this manuscript) will be integrated into current model of TC-NER. In addition, for normal elongation for a set of genes (no lesion) or with small lesions, previous publication suggests that CSB forms a stable complex with Pol II and acts as an ATP-dependent processivity factor, promoting Pol II progression through a nucleosome barrier (and/or small lesions) and promotes elongation on these genes (Xu J et al PNAS 2020). This previous model may be also integrated with current model to elucidate the roles of Rad26 (on lesion recognition) in both TC-NER and transcription elongation for certain genes.

Reply)
We thank the reviewer for this insightful comment. We agree that integrating our proposed model with the current framework of TC-NER and transcription elongation is essential. In the revised Discussion section, we have extensively considered how the Rad26/CSB-RNAPII-nucleosome arrangement could accommodate the recruitment of CSA, UVSSA, and TFIIH, and how the transition of Rad26/CSB from RNAPII to the nucleosome might facilitate these downstream events in TC-NER. We have also discussed how our model can be reconciled with the previous finding that CSB acts as an ATP-dependent processivity factor during normal transcription elongation (Xu et al., *PNAS* 2020). We now propose that Rad26/CSB may adopt distinct functional states—serving as a processivity factor under non-damaged conditions and as a remodeling factor upon lesion-stalled RNAPII—to coordinate both transcription elongation and TC-NER.

Comment)
3. For Fig. 3b, it would be easier for readers to better understand the interactions between H3 and Rad26-KR-loop by showing the side chains of KR loop and their interaction partners in H3 tail and DNA.

Reply)
Thank you very much for this helpful comment. Because the resolutions of both the KR loop and the H3 tail do not allow unambiguous visualization of amino acid side chains,

we cannot definitively assign the specific contacting residues. We have now explicitly noted this limitation in the main text (pp.4-5, ll.108-110).

Comment)
4. Line 125 “This suggest that the N-terminal…”, “This suggest” should be “This suggests”. It would be great for authors take a careful look at the grammar and spelling to ensure clarity.

Reply)
Thank you very much for this helpful suggestion. We have corrected this error and carefully checked the manuscript for other grammatical and spelling issues in the revised version.

Reviewer #4 (Remarks to the Author):
Comment)
I co-reviewed this manuscript with one of the reviewers who provided the listed reports. This is part of the Nature Communications initiative to facilitate training in peer review and to provide appropriate recognition for Early Career Researchers who co-review manuscripts.

Reviewer #5 (Remarks to the Author):

I co-reviewed this manuscript with one of the reviewers who provided the listed reports. This is part of the Nature Communications initiative to facilitate training in peer review and to provide appropriate recognition for Early Career Researchers who co-review manuscripts.

Comments:
In this manuscript, Fukushima, Kurumizaka and colleagues took use of biochemistry and cryo-EM structural methods to visualize the structure of the Rad26/CSB-nucleosome complex. They found that Rad26/CSB preferentially binds to the SHL±6 position of the nucleosomal DNA and distorts the entry/exit region of the nucleosome. The ATPase Lobe 1 and Lobe 2 domains bind to the nucleosome in the reverse orientation as compared to the Snf2 and Ino80 bound to the SHL±6 position. Finally, they found that the KR loop in Lobe 2 domain can function in nucleosome binding and

remodeling. This work paves the way to clarify the role of Rad26/CSB in TC-NER.
I have a few minor concerns that should be addressed before publication.

Comment)
1. In the results part, the authors found that the KR loop directly contacts the DNA at the SHL±6 position and the N-terminal tail of H3. However, the EMSA results showed that Rad26/CSB exhibited higher affinity to the H3 tail-less nucleosome than the wild-type nucleosome, this result suggests that the N-terminal tail of H3 may negatively regulate the Rad26/CSB binding to the nucleosome. The interactions between Rad26 and N-terminal tail of H3 may be an artifact resulted from glutaraldehyde crosslink during sample preparation?

Reply)
Thank you very much for this valuable comment. While our structural analysis shows that the KR loop is located near the H3 N-terminal tail, our EMSA results indicate that Rad26/CSB binds efficiently to H3 tail-less nucleosomes, suggesting that the H3 tail is not essential for the stability of Rad26/CSB-nucleosome binding. Although we cannot eliminate the possibility that the interactions between Rad26 and the N-terminal tail of H3 may be an artifact resulting from glutaraldehyde crosslinking during sample preparation, in the revised manuscript, our XL-MS analysis suggested that the Rad26/CSB KR loop resides near the N-terminal tail of the nucleosomal histone H3. Intriguingly, the crosslinks between Rad26/CSB and the H3 N-terminal tail become more evident in the presence of RNAPII, suggesting that RNAPII may actively support the Rad26/CSB relocation to the nucleosome. These new findings are extensively discussed in the revised manuscript (p.5, ll.135-144).

Comment)
2. Did the authors conduct restriction enzyme accessibility assay with H3 tail-less nucleosome to study the interplay between nucleosome and Rad26/CSB-H3 tail-less nucleosome

Reply)
Thank you very much for this valuable comment. We performed a chromatin remodeling assay of Rad26/CSB with the H3 tail-less nucleosome. The results are presented in Supplementary Fig. 10 and described on p.6, ll.166-170.

Comment)
3. In the discussion section, the authors formulated a hypothesis that Rad26/CSB relocates from RNAPII to the nucleosome after recruitment of NER proteins during TC-NER process. Is there any related literatures or research to support this hypothesis? Since we could also assume that another Rad26/CSB or other remodelers is recruited to the nucleosome to slide the nucleosome and help to expose the DNA lesion.

Reply)
We thank the Reviewer for this comment. In the revised Discussion section, we have elaborated on how the Rad26/CSB-RNAPII-nucleosome arrangement could accommodate the recruitment of CSA, UVSSA, and TFIIH, and how the transition of Rad26/CSB from RNAPII to the nucleosome might facilitate these downstream events in TC-NER. We have also discussed how our model can be reconciled with the previous finding that CSB acts as an ATP-dependent processivity factor during normal transcription elongation. We now propose that Rad26/CSB may adopt distinct functional states—serving as a processivity factor under non-damaged conditions and as a remodeling factor with lesion-stalled RNAPII—to coordinate both transcription elongation and TC-NER.

Comment)
4. An alternative model suggests that CSB forms a stable complex with Pol II and acts as an ATP-dependent processivity factor, promoting Pol II progression through a nucleosome barrier. It would be helpful if the authors could comment on how this model compares to the one presented in this study.

Reply)
We appreciate this insightful comment. This point is addressed in the Discussion section, where we compare our model with the proposed processivity model and note that these mechanisms are not mutually exclusive. We explain that CSB likely functions as an ATP-dependent processivity factor during normal transcription, while in TC-NER, it may transition to remodel the nucleosome after recruiting repair factors to the stalled RNAPII.

Comment)
5. Line 125 “This suggest that the N-terminal…”, “This suggest” should be “This suggests”, the authors may want to take another look at the grammar and spelling to ensure clarity.

Reply)
Thank you very much for this helpful suggestion. We have corrected this error and carefully checked the manuscript for other grammatical and spelling issues in the revised version.

REVIEWER COMMENTS

Reviewer #1 (Remarks to the Author):

Although authors claimed that “In the Rad26/CSB-nucleosome complex, the K636, R637, R638, K640, and K641 residues of the KR loop directly contact the DNA at the SHL±6 position (page 5, lines 113-115)”, the structural data in Fig. 3b and Supplementary Fig. 5b instead show that the KR loop primarily interacts with the H3 tails rather than with nucleosomal DNA. Mutating the KR loop to GS residues disrupted nucleosome binding, which the authors interpreted as evidence for direct engagement of the KR loop with nucleosomal DNA. However, the authors did not rule out an alternative explanation: the Rad26–nucleosome dissociation could also result from disrupting KR loop–H3 tail interactions. This alternative model also provides a plausible explanation for why H3-tail–less nucleosomes bind more strongly to Rad26—removal of the H3 tail would eliminate interference with KR loop–H3 tail interactions, allowing the KR loop to access nucleosomal DNA more directly.
This interpretation challenges the authors’ main conclusion that “the KR loop-H3 tail interaction is not essential for Rad26/CSB nucleosome binding and remodeling. (Page 5, line 131)”. Because the role of the KR loop in nucleosome binding is a central claim of the manuscript, the authors should unambiguously clarify this issue before publication.

Reply)
Thank you for this insightful comment. As this reviewer suggested, a plausible explanation for why H3-tail-less nucleosomes bind more strongly to Rad26 described in the revised manuscript (p.5, ll. 139-144).

In Supplementary Fig. 7d, Rad26ΔN (orange) appears to make extensive contacts with the H2A–H2B surface, a feature not observed in the Rad26–nucleosome structure (Fig. 1a). Supplementary Fig. 7h further suggests that this region exhibits distinct EM densities between the Rad26–nucleosome and Rad26ΔN–nucleosome complexes.

Reply)
Thank you for this comment. In the revised manuscript, we described this finding in the revised manuscript (p.6, ll.165-166).

In Fig. 4a, Lys611 (Rad26) and H3K27 appear too far apart to support direct crosslinking in the XL-MS analysis.

Reply)
To ensure the possibility of the H3K27-Rad26K611 crosslinking, in the Rad26-nucleosome structure, we measured the distance range (51.35Å) with the visible H3G34 position as the center. The 51.35Å length is the sum of lengths H3K27-G34, spacer DSS, and two lysin sidechains. We then confirmed that H3K27 and Rad26K611 are located within the possible distance for crosslinking in the cryo-EM structure. These facts are described in the revised manuscript (p.5, ll. 124-130) and presented in new Figure 4b, d.

In Supplementary Fig. 7h, the legend should include an explanation of the dark triangle annotation.

Reply)
An explanation of the dark triangle annotation was included in the Supplementary Fig. 7h legend.

Reviewer #2 (Remarks to the Author):

All substantive concerns have been thoroughly addressed, and the revised manuscript is greatly improved. I have only one remaining minor point:
Histone H3 residues 35–39: As there is no strong supporting cryo-EM density for these residues, please remove them from the model. I think the density in that area is the contribution of R638 and other neighboring residues (636KRRSKK641) of chain K.

With this minor correction, the manuscript is suitable for publication.
Recommendation: Accept after minor revision.

Reply)
Thank you very much for your positive evaluation and helpful comment. Although the local resolution around histone H3 residues 34-39 is relatively low due to flexibility, the corresponding EM density becomes clearly visible when the map is low-pass filtered to

6 Å. This density is consistent with the positioning of H3 residues 34-39 in our model. To clarify this point, we have added the 6 Å low-pass filtered map to the revised manuscript as new Supplementary Fig. 5c.

Reviewer #3 (Remarks to the Author):

The revised manuscript has addressed all questions raised from previous version. Therefore I recommend it for publication.

Reply)
Thank you very much.

Reviewer #4 (Remarks to the Author):

I co-reviewed this manuscript with one of the reviewers who provided the listed reports. This is part of the Nature Communications initiative to facilitate training in peer review and to provide appropriate recognition for Early Career Researchers who co-review manuscripts.

Reply)
Thank you very much.

Reviewer #5 (Remarks to the Author):

I co-reviewed this manuscript with one of the reviewers who provided the listed reports. This is part of the Nature Communications initiative to facilitate training in peer review and to provide appropriate recognition for Early Career Researchers who co-review manuscripts.

Reply)
Thank you very much.

Reviewer #6 (Remarks to the Author):

Comments to the Authors:
Overview:
Fukushima et al. presents a large body of work combining remodelling assays, cryoEM and XLMS to investigate the structure and role of Rad26 in TC-NER. The presented data largely supports their claims. However, additional information should be included and reviewed) with regards to the XLMS data prior to publication.
The cryoEM component has been extensively reviewed and commented on. The comments below will mostly focus on the new XLMS data.

1. Line142 “This enhancement of the Rad26/CSB-H3 interaction by RNAPII may function to stimulate the Rad26/CSB transfer from RNAPII to the nucleosome during the TC-NER process (discussed later).”
This statement seems rather speculative based on the presented XLMS data. Is there additional data to support this statement?

Reply)
Thank you very much. As this reviewer suggested, we deleted the corresponding sentences including this sentence in the revised manuscript.

2. It is noted from Figure 4g that there appears to be an enhancement of N-terminal to C-terminus crosslinking of Rad26 in the presence of RNAPII.
Do the authors have any comments/insights on this observation? Do these crosslinks fit with the proposed model in Figure 6?

Reply)
In the previous Figure 4c, g (current Figure 4f), we analyzed only H3-Rad26 interactions, although the RNAPII and transcription elongation factors were presented under the experimental condition. As this reviewer suggested by the comment 3, we re-analyzed the XL-MS data including RNAPII subunits (RPB2 and RPB4), Spt4/5, Elf1, TFIIS, H3, and Rad26. We then obtained the new XL-MS interactions and are presented in the new Figure 4c, f, g. In the new Figure 4f, interactions between N-terminal and C-terminal regions of Rad26 were less than threshold, although they were observed when threshold value was decreased. All XL-MS data are deposited to JPOST.

3. Related to point 2 above, were there other observed crosslinks from Rad26 to RNAPII and other proteins? Do these support your proposed model or provide additional insight?

Reply)
We re-analyzed the XL-MS data including RNAPII subunits (RPB2 and RPB4), Spt4/5, Elf1, TFIIS, H3, and Rad26. We then observed the XL-MS interactions between Rpb2 and Rad26 (new Figure 4g). In the revised manuscript, we described this finding, and provided additional insight related to the cryo-EM structure (p.7, ll. 211-215).

4. How does all identified crosslinks map to the structure of Rad26 reported here, with and without nucleosome? Does the identified crosslinks fall within the crosslinker length of DSS? This can provide some insight into the quality of the XLMS dataset.

Reply)
We did not perform the XL-MS analysis without nucleosome. To ensure the possibility of the H3K27-Rad26K611 crosslinking, we measured the distance range (51.35Å) with the H3G34 position as the center. The 51.35Å length is the sum of lengths H3K27-G34, spacer DSS, and two lysin sidechains. We then confirmed that H3K27 and Rad26K611 are located within the possible distance for crosslinking in the cryo-EM structure. These facts are described in the revised manuscript (p.5, ll. 124-130) and are presented in new Figure 4b, d.

5. Line 203 “Rad26/CSBΔC bound the nucleosome efficiently (Supplementary Fig. 11, Supplementary Fig. 17) and retained the auto-inhibition of the nucleosome remodeling activity (Fig. 5d, Supplementary Fig. 15).”
This statement could be made clearer by referencing figure 5e and also mentioning that the retention of auto-inhibition activity is observed when compared to the Rad26/CSBΔN+ΔC. The Rad26/CSBΔN+ΔC mutant is only mentioned later.

Reply)
In the revised manuscript, we rewrote corresponding paragraph as suggested by this comment (p. 8, ll. 220-224).

6. Additional details on XLMS data collection, data processing and data analysis are

required:

a. For data collection, what were the instrument settings? E.g. was it high resolution MS1/MS2 orbitrap data or was it low resolution MS2 ion trap data? Providing exact MS instruments settings would be helpful.

Reply)

Crosslink MS analysis was performed according to the settings described in a previous study (Leitner et al., 2014). MS data were acquired using an Orbitrap mass spectrometer at a resolution of 60,000. MS/MS analysis was performed by ion selection with a quadrupole, followed by fragmentation by collision-induced dissociation (CID) and detection in an ion trap. We have added this explanation to Materials and Methods (p.15, ll. 496-499).

b. For data processing, details on how the database searches were done including database of proteins used and search parameters that deviate from the default, should be included.

Reply)

Data processing parameters were defined in the xmm.def file for isotopically labeled samples. The parameters were set as follows: Isopair_Mr_tolerance = 15, Isopair_Tr_tolerance = 3.0, Isotopeshift = 12.075321, and Printisotopicscanpairs = 1. For analyses of samples containing nucleosomes and Rad26 (Fig.4a, and e), a FASTA file comprising histones (H2A, H2B, H3.1, and H4) and Rad26 (WT, L8Q/L11Q, and ΔC) was used. For analyses of samples containing nucleosomes, Rad26, and RNA polymerase II (Fig.4c, f and g), a FASTA file containing histone H3, Rad26 (WT), RNA polymerase II subunits (Rpb2 and Rpb4), and transcription factors (Spt4, Spt5, Elf1, and TFIIS) was used. We have added this explanation to Materials and Methods (p.15, ll. 499-506).

c. For data analysis, what were the score-cutoffs used? How was this determined? Were the spectra manually inspected? Was both deuterated and non-deuterated forms considered as part of the analysis?

Reply)

Approximately 1,500-3,300 crosslinked peptides were identified per analysis. To discuss crosslinked peptides identified with high confidence and a low likelihood of

false positives, only the top several dozen crosslinks with the highest LD scores were selected for further analysis (Fig. 4). In Figures 4a and 4e, all crosslinks with LD scores ≥30 are shown, whereas in Figures 4c, 4f, and 4g, all crosslinks with LD scores ≥18 are shown. We have added this explanation to Materials and Methods (p.15, ll. 506-511).

We note that the use of DSS-H12/D12, including both deuterated and non-deuterated forms, was already described in the original manuscript (p. 15, l. 477 and l. 486). We have also added an explanation of the data processing, specifying that the data processing parameters for isotopically labeled samples were defined in the xmm.def file (p. 15, ll. 499–500).

To eliminate confusion, we have removed the data obtained in the absence of AMP-PNP (formerly Figure 4f), as it yielded results comparable to those observed in the presence of AMP-PNP.

d. From experience, using non-cleavable DSS crosslinker in combination with low-resolution ion trap data and the xQuest pipeline can lead to rather high false positives. Reported peptides should ideally be manually inspected for key fragment ions.

Reply)
As described above, only crosslinks with high LD scores were included in the analysis, thereby minimizing the contribution of potential false positives.
To support our interpretation, we have provided, for the reviewer's reference, an additional figure showing the annotation of the crosslinked peptide sequences and the corresponding MS peaks (fragment ion m/z values) for the two crosslinks that are particularly important for the present discussion: the Rad26 intra-interaction suggesting autoinhibition (Fig. 4e, LD-Score: 31.83) and the Rad26–H3 inter-interaction (Fig. 4c, LD-Score: 18.05). In the MS spectra, peaks that match the crosslinked peptide fragments are marked with diamond symbols. A higher number of matched peaks corresponds to a higher Ld score.

xQuest results

SLESKINSR-ILQGKR-a5-b5

mz min. 85 mz max. 2000
MS2 tolerance for common-ions [Da] 0.2 MS2 tolerance for xlink-ions [Da] 0.3 ☐ logscale ☐ labels ☐ hide peptide structure

---

☐ water-loss ☐ NH3-loss ☐ allow matching of 2nd isotopic peak ☐ show table with matching ions ☐ show comparison of original spectra
☐ a ☐ x
☑ b ☑ y
☐ c ☐ z

reload

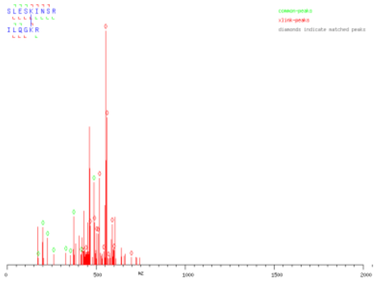

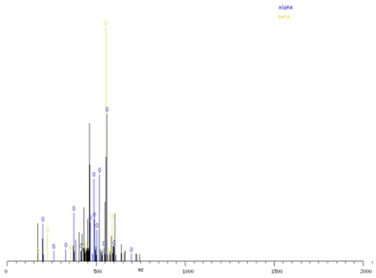

Only one spectrum was provided. Spectrum sorting cannot be applied. All peaks are matched against common- and xlink-ions

alpha-chain

alpha_common_b_standard_plus1	88.04	201.12	330.17	417.20	-	-	-	-	-
alpha_common_b_standard_plus2	44.52	101.07	165.59	209.10	-	-	-	-	-
alpha_common_b_standard_plus3	30.02	67.71	110.73	139.74	-	-	-	-	-
alpha_xlink_b_standard_plus2	-	-	-	-	698.91	755.45	812.48	855.99	934.04
alpha_xlink_b_standard_plus3	-	-	-	-	466.28	503.97	541.99	571.00	623.03
alpha_xlink_b_standard_plus4	-	-	-	-	349.96	378.23	406.74	428.50	467.52
AA	S	L	E	S	K	I	N	S	R
alpha_common_y_standard_plus1	-	-	-	-	-	489.28	376.19	262.15	175.12
alpha_common_y_standard_plus2	-	-	-	-	-	245.14	188.60	131.58	88.06
alpha_common_y_standard_plus3	-	-	-	-	-	163.76	126.07	88.06	59.05
alpha_xlink_y_standard_plus2	943.05	899.53	842.99	778.47	734.95	-	-	-	-
alpha_xlink_y_standard_plus3	629.03	600.02	562.33	519.31	490.30	-	-	-	-
alpha_xlink_y_standard_plus4	472.03	450.27	422.00	389.74	367.98	-	-	-	-

---

beta-chain

beta_common_b_standard_plus1	114.09	227.18	355.23	412.26	-	-
beta_common_b_standard_plus2	57.55	114.09	178.12	206.63	-	-
beta_common_b_standard_plus3	38.70	76.40	119.08	138.09	-	-
beta_xlink_b_standard_plus2	-	-	-	-	855.99	934.04
beta_xlink_b_standard_plus3	-	-	-	-	571.00	623.03
beta_xlink_b_standard_plus4	-	-	-	-	428.50	467.52
AA	I	L	Q	G	K	R
beta_common_y_standard_plus1	-	-	-	-	-	175.12
beta_common_y_standard_plus2	-	-	-	-	-	88.06
beta_common_y_standard_plus3	-	-	-	-	-	59.05
beta_xlink_y_standard_plus2	943.05	886.51	829.96	765.93	737.42	-
beta_xlink_y_standard_plus3	629.03	591.34	553.64	510.96	491.95	-
beta_xlink_y_standard_plus4	472.03	443.76	415.49	383.47	369.22	-

Matched common ions alpha:	7	Matched xlink ions alpha:	10	Matched ions alpha:	17
Matched common ions beta:	3	Matched xlink ions beta:	6	Matched ions beta:	9

xQuest results

KSAPSAAGGVK-ADVAKDLPK-a1-b5

mz min. 85 mz max. 2000
MS2 tolerance for common-ions [Da] 0.2 MS2 tolerance for xlink-ions [Da] 0.3 ☐ logscale ☐ labels ☐ hide peptide structure

☐ water-loss ☐ NH3-loss ☐ allow matching of 2nd isotopic peak ☐ show table with matching ions ☐ show comparison of original spectra
☐ a ☐ x
☑ b ☑ y
☐ c ☐ z

reload

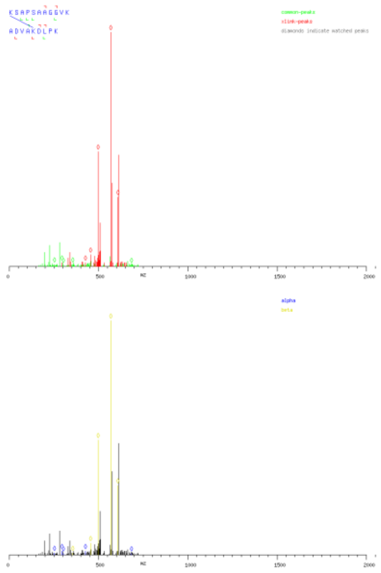

alpha-chain

alpha_xlink_b_standard_plus2	611.86	655.37	690.89	739.42	782.93	818.45	853.97	882.48	910.99	960.53	1024.57
alpha_xlink_b_standard_plus3	408.24	437.25	460.93	493.28	522.29	545.97	569.65	588.66	607.66	640.69	683.38
alpha_xlink_b_standard_plus4	306.43	328.19	345.95	370.21	391.97	409.73	427.49	441.74	456.00	480.77	512.79
AA	K	S	A	P	S	A	A	G	G	V	K
alpha_common_y_standard_plus1	-	844.45	757.42	686.38	589.33	502.30	431.26	360.22	303.20	246.18	147.11
alpha_common_y_standard_plus2	-	422.73	379.21	343.70	295.17	251.65	216.13	180.62	152.11	123.59	74.06
alpha_common_y_standard_plus3	-	282.16	253.15	229.47	197.12	168.10	144.43	120.75	101.74	82.73	49.71
alpha_xlink_y_standard_plus2	1033.58	-	-	-	-	-	-	-	-	-	-
alpha_xlink_y_standard_plus3	689.39	-	-	-	-	-	-	-	-	-	-
alpha_xlink_y_standard_plus4	517.29	-	-	-	-	-	-	-	-	-	-

beta-chain

beta_common_b_standard_plus1	72.04	187.07	286.14	357.18	-	-	-	-	-
beta_common_b_standard_plus2	36.53	94.04	143.57	179.09	-	-	-	-	-
beta_common_b_standard_plus3	24.69	63.03	96.05	119.73	-	-	-	-	-
beta_xlink_b_standard_plus2	-	-	-	-	797.94	855.46	912.00	960.53	1024.57
beta_xlink_b_standard_plus3	-	-	-	-	532.30	570.64	608.34	640.69	683.38
beta_xlink_b_standard_plus4	-	-	-	-	399.48	428.23	456.50	480.77	512.79
AA	A	D	V	A	K	D	L	P	K
beta_common_y_standard_plus1	-	-	-	-	-	472.28	357.25	244.17	147.11
beta_common_y_standard_plus2	-	-	-	-	-	236.64	179.13	122.59	74.06
beta_common_y_standard_plus3	-	-	-	-	-	158.10	119.76	82.06	49.71
beta_xlink_y_standard_plus2	1033.58	998.06	940.55	891.01	855.49	-	-	-	-
beta_xlink_y_standard_plus3	689.39	665.71	627.37	594.34	570.67	-	-	-	-
beta_xlink_y_standard_plus4	517.29	499.53	470.78	446.01	428.25	-	-	-	-

Matched common ions alpha:	4	Matched xlink ions alpha:	2	Matched ions alpha:	6
Matched common ions beta:	2	Matched xlink ions beta:	5	Matched ions beta:	7

7. Apart from Figure 4, perhaps there should be a list /table of identified crosslinks with formatted data analysis outputs from xQuest in the Supplementaries.

Reply)
We have added a list of crosslinked peptides identified by crosslinking mass spectrometry, corresponding to Fig. 4, as a new Supplementary Fig. 18. This figure presents the formatted data analysis output generated by xQuest/xProphet.

8. While I can see that the dataset PXD070143 has been deposited, the link in the data

availability statement is incorrect and doesn’t work.

Reply)

The dataset (PXD070143) has been placed on hold and will be made publicly available upon acceptance of the manuscript.

REVIEWER COMMENT

Reviewer #6 (Remarks to the Author):

Comment)

All major concerns have been addressed. Thank you.

I only have one remaining minor point:

p.5, ll. 123-130. I do not think the use of the sum length of K27-G34 (with G34 as the center) + DSS spacer arm + two lysines as the maximum distance is suitable. I think that K27 as the centre with 30A maximum distance will be more appropriate. The current methodology would imply that the DSS crosslink is attached to G34 rather than K27.

Alternatively, using a residue BEFORE K27 as the centre (in combination with the above methodology using peptide backbone sum lengths up to K27) could be acceptable too.

In additional, given the general flexibility of the histone tail, the upper limit of 30A could be a little more flexible.

I recommend accepting the article after minor revision.

Reply)

We appreciate the reviewer's thoughtful suggestion.

In our cryo-EM structural model, H3 G34 is the first residue that could be resolved, and therefore the structural position of K27 is not defined. For this reason, we estimated the theoretical distance from G34 back to K27 and used this to approximate the spatial region within which K27 could be located. This calculation yields a maximum distance of 51.35 Å. Because K27 is not present in the resolved structure and its position cannot be directly determined, we are unfortunately unable to define K27 as the center in our analysis. We agree that our original description may have been unclear, and we have revised the corresponding text in the manuscript to improve clarity (p.5, ll. 127-137).
